# Carrier multiplication in perovskite solar cells with internal quantum efficiency exceeding 100%

**DOI:** 10.1038/s41467-023-41758-w

**Published:** 2023-10-09

**Authors:** Yue Wang, Senyun Ye, Jia Wei Melvin Lim, David Giovanni, Minjun Feng, Jianhui Fu, Harish N S Krishnamoorthy, Qiannan Zhang, Qiang Xu, Rui Cai, Tze Chien Sum

**Affiliations:** 1 Division of Physics and Applied Physics, School of Physical and Mathematical Sciences, Nanyang Technological University, 21 Nanyang Link, Singapore, 637371 Singapore; 2 Centre for Disruptive Photonic Technologies, TPI, School of Physical and Mathematical Sciences, Nanyang Technological University, Singapore, 637371 Singapore; 3 Present Address: Tata Institute of Fundamental Research-Hyderabad, Sy. No. 36/P, Gopanapally Village, Serilingampally Mandal, Hyderabad, 500046 India

**Keywords:** Devices for energy harvesting, Solar cells

## Abstract

Carrier multiplication (CM) holds great promise to break the Shockley-Queisser limit of single junction photovoltaic cells. Despite compelling spectroscopic evidence of strong CM effects in halide perovskites, studies in actual perovskite solar cells (PSCs) are lacking. Herein, we reconcile this knowledge gap using the testbed Cs_0.05_FA_0.5_MA_0.45_Pb_0.5_Sn_0.5_I_3_ system exhibiting efficient CM with a low threshold of 2*E*_g_ (~500 nm) and high efficiency of 99.4 ± 0.4%. Robust CM enables an unbiased internal quantum efficiency exceeding 110% and reaching as high as 160% in the best devices. Importantly, our findings inject fresh insights into the complex interplay of various factors (optical and parasitic absorption losses, charge recombination and extraction losses, etc.) undermining CM contributions to the overall performance. Surprisingly, CM effects may already exist in mixed Pb-Sn PSCs but are repressed by its present architecture. A comprehensive redesign of the existing device configuration is needed to leverage CM effects for next-generation PSCs.

## Abstract (web summary)

While there has been evidence of strong carrier multiplication effects in halide perovskites, studies in actual solar cells are lacking. Here, the authors demonstrate such effects with a low threshold of 2*E*_g_ and a high efficiency of 99.4%, realizing unbiased internal quantum efficiency up to 160%.

## Introduction

Harvesting solar energy is one of the most sustainable approaches to satisfy Mankind’s ever-increasing energy demand. The recent spate of extreme weather events worldwide shows that no one is spared from climate change. Hence there is greater urgency to develop even more cost-effective and efficient solar cells. Perovskite solar cells (PSCs) have gained widespread interest due to their facile processibility and outstanding performance^1–10^. Within a decade of development, its power conversion efficiency (PCE) has surged from ~3.8 to >25%^11,12^, showing great promise to challenge the monopoly of Silicon cells. Regardless, the Shockley-Queisser (SQ) limit of a single junction solar cell is still limited to around 33%^13^. One of the main loss channels is the loss of the excess kinetic energies of hot carriers as heat in the absorber^14–17^. These hot carriers are formed from the absorption of high-energy photons. It is desirable to convert the excess energies, which would otherwise be normally lost as heat, into electrical work to boost efficiencies. Carrier multiplication (CM) or multiple exciton generation (MEG) enables efficiency enhancement by generating multiple electron-hole pairs (or excitons) from the absorption of a single high-energy photon. This provides an exciting possibility for surpassing the SQ limit to a theoretical maximum of 44.4%^18^.

Halide perovskites possess slow hot carrier cooling properties highly favorable for CM or MEG^19–24^. Low CM/MEG thresholds ($h{\nu }_{{{{{{\rm{th}}}}}}}$) and high CM/MEG quantum yields (QYs) were reported for FAPbI_3_ nanocrystals (NCs), CsPbI_3_ NCs, and (FASnI_3_)_0.6_(MAPbI_3_)_0.4_ bulk films^18,22–25^. However, most studies focused on transient absorption (TA) spectroscopy measurements conducted on bare perovskite films rather than in actual devices. A recent study reported efficient MEG in perovskite NCs, which enabled photodetector enhancements when excited in the ultraviolet (UV)/deep UV region^25^. However, the demonstration of MEG enhancements in actual PSC devices, which is another major application that will benefit from enhanced MEG, is still lacking. In this regard, an external quantum efficiency (EQE) or internal quantum efficiency (IQE) measured under a bias voltage in a photodetector may not be equivalent to the situation in a solar cell operating under zero bias. Hence, the demonstration of CM/MEG in a real-world PSC device and a thorough evaluation of whether such effects will contribute to tangible PSC enhancements is paramount for the development of next-generation photovoltaic technologies.

The key evidence of CM in a PSC device is an EQE or IQE surpassing 100% without any applied bias voltage or gain. For typical Pb-based perovskites with bandgaps around 1.7 eV, the CM threshold energy must be larger than 3.4 eV (<365 nm), which lies at the fringe of the most intense wavelength region of the solar spectrum^22,23^. Absorption of wavelengths below 400 nm by the perovskite layer is relatively weak because of absorption from the fluorine-doped tin oxide (FTO) or indium tin oxide (ITO) glass^26^. Thus, CM from these perovskite layers (if any) is unlikely to have a significant impact on PSC device performance. This is one plausible reason why CM has not been reported in PSC devices. The mixed Pb-Sn perovskite system with a narrower bandgap (~1.17–1.30 eV) is more suitable as its CM threshold energy is well-matched to the solar spectrum at its strongest intensity around 2.48 eV (500 nm)^24,27^.

Herein, we demonstrate efficient CM in the mixed Pb-Sn perovskite Cs_0.05_FA_0.5_MA_0.45_Pb_0.5_Sn_0.5_I_3_ (${E}_{{{{{{\rm{g}}}}}}}$ ~ 1.24 eV), which possesses an intrinsically low CM threshold of 2*E*_g_ and high CM efficiency of 99.4 ± 0.4%. Furthermore, we attain a peak IQE of ~161% (average 157 ± 4%) in the champion quartz-based PSC devices without applying any additional bias voltage or gain. Optical modeling further substantiates this finding of the IQE exceeding 100% at the CM threshold of 2*E*_g_.

## Results

### CM in mixed Pb-Sn perovskite films

**Fig. 1:**
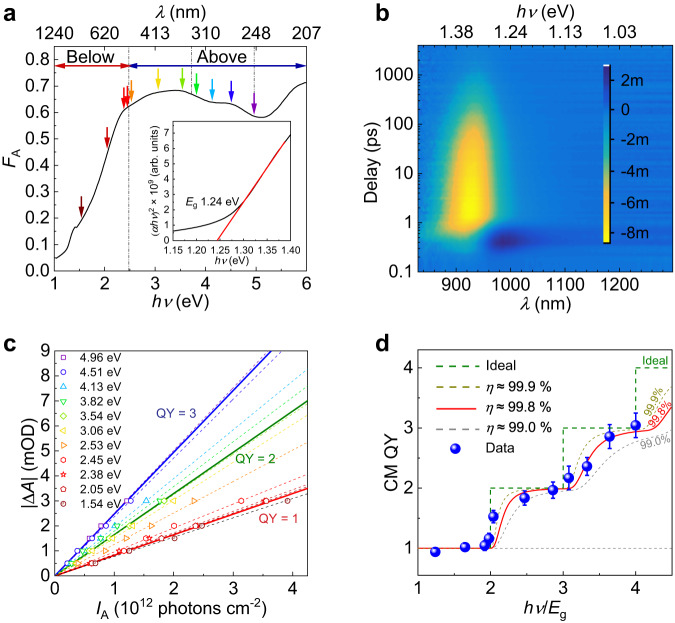
Optical spectroscopy of Cs_0.05_FA_0.5_MA_0.45_Pb_0.5_Sn_0.5_I_3_. **a** Steady-state absorption spectrum of the perovskite thin film. *F*_A_ is the fraction of absorbed light or absorptance and $h\nu$ is the photon energy. The first dotted line from the left demarcates the regions below/above 2*E*_g_. The other two dotted lines indicate the energies for 3*E*_g_ and 4*E*_g_. The arrows designate the pump energies used in TA spectroscopy measurements. The pump energies range from 1.54 eV (dark red) to 4.96 eV (purple), the same as that shown in panel (**c**). Inset is the Tauc plot indicating the bandgap. **b** TA spectra of the mixed Pb-Sn perovskite film excited with 3.06 eV (405 nm) pulses. The color indicates the amplitude Δ*A* which varies from −8 mOD (yellow) to 2 mOD (dark blue). **c** Initial amplitude |Δ*A*| for different pump energies as a function of the required *I*_A_. The dashed lines show that |Δ*A*| increases linearly with *I*_A._ The red, green and blue solid lines indicate carrier multiplication (CM) quantum yield (QY) = 1, 2 and 3, respectively. **d** CM QY variation with increasing pump energy. The blue dots represent the CM QY calculated from panel (**c**), and the error bars represent the uncertainties of the CM QY determined using the error propagation formula. The red line is a fit to the data based on a previously published model^25,28^. The fitted CM efficiency $\eta$ is 99.8%. The dashed gray line and dashed dark yellow line show the influence of uncertainty of the data on the fitted result. The dashed green line indicates the ideal case. See Supplementary Note 2 for more details. Source data are provided as a Source Data file.

TA spectroscopy is first conducted on the Cs_0.05_FA_0.5_MA_0.45_Pb_0.5_Sn_0.5_I_3_ thin films to establish their CM behavior. Unlike colloidal NCs, which can be stirred to reduce photocharging effects, measurements on thin film samples are highly susceptible to artifacts. Due care must be taken to eliminate any false signals (please refer to section “TA measurements” in Supplementary Note 1 for full details). Figure 1a shows the absorption spectra of the mixed Pb-Sn perovskite film with bandgap *E*_g_ ~ 1.24 eV (inset Tauc plot). The fraction of the absorbed light *F*_A_ is plotted as a function of energy and wavelength. The film was excited with different pump energies below and above the 2*E*_g_ threshold required for CM. Figure 1b shows a representative TA spectrum near the 1.33 eV main photobleaching (PB) peak following 3.06 eV pump excitation. The PB signal arises from state-filling at the band edge, and it tracks the time evolution of the carrier distribution. Following photoexcitation, various processes, including carrier thermalization, CM, and hot carrier cooling, occur within the first ~1 ps before the PB peak reaches its initial maximum amplitude. The initial PB signal amplitude (|Δ*A*|) at around 1–3 ps was used as a reference value, and the corresponding absorbed photon flux (*I*_A_) needed for the same initial |Δ*A*| under different pump energies was compared. (See Supplementary Note 1 and Supplementary Figs. 1 and 2 for more details about the TA measurements). With CM present, less *I*_A_ is required to attain the same initial |Δ*A*|.

The initial maximum |Δ*A*| is plotted as a function of the required *I*_A_ at pump energies ranging from 1.54 eV to 4.96 eV in Fig. 1c. A reduction of *I*_A_ for the same initial |Δ*A*| is observed at high pump photon energies larger than 2.48 eV (2*E*_g_), providing an initial indication of CM in these mixed Pb-Sn films. The gradient obtained is proportional to the CM QY. The average gradient for pump energies <2.48 eV is used as the reference value in the absence of CM effects (i.e., QY = 1). Thus, QY = 2 represents half the required *I*_A_ for the same |Δ*A*|. Above the CM threshold of 2.48 eV, for the same |Δ*A*|, the required *I*_A_ is lower, resulting in a steeper gradient (Fig. 1c). The relative CM QY is defined as the ratio of the gradient for each pump energy to the average gradient at QY = 1. Figure 1d shows the calculated CM QY as a function of pump energy normalized to the bandgap. The plot shows the onset of CM at 2*E*_g_ and a CM efficiency (${\eta }_{{{{{{\rm{CM}}}}}}}$) of ~99.4 ± 0.4%. See Supplementary Note 2 for more details about the fitting of CM efficiency^25,28^. The intrinsically low CM threshold and high CM efficiency of Cs_0.05_FA_0.5_MA_0.45_Pb_0.5_Sn_0.5_I_3_ validate its potential (see Supplementary Fig. 3 for a comparison of the theoretical CM QY and maximum PCE under AM 1.5 G). Furthermore, we have also extended the CM study to Pb-Sn mixed perovskites with different lead-tin ratios (Pb_0.75_Sn_0.25_ and Pb_0.25_Sn_0.75_) for comparison. The Pb_0.5_Sn_0.5_ shows the most efficient CM, which is possibly due to its slowest hot carrier cooling in favor of CM^23,29^. See Supplementary Figs. 4 and 5 and discussion in Supplementary Note 3 for more details. The low CM threshold of ~2*E*_g_ in the Pb-Sn mixed perovskite may be caused by asymmetric excitation, which completely transfers the excess energy to the electrons^18^. The asymmetric excitation may be due to the presence of additional sub-bands at ~2*E*_g_ (Supplementary Figs. 6–8)^18^. The sub-conduction band at ~2*E*_g_ sits at the same quasi-momentum k point as the bandgap, which fulfills both the energy conservation and momentum conservation, thus permitting asymmetric excitation and a low CM threshold of ~2*E*_g_^18^. The DFT calculation for the electronic band structure and a more detailed discussion can be found in Supplementary Note 4. We next proceed to device studies.

### CM in Pb-Sn PSC devices under monochromatic illumination

**Fig. 2:**
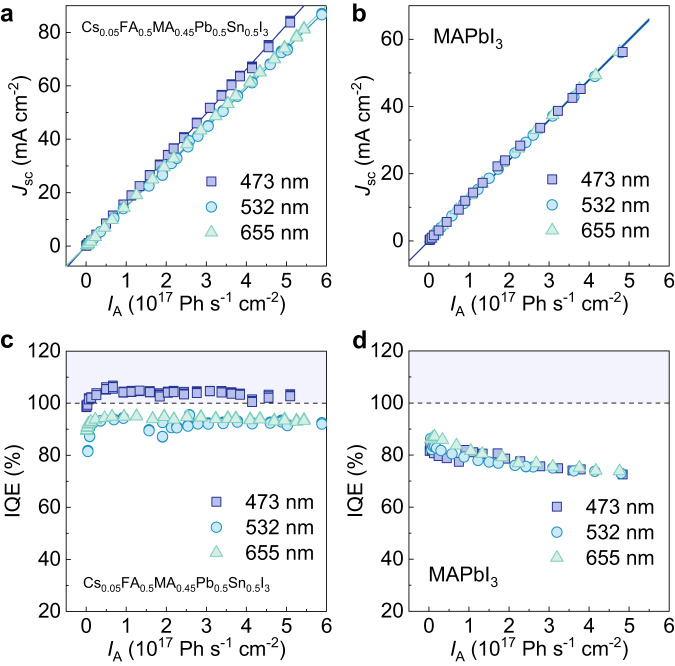
Perovskite solar cell performance under monochromatic illumination. **a**, **b** *J*_sc_ and **c**, **d** IQE as a function of absorbed photon flux (*I*_A_) under different pump energies in Cs_0.05_FA_0.5_MA_0.45_Pb_0.5_Sn_0.5_I_3_ (**a**, **c**) and MAPbI_3_ (**b**, **d**) PSCs illuminated by monochromatic CW lasers. The solid lines in (**a**) and (**b**) show that the *J*_sc_ increases linearly with *I*_A._ The horizontal dotted black lines in (**c**) and (**d**) indicate IQE = 100%. The violet-shaded regions in (**c**) and (**d**) indicate IQE > 100%. Source data are provided as a Source Data file.

While CM is evident in bare films from TA spectroscopy studies, its impact on PSC devices remains unclear. Cs_0.05_FA_0.5_MA_0.45_Pb_0.5_Sn_0.5_I_3_ and reference MAPbI_3_ PSCs with the following device structure transparent substrate/ITO/HTL/Perovskite/C_60_/BCP/Ag (Supplementary Fig. 9a, see experimental section for more information) were fabricated. To evaluate the device IQE, their short circuit current density (*J*_sc_) was first measured under monochromatic illumination with varying photon energies below/above the CM threshold of 2.48 eV (Fig. 2a, b). Then the IQE can be computed by dividing the number of electrons produced by the number of photons absorbed (Fig. 2c, d). The IQE of the perovskite layer is derived as follows (Eq. 1 and refer to Supplementary Note 5 and Supplementary Fig. 9b):

1 $${{{{{\rm{IQE}}}}}}=\frac{{J}_{{{{{{\rm{sc}}}}}}}/e}{{I}_{{{{{{\rm{A}}}}}}}}=\frac{{J}_{{{{{{\rm{sc}}}}}}}A/e}{{I}_{{{{{{\rm{A}}}}}}}A}=\frac{{J}_{{{{{{\rm{sc}}}}}}}A/e}{{{{{{{\rm{Abs}}}}}}}_{{{{{{\rm{Perovskite}}}}}}}P/h\nu }=\frac{{{{{{\rm{EQE}}}}}}}{{{{{{{\rm{Abs}}}}}}}_{{{{{{\rm{Perovskite}}}}}}}}=\frac{{{{{{\rm{EQE}}}}}}}{(1-R){T}_{{{{{{{\rm{ITO}}}}}}\_{{{{{\rm{HTL}}}}}}}}}$$

where ${J}_{{{{{{\rm{sc}}}}}}}$ is the short circuit current density, ${I}_{{{{{{\rm{A}}}}}}}$ is the absorbed photon flux, $A$ is the active area of the PSC, $e$ is the electron charge, ${{{{{{\rm{Abs}}}}}}}_{{{{{{\rm{Perovskite}}}}}}}$ is the fraction of light absorbed by the perovskite, $P$ is the power of incident light, $h\nu$ is the photon energy, *R* is the reflectance of the PSC device and *T*_ITO_HTL_ is the transmittance of ITO_HTL (Supplementary Figs. 10 and 11).

Figure 2a, b shows the *J*_sc_ as a function of *I*_A_ at various photon energies for the Cs_0.05_FA_0.5_MA_0.45_Pb_0.5_Sn_0.5_I_3_ and MAPbI_3_ reference devices. Compared to the power *P*, the absorbed photon flux *I*_A_, which eliminates the influence of excitation energies from the different CW lasers, is a more appropriate criterion for comparing the PSC photocurrent. Further discussion on its suitability and potential pitfalls can be found in Supplementary Note 6 (Supplementary Figs. 12–15). The *J*_sc_ and gradient for the mixed Pb-Sn PSC increased for the 473 nm (2.62 eV, 2.11*E*_g_) excitation source (Fig. 2a), indicating the likely presence of CM. The average gradient of the plots for the 532 nm and 655 nm excitation (i.e., 1.88 *E*_g_ and 1.52 *E*_g_, respectively, and below the CM threshold) is used as a reference value and set to unity. Hence, the gradient for 473 nm excitation (above the CM threshold) is approximately 1.10 times that of the reference. A similar rise in *J*_sc_ is also observed for (FASnI_3_)_0.6_(MAPbI_3_)_0.4_ PSCs (Supplementary Figs. 16 and 17). In contrast, the MAPbI_3_ reference device shows a constant gradient for all the different excitation sources (Fig. 2b). In the absence of CM, the short circuit current density *J*_sc_ of the PSC device should remain constant for the same photon flux *I*_A_ regardless of excitation energies. MAPbI_3_ possesses a bandgap of 1.55 eV (800 nm) and requires a CM threshold of ≥3.1 eV (≤400 nm). Hence, no CM effect is present at 473 nm (2.62 eV), 532 nm (2.33 eV) and 655 nm (1.89 eV) as expected.

Figure 2c, d shows the corresponding IQE of the Cs_0.05_FA_0.5_MA_0.45_Pb_0.5_Sn_0.5_I_3_ and MAPbI_3_ PSCs. With CM present, the IQE obtained with 473 nm excitation is greater than that at 532 nm and 655 nm excitation (Fig. 2c) for the mixed Pb-Sn sample, and it exceeds 100%. This increment is absent in the MAPbI_3_ reference sample since it is excited below its CM threshold (Fig. 2d). Other metrics such as open-circuit voltage (*V*_oc_), fill factor (FF) and PCE can be found in Supplementary Figs. 18–21. The PCE produced under monochromatic illumination with different photon energies is normalized to *I*_A_ for a fair comparison (Supplementary Fig. 22). Above the CM threshold, PCE enhancement is observed for the mixed Pb-Sn PSC devices but is absent in the MAPbI_3_ reference PSC devices. More discussion can be found in Supplementary Notes 7–9 and Supplementary Figs. 22 and 23. All this evidence supports the presence of the CM effect in the mixed Pb-Sn PSC.

### CM in Pb-Sn PSC devices under broadband illumination

Next is the performance of the mixed Pb-Sn PSCs under broad illumination. We have also established that CM effects are present in mixed Pb-Sn PSCs with typical glass substrates and architectures used in PSC research but are overshadowed by parasitic absorption loss from glass and ITO and recombination loss during carrier extraction in the high-energy region. A detailed discussion can be found in Supplementary Note 10 and Supplementary Figs. 24 and 25. To maximize any gains from CM in PSC devices, the optimized PSC device structure of Quartz/ITO/self-assembled monolayer (SAM) [2-(9H-carbazol-9-yl)ethyl]phosphonic acid (2PACz)/Perovskite/C_60_/BCP/Ag is utilized^30–32^. The quartz substrate has a greater transmittance in the UV region, and the SAM 2PACz exhibits a better energy level alignment with the Pb-Sn mixed perovskite (Supplementary Figs. 26 and 28), which can minimize the parasitic losses and reduce carrier recombination losses in the PSCs.

**Fig. 3:**
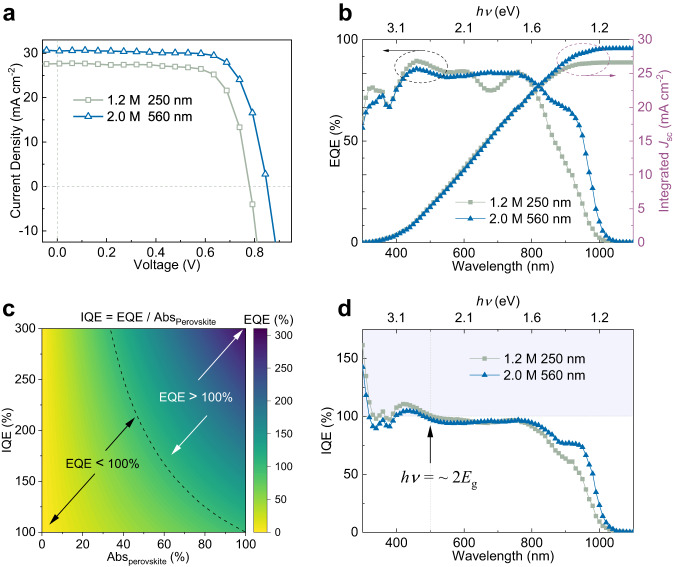
PSC performance under broadband solar illumination. **a** *J*-*V* curves of Cs_0.05_FA_0.5_MA_0.45_Pb_0.5_Sn_0.5_I_3_ PSC devices with two perovskite layer thicknesses (i.e., the thinnest and thickest layers) measured under one sun. The dashed lines indicate *J* = 0 mA cm^−2^ (horizontal) and *V* = 0 V (vertical). **b** The corresponding EQE (black *Y*-axis on the left, indicated by the black dotted circle) and the integrated *J*_sc_ (red *Y*-axis on the right, indicated by the red dotted circle) of PSC devices with different perovskite layer thicknesses. **c** Relationship between the EQE, IQE and perovskite absorptance Abs_perovskite_. The dotted line is the EQE = 100% boundary. On the left (from yellow to green), the EQE is smaller than 100%, although IQE is larger than 100%. The low EQE is due to decreased Abs_perovskite_. On the right (from green to blue), both the EQE and IQE are larger than 100%. **d** IQE of the PSCs. The dashed lines indicate IQE = 100% (horizontal) and wavelength = 500 nm (vertical). The arrow points to the CM threshold at around 2*E*_g_. The violet-shaded region in (**d**) indicates IQE > 100%. Source data are provided as a Source Data file.

Figure 3a shows the *J*-*V* curves of the champion devices obtained under one Sun (AM 1.5 G) for absorber thickness of 250 nm and 560 nm (i.e., the thinnest and the thickest) obtained by varying precursor concentrations from 1.2 M to 2.0 M. *J-V* curves of the champion devices for other perovskite thicknesses can be found in Supplementary Fig. 29. Their parameters are tabulated in Table 1. Details of their thicknesses and morphology can be found in Supplementary Figs. 30–33. Figure 3a shows that the device fabricated with 2.0 M precursor concentration (560 nm thick layer) yields the highest PCE of 19.19%, with *V*_oc_ of 0.85 V, *J*_sc_ of 30.55 mA cm^−2^, and FF of 0.74. The *J*_sc_ values are also corroborated by the integrated *J*_sc_ from the EQE spectrum with a relative discrepancy of ~6% (Supplementary Table 1). The reproducibility and statistics for the mixed Pb-Sn device characterization can be found in Supplementary Fig. 34 and Supplementary Table 2. In general, the average values for the PSCs in Supplementary Fig. 34 and Supplementary Table 2 show the same trend as the champion devices in Table 1.

**Table 1:** The optoelectronic performance of the champion Cs_0.05_FA_0.5_MA_0.45_Pb_0.5_Sn_0.5_I_3_ PSCs with different absorber layer thicknesses

Concentration	Thickness (nm)	*V*_oc_ (V)	*J*_sc_ (mA cm^−2^)	FF	PCE (%)
1.2 M	250	0.78	27.75	0.74	16.06
1.4 M	330	0.86	28.57	0.73	17.97
1.6 M	400	0.86	29.16	0.73	18.35
1.8 M	480	0.85	30.08	0.73	18.85
2.0 M	560	0.85	30.55	0.74	19.19

Figure 3b shows the EQE and integrated *J*_sc_ for devices with different active layer thicknesses. An EQE value of less than 100% does not exclude the presence of CM in PSCs. This is because EQE represents the proportion of incoming photons converted to the external circuit electrons, regardless of whether the photons are absorbed by the perovskite active layer. On the other hand, IQE represents the ratio of the population of external circuit charge carriers produced by the solar cell to the corresponding number of photons absorbed by the active layer of the device^33^. Hence IQE is the key parameter for studying CM^33^. An IQE exceeding 100% indicates that by absorbing one photon, more than one electron is generated and transported to the external circuit, validating the existence of CM in the devices.

Figure 3c shows the theoretical relationship between EQE, IQE and the proportion of light absorbed by the perovskite layer in the device (Abs_perovskite_). If Abs_perovskite_ is small (e.g., 40%), even though 2 electrons are produced from 1 absorbed photon due to CM (i.e., IQE = 200%), the resultant EQE is still only 80% (EQE = IQE × Abs_perovskite_ = 200% × 40% = 80%). Conversely, for large IQE (=300%), the extra electrons could compensate for the small Abs_perovskite_, then the EQE value could exceed 100% (EQE = 300% × 40% = 120%). Hence, IQE exceeding 100% is the key evidence for evaluating the presence of CM in PSC devices.

**Fig. 4:**
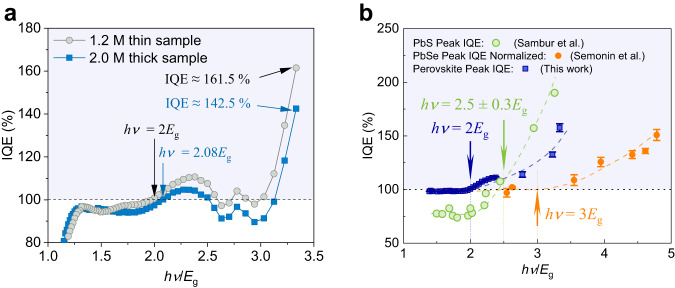
IQE exceeds 100% in PSCs. **a** IQE as a function of $h\nu /{E}_{{{{{{\rm{g}}}}}}}$ variation in PSCs with 1.2 M thin perovskite layer and 2.0 M thick perovskite layer. The arrows show that the highest IQE of ~161.5% and ~142.5% are obtained in the 1.2 M thin sample and 2.0 M thick samples at 3.33*E*_g_ with CM threshold of 2*E*_g_ and 2.08*E*_g_, respectively. **b** Comparison of peak IQE value of Pb-Sn mixed PSCs, PbSe solar cells and PbS photovoltaic devices. Blue squares are the average peak IQE of the glass and quartz-based Pb-Sn mixed PSCs with both thick and thin perovskite layers, and the error bars are the standard deviation. Orange circles are the normalized IQE value of PbSe solar cells taken from reference^34^. Green open circles are the peak IQE value of PbS devices taken from reference^35^. CM threshold for PSCs is ~2*E*_g_ which is smaller than the 3*E*_g_ of PbSe solar cells and 2.5*E*_g_ of PbS devices. The horizontal dashed black lines in (**a**) and (**b**) indicate IQE = 100%. The vertical dashed lines and arrows in (**b**) indicate the CM thresholds of the PSCs (blue), PbS devices (green) and PbSe solar cells (orange). The dashed curves indicate the variation trend of the IQE values. The violet-shaded regions in (**a**) and (**b**) indicate IQE > 100%. Source data are provided as a Source Data file.

Figure 3d shows the mixed Pb-Sn PSC devices with IQE > 100% and CM threshold of ~2*E*_g_ (500 nm). The IQE is calculated from Fig. 3b using Eq. 1, where we considered the reflection of photons by the device (*R*) and the transmittance (*T*) of the ITO and 2PACz layers. Details on the *R, T* and IQE reproducibility are given in Supplementary Fig. 35. The plots of IQE *vs* $h\nu /{E}_{{{{{{\rm{g}}}}}}}$ (Fig. 4a) clearly shows the CM threshold of ~2*E*_g_ and maximum IQE values of 150 ± 10% for the PSC devices at 3.33*E*_g_. Figure 4a shows that in the thin perovskite layer PSC (1.2 M), the CM threshold is ~2*E*_g_ and the highest IQE can reach ~161.5% at 3.33*E*_g_, while the CM threshold of the thick perovskite layer PSC (2.0 M) is around 2.08*E*_g_, and the highest IQE is around 142.5%. The difference between their IQEs is possibly due to carrier recombination loss in the thicker perovskite layer. For high-energy photons with shorter penetration depth, the photons are fully absorbed in the first hundreds of nanometers. In the thin perovskite layer PSC, the generated electrons can be efficiently extracted to the electron transport layer located deeper from the incident light direction. In the thick perovskite layer PSC, the generated electrons may suffer from increased intrinsic recombination loss during transport, leading to a reduced IQE. Nonetheless, the low-energy photons, which have longer penetration depth, can still be efficiently extracted in the thick perovskite film. This is consistent with the study on glass-based PSCs. More discussion can be found in Supplementary Note 11 and Supplementary Figs. 36–40. Optical modeling of the 1.2 M quartz-ITO PSC further validates that the IQE exceeds 100% (Supplementary Fig. 41). See Supplementary Note 12 for more information about optical modeling. The IQE exceeding 100% in the Pb-Sn mixed PSCs is further confirmed through crosschecking with the setup at the Solar Energy Research Institute of Singapore (SERIS) (Supplementary Fig. 42). Additional details regarding the perovskite layer and the PSCs, including information about hysteresis and stability, are available in the Supplementary Figs. 43–45.

Lastly, we compare the peak IQEs of our mixed Pb-Sn PSC devices with that of conventional PbSe solar cells and PbS photovoltaic devices reported in the literature (Fig. 4b)^34,35^. In contrast to PbSe solar cells with CM threshold of 3*E*_g_ and PbS photovoltaic devices with CM threshold of 2.5±0.3 *E*_g_, our mixed Pb-Sn PSCs have a smaller CM threshold of 2*E*_g_. Our PSCs can achieve an IQE of ~160% at *hv* = 3.33*E*_g_, while the PbSe solar cells require at least 4.78*E*_g_ to reach an IQE of ~150%, and the PbS photovoltaic devices need *hv* = 3.26*E*_g_ for an IQE of ~190%. Our mixed Pb-Sn PSCs compare favorably and present an alternative approach to maximize PCE enhancements from CM effects.

## Discussion

Our findings of IQE > 100% provide clear evidence that CM may already be present in the mixed Pb-Sn PSCs. Nonetheless, it is evident that the typical PSC architecture is the primary limiting factor due to: (1) competing requirements of thin absorber layers to maximize the CM effect from high-energy (short wavelength) photons while requiring adequate thickness to effectively harvest the low-energy photons (Supplementary Figs. 24 and 25); (2) carrier losses due to defects (possibly from Sn^2+^ oxidation) which tend to be more severe in the thicker layers (Supplementary Figs. 24 and 25); (3) parasitic absorption losses from the glass substrate (Supplementary Fig. 26) and the intervening layers (e.g., ITO—Supplementary Figs. 35b and 36b); and (4) energy level alignment and carrier recombination at the perovskite/HTL interface impacting charge transport and extraction (Supplementary Fig. 28). Parasitic absorption losses from the ITO-substrate over the regions of 2.5–3.0*E*_g_ is a major contribution to stymieing the effects of CM. Replacement of the HTL and the quartz substrate mitigated some of these losses for the contributions from the CM effects to be revealed. Interestingly, such CM effects may already be present in mixed Pb-Sn PSCs but may be obscured by other contributions to the IQE/EQE. To fully leverage the CM effect in these mixed Pb-Sn PSCS, several strategies can be considered in future works, such as using new transparent conducting layers (e.g., graphene) with high UV transparency to replace ITO; engineering new perovskite absorbers with higher optical thickness at long wavelengths; interface engineering to further suppress the defects and recombination losses; as well as developing new ETL materials to enhance the carrier transport and extraction, etc. Another exciting possibility is the perovskite tandem configuration where thin mixed Pb-Sn PSCs exploiting the CM effects at the short wavelengths could be combined with Si solar cells or conventional perovskite solar cells to maximize the spectral coverage at the longer wavelengths.

In summary, we uncover clear evidence of CM in mixed Pb-Sn perovskite solar cells with an IQE exceeding 110% without the application of external gain or any bias voltage. Transient spectroscopy validates the high CM efficiency (99.4 ± 0.4%) and the low CM threshold of 2*E*_g_ present in these Cs_0.05_FA_0.5_MA_0.45_Pb_0.5_Sn_0.5_I_3_ bare films. Monochromatic and broadband illumination studies on PSC devices further reveal the intricate interplay of various factors ranging from absorber thickness, recombination losses, parasitic absorption losses, and interfacial energy alignments that impede potential PCE gains from CM effects. By replacing the commonly used PEDOT:PSS HTL and glass-ITO substrates with 2PACz and quartz-ITO substrates, we demonstrate that these limitations could be overcome to attain an IQE as large as >160% in a PSC solar cell. Importantly, our findings reveal the presence of CM effects in mixed Pb-Sn perovskites contributing to the device performance but are overshadowed by other contributions. A comprehensive redesign of PSC device architecture, together with new optimized charge transport layers, is needed to fully exploit CM effects in next-generation PSCs or tandem PSCs to break the SQ limit.

## Methods

### Materials

Patterned glass-based ITO was purchased from Shenzhen Huayu Union Technology Co., Ltd. Poly(3,4-ethylenedioxythiophene)-poly(styrenesulfonate) (PEDOT: PSS) was purchased from Heraeus Precious Metals GmbH. Tin iodide (SnI_2_), Cesium iodide (CsI), bathocuproine (BCP), tin powder (Sn), *N, N*-dimethylformamide (DMF), dimethyl sulfoxide (DMSO), toluene, tin (II) fluoride (SnF_2_), guanidine thiocyanate (GuaSCN), and ethylenediamine (EDA) were purchased from Sigma-Aldrich. Methylammonium iodide (MAI) and formamidinium iodide (FAI) were purchased from Greatcell Solar Materials. Lead iodide (PbI_2_) and [2-(9H-carbazol-9-yl)ethyl]phosphonic acid (2PACz) were purchased from Tokyo Chemical Industry Co. Ltd (TCI). C_60_ was purchased from Puyang Yongxin Fullerene Technology Co., Ltd. Quartz-based ITO was purchased from Luoyang Guluo Glass Co. Ltd. All materials and solvents were used as received without any further purification.

### Film preparation

Cs_0.05_FA_0.5_MA_0.45_Pb_0.5_Sn_0.5_I_3_ precursor solution was prepared by mixing MAI (0.45 mmol), FAI (0.5 mmol), CsI (0.05 mmol), PbI_2_ (0.5 mmol), SnI_2_ (0.5 mmol), SnF_2_ (0.05 mmol), GuaSCN (0.035 mmol) and Sn powder (0.1 g) into mixed solvent of DMF and DMSO (volume ratio 4:1). The narrow bandgap (FASnI_3_)_0.6_(MAPbI_3_)_0.4_ precursor solution was prepared by mixing MAI (0.4 mmol), PbI_2_ (0.4 mmol), FAI (0.6 mmol), SnI_2_ (0.6 mmol), SnF_2_ (0.06 mmol), GuaSCN (0.042 mmol) and Sn powder (0.1 g) into mixed solvent of DMF and DMSO (volume ratio 4:1). The thickness of perovskite films was tuned by the concentration of perovskite precursor. Thin films (30 nm) for characterization and thick films (200 nm, 260 nm, 340 nm, 420 nm, 500 nm) for devices were prepared with 0.2 M and 1.2–2.0 M (1.2 M, 1.4 M, 1.6 M, 1.8 M, 2.0 M) precursor by spin-coating at 5000 rpm for 30 s, using 180 μL of toluene as anti-solvent during the spinning process and annealing at 100 °C for 10 min. For Cs_0.05_FA_0.5_MA_0.45_Pb_0.5_Sn_0.5_I_3_ thin films prepared with vacuum-assisted crystallization (vacuum pumped at ~−1 bar for 8 min before annealing), the thicknesses for 1.2–2.0 M precursor are 250 nm, 330 nm, 400 nm, 480 nm and 560 nm (i.e., spin-coated at 5000 rpm for 30 s).

MAPbI_3_ precursor solution was prepared by mixing 1.55 M stoichiometric MAI and PbI_2_ into a mixed solvent of DMF and DMSO (volume ratio 9:1). The precursor solution was spin-coated at 1000 rpm for 10 s and 6000 rpm for 30 s. 180 μL of toluene was dropped onto the precursor as an anti-solvent during the second spinning process. The fabricated film was annealed at a 100 °C hotplate for 60 min. The thickness of the MAPbI_3_ perovskite layer is around 340 nm. See Supplementary Fig. 32f.

### Device fabrication

The Pb-Sn mixed PSCs discussed in the main manuscript are based on the structure of Quartz/ITO/2PACz/Perovskite/C_60_/BCP/Ag. The preparation process of inverted structure devices (ITO/2PACz/Perovskite/C_60_/BCP/Ag) was as follows: Patterned ITO glass or quartz was ultrasonically cleaned with detergent, acetone, ethanol, and distilled water in sequence, followed by a 10-min plasma cleaning. The 2PACz (0.5 mg mL^−1^ in ethanol) self-assembled monolayer (SAM) was spin-coated on the ITO substrates at a speed of 5000 rpm for 30 s and followed by annealing at 100 °C for 10 min. Then, ethanol was applied to wash the unbounded 2PACz at a speed of 5000 rpm for 30 s, followed by annealing at 100 °C for 5 min^36^. The perovskite films were spin-coated as mentioned above and followed by vacuum pumping at ~−1 bar for 8 min^37^. Subsequently, the perovskite films were annealed at 100 °C for 10 min. EDA solution (0.1 m M in toluene) was spin-coated at 5000 rpm for 50 s onto the perovskite film and followed by annealing at 70 °C for 5 min as a post-treatment for Pb-Sn PSCs. Eventually, C_60_ (50 nm)/BCP (6 nm)/Ag (100 nm) were sequentially deposited on the perovskite layer via thermal evaporation. For PCE measurement under 1 Sun (AM 1.5 G) condition and EQE measurement, a 100 nm LiF anti-reflection (AR) layer was deposited on the ITO glass/quartz (glass/quartz side).

The preparation process of inverted structure devices (ITO/PEDOT:PSS/Perovskite/C_60_/ BCP/Ag) was as follows: Patterned ITO glass was ultrasonically cleaned with detergent, acetone, ethanol, and distilled water in sequence, followed by a 10-min plasma cleaning. Filtered PEDOT:PSS was spin-coated on the ITO substrates at a speed of 900 rpm for 10 s and 4000 rpm for 60 s and followed by annealing at 150 °C for 10 min. After the substrates cooled down, the perovskite layer was prepared on the PEDOT:PSS substrate, as mentioned above. Subsequently, the perovskite films were annealed at 100 °C for 10 min to remove the solvent and stimulate crystallization. As a post-treatment for Pb-Sn PSCs, EDA solution (0.1 m M in toluene) was spin-coated at 5000 rpm for 50 s onto the perovskite film and followed by annealing at 70 °C for 5 min. The preparation of the perovskite layer was done in a nitrogen-filled glovebox. Eventually, C_60_ (50 nm)/BCP (6 nm)/Ag (100 nm) were sequentially deposited on the perovskite layer via thermal evaporation. For PCE measurement under 1 Sun (AM 1.5 G) condition and EQE measurement, a 100 nm LiF anti-reflection (AR) layer was deposited on the ITO glass (glass side).

For CM study of devices under monochromatic illumination presented in the main manuscript, the thickness of the Pb-Sn mixed perovskite (1.6 M 5k rpm) is ~400 nm, and the pure Pb-based perovskite (1.55 M 6k rpm) is around 340 nm, as shown in Supplementary Figs. 30c and 32f. For CM study of devices under monochromatic illumination presented in Supplementary Notes 7 and 8 and Supplementary Figs. 15, 19 and 22, the thickness of the Pb-Sn mixed perovskite prepared without the vacuum-assisted crystallization method (1.6 M 5k rpm) is around 340 nm (Supplementary Fig. 32c), which is comparable with the Pb counterpart.

### Characterization

XRD patterns (Supplementary Fig. 43) were collected with a Rigaku SmartLab High-resolution X-ray diffractometer^38^. Diffraction signal 2*θ* was collected from 3° to 60° with a step size of 0.02°. Absorption and reflection spectra were measured using a Shimadzu UV-3600 plus UV-VIS-NIR spectrometer equipped with an integrating sphere in the range from 300 to 1250 nm. The current density-voltage (*J-V*) curves of PSC devices under one sun were recorded by a Keithley 2420 source meter under a Sciencetech UHE-NSC Class AAA solar simulator. The AM 1.5 G one sun intensity (100 mW cm^−2^) was calibrated using an Oriel PV Reference Cell System (91150 V)^39^. The active area of the solar cells is 0.06 cm^2^. The measurements were conducted in a N_2_-filled glovebox (O_2_ < 10 ppm, H_2_O < 1.0 ppm) at room temperature. The current *J-V* curves and parameters of PSC devices under monochromatic illuminations were measured with continuous wave (CW) lasers (Changchun New Industries Optoelectronics Tech. Co., Ltd.). The area of solar cell is 0.09 cm^2^. The active area of the solar cell is determined by the beam size of the laser beam, which is smaller than the area of the solar cell. For the 2PACz-based Cs_0.05_FA_0.5_MA_0.45_Pb_0.5_Sn_0.5_I_3_ PSCs, the active areas are 0.00475 cm^2^ (473 nm), 0.00202 cm^2^ (532 nm) and 0.00196 cm^2^ (655 nm). For the PEDOT: PSS-based Cs_0.05_FA_0.5_MA_0.45_Pb_0.5_Sn_0.5_I_3_ PSCs, the active areas are 0.02118 cm^2^ (473 nm), 0.00558 cm^2^ (532 nm) and 0.00369 cm^2^ (655 nm). For the MAPbI_3_ PSCs, the active areas are 0.01102 cm^2^ (473 nm), 0.00719 cm^2^ (532 nm) and 0.00693 cm^2^ (655 nm). For the (FASnI_3_)_0.6_(MAPbI_3_)_0.4_ PSCs, the active areas are 0.01321 cm^2^ (473 nm) and 0.00362 cm^2^ (785 nm). The beam size is measured with a CMOS camera (Thorlabs, DCC1545M-GL), and the diameter is defined as 1 e^−2^ of the maximum intensity, assuming a Gaussian beam profile. The measurements were conducted in air at room temperature, and the solar cells were encapsulated in the N_2_-filled glovebox in advance. The *J* − *V* curves were measured in reverse scan from 1.0 V to −0.2 V at a scan rate of ~0.5 V s^−1^. EQE for PSC devices were recorded using a Zolix Solar Cell Scan100 measurement system (Zolix Instruments Co., Ltd.). Material constants (refractive index *n* and extinction coefficient *k*) were modeled using a Kramers-Kronig-consistent b-spline model based on the ellipsometry data obtained with an α-SE^TM^ spectroscopic Ellipsometer (J. A. Woollam Co., Inc.). UPS measurements were conducted using an XPS Shimadzu Kratos Axis Supra with a He I (21.2 eV) lamp. The material’s thickness and morphology were measured with a Bruker BioScope Resolve Atomic Force Microscope (AFM). Transient Absorption (TA) spectroscopy was performed using the HELIOS^TM^ femtosecond transient absorption spectrometer (HELIOS^TM^, Ultrafast Systems, LLC). The pump pulses were directed from the regeneration amplifier (Coherent Libra™, 800 nm, 1 kHz, 50 fs) or were generated from a BBO crystal via second harmonic generation (SHG) of the 800 nm fundamental (400 nm), or were generated with an optical parametric amplifier (OPA) system (Coherent OPeRA Solo™). The probe was a NIR white-light continuum (800–1400 nm) generated by focusing part of the 800 nm fundamental beam into a sapphire crystal.

### Reporting summary

Further information on research design is available in the Nature Portfolio Reporting Summary linked to this article.

### Supplementary information

Supplementary Information

Peer Review File

Reporting Summary

### Source data

Source Data

## Data Availability

All data are available in the main text and the Supplementary Information. The data that support the findings of this study are also openly available in DR-NTU (Data) at 10.21979/N9/ZEZWAM. Source data are provided with this paper.
