# Supplementary material for: Carrier multiplication in perovskite solar cells with internal quantum efficiency exceeding 100%
Source: Nat Commun. 2023 Oct 9;14:6293. doi: 10.1038/s41467-023-41758-w (PMC10562407; doi:10.1038/s41467-023-41758-w)
Supplement: Supplementary file 1 — Supplementary Information [file 41467_2023_41758_MOESM1_ESM.pdf]

Supplementary Information

Carrier Multiplication in Perovskite Solar Cells with Internal Quantum Efficiency exceeding 100%

Yue Wang[1], Senyun Ye[1], Jia Wei Melvin Lim[1], David Giovanni[1], Minjun Feng[1], Jianhui Fu[1], Harish Natarajan Swaha Krishnamoorthy[2,3], Qiannan Zhang[1], Qiang Xu[1], Rui Cai[1], Tze Chien Sum[*,1]

[1]Division of Physics and Applied Physics, School of Physical and Mathematical Sciences, Nanyang Technological University, 21 Nanyang Link, Singapore 637371, Singapore.

[2] Centre for Disruptive Photonic Technologies, TPI, School of Physical and Mathematical Sciences, Nanyang Technological University, Singapore 637371, Singapore.

[3]Present address: Tata Institute of Fundamental Research-Hyderabad, Sy. No. 36/P, Gopanapally Village, Serilingampally Mandal, Hyderabad-500046, India

Corresponding Author

*Tze Chien Sum. Email: Tzechien@ntu.edu.sg

This PDF file includes:

Supplementary Notes
Supplementary Figures.
Supplementary Tables.
Supplementary References

Supplementary Notes

1. Transient Absorption (TA) measurements

CM/MEG is typically assessed using the TA method developed by Schaller and Klimov.[1] Evidence of CM/MEG is based on the occurrence of a fast decay component at the early times in the photobleaching (PB) peak under low pump fluence. The CM/MEG Quantum Yield (QY) is then calculated by taking the ratio between the early time PB amplitude (A, initial exciton population) and quasi-equilibrium amplitude (B, long-lived single exciton population) (i.e., $R_{\mathrm{pop}} = \frac{\mathrm{A}}{\mathrm{B}} = \frac{\delta\langle N_0\rangle \cdot QY}{1-\exp(-\langle N_0\rangle)}$ where $\langle N_0\rangle$ is the average number of absorbed photons per nanocrystal (NC) and δ stands for single exciton decay), as shown in Supplementary Fig. 1a.[1-4] However, A/B is very sensitive to the condition of the sample's surface. For instance, surface charge trapping caused by photo-charging could induce additional time constants leading to a lower B value (Supplementary Fig. 1b)).[5] The fast depopulation of PB and inflated A/B ratio could lead to false CM/MEG signals. Colloidal quantum dot (QD) samples are typically stirred in a cuvette to avoid potential photo-charging effects.[2] However, such photocharging effects are inevitable in thin films when the beam is fixed at a single measurement point. Moving the sample stage (analogous to stirring for colloidal QD samples) could help reduce the photocharging effects, but not eliminate them completely.

Thus, we directly compare the efficiency of carrier photogeneration by monitoring the required amount of absorbed photon flux to attain a reference photobleaching intensity amplitude for different pump photon energies. The initial value of the band edge photobleaching peak from TA (ΔA_{max}) was used to characterize the initial carrier photogeneration. With CM/MEG present, less

photon absorption (i.e., absorbed photon flux (I_A)) will be required to attain the same bleach intensity (or the magnitude of the change in absorbance, ΔA_{max}, Supplementary Fig. 1c) that translates to a higher photogeneration efficiency. This will help mitigate the influence of false CM/MEG signals arising from a decreasing magnitude of B.

To illustrate the influence of photocharging on CM assessment, Supplementary Fig. 1d shows the normalized TA dynamics of a $(FASnI_3)_{0.6}(MAbI_3)_{0.4}$ thin film pumped with a 2.38 eV (522 nm) pulse (below CM threshold) with the sample fixed or moving. Severe photo-charging ensues as expected with the sample fixed, yielding a much shorter lifetime of 3.2 ± 0.4 ns. Conversely, a longer lifetime of 14 ± 5 ns is obtained for the moving sample, indicating less photocharging. The reduced lifetime in the former leads to an enlarged A/B ratio (i.e., $\frac{A'}{B'} > \frac{A}{B}$ - Supplementary Fig. 1b) In the absence of CM, the A/B ratio should approach 1 with decreasing pump intensity as there is no extra carriers generated that will contribute to the initial PB amplitude.[6] This additional fast decay component due to surface traps is present even under low pump power (see power-dependent studies in Supplementary Fig. 2). Supplementary Fig. 1e shows the variation of A/B with the initial maximum amplitude $|\Delta A|$ (where $|\Delta A|$ is proportional to the pump intensity). Indeed, the moving sample exhibits a ratio of A/B approaching 1 at low pump intensity. Instead, with photocharging, the stationary sample shows a spurious signal as large as A/B = 3 at the same low pump intensity (at below CM threshold). The A/B ratio is even more pronounced at higher pump intensities, which could lead to false deductions of CM.

It is important to note that the required photon flux I_A to attain the same change in absorbance ($|\Delta A_{max}|$) for the two conditions (fixed vs moving sample) remains the same since carrier recombination is minimal in the first few picoseconds. Supplementary Fig. 1f shows that I_A linearly

increases with $|\Delta A_{\max}|$ and the gradients under the two conditions are similar. This indicates that the photo-charging induced surface traps have no significant effect on the initial PB signal amplitude. By considering the required photon flux for generating a certain initial carrier population, we could eliminate the influence from the longer-lived B signal, thus circumventing the photo-charging issue.

2. CM/MEG QY Fitting Model

The previously reported cascade model was adopted to fit the QY data determined from TA measurement.[7] According to this model, the QY can be represented by:

$$\mathrm{QY} = \sum_{j=1}^{m} \frac{k_{\mathrm{cool}} \prod_{i=1}^{j} k_{\mathrm{MEG}}^{(i=1)}}{\prod_{i=1}^{j} (k_{\mathrm{cool}} + k_{\mathrm{MEG}}^{(i)})} \quad \text{(S1)}$$

where k_{MEG} is the multi-exciton generation (MEG) or carrier multiplication (CM) rate and k_{cool} is the carrier cooling rate. The relationship between the two can be given by:[8]

$$k_{\mathrm{MEG}} = k_{\mathrm{cool}} P \left(\frac{h\nu - h\nu_{\mathrm{th}}}{h\nu_{\mathrm{th}}} \right)^{s} \quad \text{(S2)}$$

where $h\nu_{th}$ is the CM threshold energy, P is a constant relating to the CM efficiency, and s is an exponent that varies between 2 to 5. The CM efficiency η is then given by:

$$\eta = \frac{P}{1 + P} \quad \text{(S3)}$$

The data in Figure 1d of the main text can be well-fitted with this model and the fitted parameters were P = 500 and s = 2.5 ± 0.2. The value of P obtained leads to $\eta \sim$ 99.8 %, as described in the main text.

3. Carrier Multiplication in Pb-Sn mixed perovskite with different lead-tin ratio

We have also probed the CM effects in other Pb-Sn mixed perovskite compositions – $Cs_{0.05}FA_{0.5}MA_{0.45}Pb_{0.75}Sn_{0.25}I_3$ (termed $Pb_{0.75}Sn_{0.25}$ thereafter) and $Cs_{0.05}FA_{0.5}MA_{0.45}Pb_{0.25}Sn_{0.75}I_3$ (termed $Pb_{0.25}Sn_{0.75}$ thereafter) with the same method. Supplementary Fig. 4 shows the steady state absorption and transient absorption spectra of the samples. The $Pb_{0.75}Sn_{0.25}$ sample has a larger bandgap of ~1.28 eV while that for $Pb_{0.25}Sn_{0.75}$ is around 1.24 eV. The CM QY as a function of bandgap multiples for the two samples and the fitted CM efficiencies are depicted in Supplementary Fig. 5. The blue dots are the CM QY at each $h\nu/E_g$, the red dotted lines show the fitted CM efficiencies (93.3% for the $Pb_{0.75}Sn_{0.25}$ and 95.2% for the $Pb_{0.25}Sn_{0.75}$), and the grey/dark yellow dashed line show the lowest/highest CM efficiency considering the uncertainties (85.7%, 96.2% for the $Pb_{0.75}Sn_{0.25}$ and 92.3%, 98.0% for the $Pb_{0.25}Sn_{0.75}$). Considering the uncertainty, the CM efficiency of the $Pb_{0.75}Sn_{0.25}$ sample is around 91 ± 5 % and that of the $Pb_{0.25}Sn_{0.75}$ sample is around 95 ± 3 %. Both samples show a lower CM efficiency and slightly larger CM threshold. The CM threshold is calculated according to the equation:[7]

$$h\nu_{\mathrm{th}} = E_{\mathrm{g}} + E_{\mathrm{g}}/\eta \qquad \text{(S4)}$$

where $h\nu_{\mathrm{th}}$ is the CM threshold, E_{g} is the bandgap, and η is the CM efficiency. The $h\nu_{\mathrm{th}}$ of the $Pb_{0.75}Sn_{0.25}$ sample is ~ 2.10E_{g} and the $h\nu_{\mathrm{th}}$ of the $Pb_{0.25}Sn_{0.75}$ is ~ 2.05E_{g}. The lower CM

efficiency and larger CM threshold is probably due to a faster hot carrier cooling than the $Pb_{0.5}Sn_{0.5}$ sample.[9] In the previous study on the hot carrier cooling in Pb-Sn mixed perovskite, the slowest hot carrier cooling is obtained in a perovskite with a 40% Pb, 60% Sn composition. The 80% Pb, 20% Sn sample and 20% Pb, 80% Sn both show much faster hot carrier cooling.[9] That is possibly the reason why the 50% Pb, 50% Sn sample shows the most efficient CM, while the 75% Pb or 75% Sn samples show a lower CM efficiency and a larger CM threshold. Hence, the CM QY of the latter increases more gradually than the $Pb_{0.5}Sn_{0.5}$ sample.

4. DFT calculation and the explanation for the low CM threshold in the Pb-Sn mixed perovskite

The mechanisms of CM/MEG are still under debate and the exact mechanism of CM/MEG in perovskite is still unclear. One possible explanation for the low CM threshold in perovskite materials is the asymmetric excitation which completely transfers the excess energy to the electrons or holes.[10] Apart from dissimilar electron and hole effective masses, higher valence and conduction band in confined and bulk materials can also result in asymmetric excitation and determine the onset of the CM.[10,11] An additional band at around $2E_g$ which may lead to asymmetric excitation could also be one of the possible reasons of the low CM threshold in the Pb-Sn mixed perovskite.[10]

We construct a supercell of $FA_{0.5}MA_{0.5}Pb_{0.5}Sn_{0.5}I_3$ perovskite in the cubic phase. For simplicity and to lower the computational cost, here we have used $FA_{0.5}MA_{0.5}Pb_{0.5}Sn_{0.5}I_3$ instead of $Cs_{0.05}FA_{0.5}MA_{0.45}Pb_{0.5}Sn_{0.5}I_3$. This is because, the top of valence band (VB) and the bottom of the conduction band (CB) are mainly formed by Pb 6P, Sn 4P, I 5P and the electronic states associated with Cs, FA, MA are located away from the band edges. The electronic band structure is obtained

from density functional theory (DFT) calculations with spin-orbit coupling (SOC) effect considered. We utilized the all-electron-like projector augmented wave (PAW) method[12] and the PW86R exchange and PBE correlation potential[13,14], as implemented in the VASP code.[15] We used a cut-off energy of 500eV for the plane wave expansion of the wave functions, and fully relaxed all atoms of unit cells until the Hellman-Feynman forces are less than 0.01 eV $Å^{-1}$. For Brillouin zone integration, we used a 6×6×6 Monkhorst-Pack grid of k-points[16] in the calculations of unit cell relaxation. The unit cell of $MA_{0.5}FA_{0.5}Pb_{0.5}Sn_{0.5}I_3$ was constructed using a 2×2×2 supercell of cubic $MAPbI_3$ and $FAPbI_3$. We examined eight different configurations of Pb sites substituted by Sn ions, as shown in Supplementary Fig. 6, where the substitution of (c1) - (c4) is ordered, while that of the (c5) - (c8) in Supplementary Fig. 6 is random. We calculated the total energies of these configurations, which are displayed in Supplementary Fig. 7. Our findings indicate that c3 is the most stable configuration due to its lower total energy, and we used this configuration to perform electronic structure calculations (Supplementary Fig. 8a). The calculated bandgap is around 1.35 eV and is located near the Γ point, as indicated by the two arrows in the Supplementary Fig. 8b. At the same quasi-momentum k point, a sub band at around $2E_g$ is observed, as indicated by the upper arrows. The sub band at same k point provides a possibility for asymmetric excitation in the Pb-Sn mixed perovskite and transfers its excess energy completely to electrons in the conduction band, which is consistent with a previous report.[10] Fulfilling both energy conservation and momentum conservation, the CM threshold could decrease to $2E_g$. Thus, the asymmetric excitation due to additional sub bands at ~ $2E_g$ could be a possible reason for the low CM threshold.

5. Calculation of Internal Quantum Efficiency (IQE) and the absorbed photon flux (I_A)

The strongest evidence of CM in a solar cell is the external quantum efficiency (EQE) or IQE exceeding 100% without extra gain and at zero bias.[17] The EQE represents the ratio of the population of external circuit charge carriers produced by the solar cell to the corresponding number of incident photons on the device. The IQE represents the ratio of the population of external circuit charge carriers produced by the solar cell to the corresponding number of photons absorbed by the active layer of the device.[18] Presently, there are no reports of CM effects on the EQE of PSCs. Assessment of the IQE may provide a clearer picture of the intrinsic properties of the absorber layer to uncover the complex interplay of factors stymieing its contribution.

The IQE is computed by dividing the number of electrons produced by the number of photons absorbed. To quantify the number of photons absorbed by the perovskite layer and reduce the effect of the parasitic absorption of the preceding inactive layers, the reflectance (*R*) of the entire device and the transmittance (*T*) of the ITO/HTL layers were measured (Supplementary Fig. 10-11). *R* consists of both the fraction of light reflected by the glass that would not reach the perovskite layer and that escapes from the device due to reflection of the silver electrode. Hence, (1 - *R*) represents the fraction of the light absorbed by the whole device. The absorption by C_{60}/BCP/Ag, which is beneath the perovskite layer, is almost negligible due to the large absorption coefficient of the latter, while the transmittance of the ITO/HTL layer in front of the perovskite layer must be considered (Supplementary Fig. 9).

Thus, the IQE of the perovskite layer is derived as Equation 1 $\mathrm{IQE} = \frac{J_{\mathrm{sc}}/e}{I_{\mathrm{A}}} = \frac{J_{\mathrm{sc}}A/e}{I_{\mathrm{A}}A} = \frac{J_{\mathrm{sc}}A/e}{\mathrm{Abs}_{\mathrm{Perovskite}}P/h\nu} = \frac{\mathrm{EQE}}{\mathrm{Abs}_{\mathrm{Perovskite}}} = \frac{\mathrm{EQE}}{(1-R)T_{\mathrm{ITO\_HTL}}}$ in main text and the calculation of absorbed photon flux I_{A} using monochromatic illumination is as follows:

$$I_A = \frac{\mathrm{Abs}_{\mathrm{Perovskite}}P}{Ah\nu} = \frac{(1-R)T_{\mathrm{ITO\_HTL}}P}{Ah\nu} \quad \text{(S5)}$$

where $\mathrm{Abs}_{\mathrm{Perovskite}}$ is the fraction of light absorbed by perovskite, R is the reflectance of the whole device, $T_{\mathrm{ITO\_HTL}}$ is the transmittance of the ITO_HTL, P is the power of incident light, $h\nu$ is the photon energy, A is the active area of the PSC.

6. A more appropriate criterion for comparing the PSC photocurrent: power (P) versus absorbed photon flux (I_{A})

Supplementary Fig. 12-15 illustrates the effect of different photon flux/photon numbers present at the same power of monochromatic illumination with different wavelengths on the comparability of photocurrent due to the varying photon energies. Higher-energy photons contain intrinsically fewer photons per unit of power, resulting in a decrease in photocurrent (Supplementary Fig. 12). Using the relationship between photocurrent and power may lead to erroneous conclusions in an CM/MEG investigation. Thus, the short circuit current I_{sc} *vs* absorbed photons as well as J_{sc} *vs* I_{A} are more suitable metrics for CM studies (Supplementary Fig. 14).

7. Comparing PCEs under monochromatic illumination

The power conversion efficiency $\left(\mathrm{PCE} = \frac{P_{\mathrm{out}}}{P_{\mathrm{in}}} = \frac{J_{\mathrm{sc}} \cdot V_{\mathrm{oc}} \cdot FF}{P_{\mathrm{in}}}\right)$ determines the fraction of the incident power that is converted to electricity in a solar cell. The PCE is typically measured under the solar spectral irradiance AM 1.5 G conditions and at a temperature of 25 °C. The input power P_{in} is set at 1 kW m^{-2} or 100 mW cm^{-2} over the range of wavelengths under AM 1.5 conditions. To adapt this metric for comparison under monochromatic illumination, one must take note of the energy/wavelength dependence of some of the parameters in the PCE equation.

To illustrate this dependence, suppose we utilize two monochromatic light sources for excitation 3 eV (~413 nm wavelength) and 1.5 eV (~826 nm). For the same P_{in} = 100 mW cm^{-2} = 100 mJ s^{-1} cm^{-2} ≈ 6.24×10^{17} eV s^{-1} cm^{-2}, the photon flux for the 3 eV photons is ~ 2.08×10^{17} Phs s^{-1} cm^{-2}, while that for the 1.5 eV photons is ~ 4.16×10^{17} Phs s^{-1} cm^{-2}. Given that J_{sc} is proportional to the photon flux, this immediately leads to $J_{\mathrm{sc\ 3\ eV}} = 0.5\ J_{\mathrm{sc\ 1.5\ eV}}$. Assuming that V_{oc} and FF are identical, $\mathrm{PCE}_{\mathrm{3\ eV}} = 0.5\ \mathrm{PCE}_{\mathrm{1.5\ eV}}$. This means that the PCE using the 3eV source would be ½ that of the 1.5 eV source. Conversely, if we fix the absorbed photon flux to be the same, then $\mathrm{P}_{\mathrm{in\ 3\ eV}} = 2\ \mathrm{P}_{\mathrm{in\ 1.5\ eV}}$. For the same P_{out} (assuming J_{sc}, V_{oc}, and FF are the same), then $\mathrm{PCE}_{\mathrm{3\ eV}} = \frac{\mathrm{P}_{\mathrm{out\ 3\ eV}}}{\mathrm{P}_{\mathrm{in\ 3\ eV}}} = \frac{\mathrm{P}_{\mathrm{out\ 1.5\ eV}}}{2\mathrm{P}_{\mathrm{in\ 1.5\ eV}}} = \frac{1}{2}\mathrm{PCE}_{\mathrm{1.5\ eV}}$. In this case, J_{sc} is taken to be the same because in the absence of CM, for the same absorbed photon flux at different energies, PSC devices should ideally provide the same current density (Supplementary Fig. 13-14).

Supplementary Fig. 18c also shows the highest PCE for the $MAPbI_3$ reference occurs at the lowest excitation energy 1.89 eV (655 nm, 1.22 E_{g}). This is due to the abovementioned energy/wavelength dependence of the PCE. Furthermore, hot carrier losses could also contribute

to this trend, which will complicate the comparison of the PCEs. Hence due care must be taken for fair comparison of the PCEs under monochromatic excitations with different energies.

8. Evaluating the CM contribution to PSC performance (using P_{out} and P_{in})

Based on the intrinsic dependence of the parameters in the PCE expression with pump energy/wavelength discussed above, fair comparison of the PCE at different monochromatic energies is critical. Here, two approaches are used to assess CM's contribution to PSC performance.

The first method (see Supplementary Fig. 22b, e, h, k) is to normalize the absorbed photon flux I_A, then compare the produced current density (J_{sc}) and hence the output electrical power (P_{out}) at different illumination energies ($E > 2E_g$ or $E < 2E_g$). At the same absorbed photon flux I_A, high energy photons (With CM (W CM)) may create more electrons than low energy photons (Without CM (WO CM), resulting in a higher J_{sc} if these electrons are efficiently transported out. Supplementary Fig. 22e, h, k shows that the P_{out} of the $Cs_{0.05}FA_{0.5}MA_{0.45}Pb_{0.5}Sn_{0.5}I_3$ PSC device and $(FASnI_3)_{0.6}(MAPbI_3)_{0.4}$ PSC device under 2.62 eV (473 nm) illumination (W CM) is larger than that under 2.33 eV (532 nm), 1.89 eV (655 nm) or 1.58 eV (785 nm) illumination (WO CM).

The second approach (see Supplementary Fig. 22c, f, i, l) is to normalize the absorbed irradiance (i.e., same P_{in}), then compare (i) $PCE_{473\ nm}$; (ii) $PCE_{532\ nm}/1.125$; (iii) $PCE_{655\ nm}/1.385$ and (iv) $PCE_{785}/1.66$. With the same P_{in}, the lower energy illumination contains more photons (Supplementary Fig. 12), hence the normalization factor (i.e., 1.125, 1.385, 1.66) arises from absorbed photon flux $I_{A\ 532\ nm} = \sim 1.125\ I_{A\ 473\ nm}$; $I_{A\ 655\ nm} = \sim 1.385\ I_{A\ 473\ nm}$ and $I_{A\ 785\ nm} = \sim 1.66\ I_{A\ 473nm}$. When the effect of different photon numbers contained in the same P_{in} is factored out/absent,

very similar PCE plots are observed in $MAPbI_3$ reference PSCs (Supplementary Fig. 22c) while an improvement in PCE is obtained from the mixed Pb-Sn PSCs (Supplementary Fig. 22f, i, l), as expected. The contribution of CM can now be directly observed.

9. Discussion on CM performance difference in various Pb-Sn mixed devices

CM can be observed in both the PSCs based on the two Pb-Sn perovskite materials. However, $Cs_{0.05}FA_{0.5}MA_{0.45}Pb_{0.5}Sn_{0.5}I_3$ devices exhibit slightly more efficient CM than $(FASnI_3)_{0.6}(MAPbI_3)_{0.4}$ devices (Supplementary Fig. 22f, i). To examine the origin of the CM performance difference, we have also measured the hot carrier cooling (HCC) behaviour and calculated the hot carrier temperatures and HCC times for both materials according to previous publications.[19-21]

Supplementary Fig. 23a, b shows the TA spectra of $Cs_{0.05}FA_{0.5}MA_{0.45}Pb_{0.5}Sn_{0.5}I_3$ and $(FASnI_3)_{0.6}(MAPbI_3)_{0.4}$ thin films at time delays between 0.24 and 5 ps. For fair comparison, the absorbed photon flux (I_A) for both measurements are kept around 6.8×10^{12} cm^{-2} and the exciton pump energy is approximately 2.18 eV (570 nm). The carrier temperature can be fitted according to the following equation:[19]

$$\Delta A = (1 - f_e(E, E_F, T_c))^2 \times A_{exc} - A_0 \quad \text{(S6)}$$

where ΔA is the TA signal, f_e is the Fermi−Dirac distribution function, E_F is the quasi-Fermi energy, T_c is the carrier temperature, $A_0 \equiv (E, E_g, \Gamma)$ and $A_{exc} \equiv (E, E_g - \Delta E_g, \Gamma')$ are the absorption spectra before and after excitation, E_g is the bandgap, ΔE_g is the bandgap change as a result of bandgap renormalization (BGR) , Γ and Γ' are the line widths before and after excitation. Supplementary Fig. 23c displays the calculated time-dependent temperature of the two materials.

The $Cs_{0.05}FA_{0.5}MA_{0.45}Pb_{0.5}Sn_{0.5}I_3$ sample shows a higher carrier temperature as compared to the $(FASnI_3)_{0.6}(MAPbI_3)_{0.4}$ sample. Then, the longitudinal optical- longitudinal acoustic (LO-LA) phonon scattering time τ_{LO} which dominates the HC cooling in perovskite is fitted with the following equation: [20,21]

$$J_{\mathrm{r}} = \frac{3}{2}\frac{h\omega_0}{\tau_{\mathrm{LO}}}(e^{\frac{h\omega_0}{kT_{\mathrm{a}}}} - e^{\frac{h\omega_0}{kT_{\mathrm{c}}}})\frac{N_{\mathrm{LO}}(T_{\mathrm{a}})}{N_{\mathrm{LO}}(T_{\mathrm{c}})}(\frac{kT_{\mathrm{c}}}{h\omega_0})^2 e^{-\frac{h\omega_0}{kT_{\mathrm{c}}}} \quad \text{(S7)}$$

where J_{r} is the energy loss rate per carrier, $h\omega_0$ is the LO-phonon energy, τ_{LO} is the characteristic LO-phonon decay time, T_{a} is the acoustic phonon temperature, T_{c} is carrier temperature, $N_{\mathrm{LO}}(T)$ is the LO-phonon occupation number at temperature T. Under 2.18 eV (I_A = 6.8×10^{12} cm^{-2}) pump excitation, the τ_{LO} for $Cs_{0.05}FA_{0.5}MA_{0.45}Pb_{0.5}Sn_{0.5}I_3$ and $(FASnI_3)_{0.6}(MAPbI_3)_{0.4}$ are ~ 318 fs and ~ 248 fs, respectively.

Similarly, HC temperatures and HCC lifetimes of the two materials under 1.55 eV pump excitation (800 nm, I_A = 8×10^{12} cm^{-2}) are fitted from the TA spectra (Supplementary Fig. 23d, e) using Supplementary equation 6 and 7. $Cs_{0.05}FA_{0.5}MA_{0.45}Pb_{0.5}Sn_{0.5}I_3$ also shows a higher hot carrier temperature (Supplementary Fig. 23f) and a slower HC cooling (τ_{LO} ~ 328 fs) compared to $(FASnI_3)_{0.6}(MAPbI_3)_{0.4}$ (τ_{LO} ~ 228 fs).

Since HCC is a competing process with CM, a slower HCC process may thus be more favourable for CM.[4] The more efficient CM could possibly arise from the higher hot carrier (HC) temperature and the slower hot carrier cooling (HCC) in $Cs_{0.05}FA_{0.5}MA_{0.45}Pb_{0.5}Sn_{0.5}I_3$. This could be due to the addition of alkali cation Cs^+. By adding 5% Cs^+ into the pure FAMA perovskite, an increase in the initial carrier temperature was also reported in the pure lead perovskite system.[22] The alkali cations could passivate halide vacancies, improve the lattice strain relaxation, reduce hot photon bottleneck threshold, elevate carrier temperature and modify hot carrier relaxation and transport.[22]

The appropriate doping of alkali cations such as Cs^+, Rb^+, K^+ provides an opportunity for designing perovskite materials with slower hot carrier cooling and higher CM efficiency.

10. Increased carrier recombination loss in thicker perovskite layers and parasitic absorption loss from glass and ITO

Comparing the optimized quartz-based PSCs (Quartz/ITO/2PACz/Perovskite/C_{60}/BCP/Ag structure), the glass-based PSCs (Glass/ITO/PEDOT: PSS/ Perovskite/C_{60}/BCP/Ag structure), we clearly reveal the intricate interplay of various factors influencing PCE gains from CM effects. Given the wavelength dependence of CM, perovskite layer thickness is a critical consideration in devices. Wavelength dependence of the penetration depth of solar photons is the first consideration. Supplementary Fig. 24a shows the EQE and integrated J_{sc} for the glass/ITO/PEDOT:PSS/$Cs_{0.05}FA_{0.5}MA_{0.45}Pb_{0.5}Sn_{0.5}I_3$ based devices with different active layer thicknesses. For long-wavelength photons (~700 – 1000 nm) with long penetration depth, there is a big increase in EQE from 200 nm to 260 nm thickness because of insufficient absorption by the thinner perovskite layer (Supplementary Fig. 25b). For short-wavelength photons ~530 nm with shorter penetration depth, EQE remains almost invariant (from 200 – 340 nm) and then drops for thicker layers (Supplementary Fig. 24b). Similarly, at an even shorter wavelength of 360 nm, the EQE trend falls continuously from ~75.9% to ~67.5% with increasing perovskite thickness (Supplementary Fig. 24b), which is likely because of increased carrier loss in the thicker perovskite layer (Supplementary Fig. 25a). Thus, in the thicker samples, such carrier loss may negate any CM/MEG effect at these short-wavelength regions with shorter penetration depths. Another factor affecting the PCE enhancements from CM is losses resulting from parasitic absorption. Compared to the

glass substrate, the quartz substrate has greater transmittance in the UV region (Supplementary Fig. 26).

11. Discussion on the trend of IQE in the PSCs.

In CM, there is an interplay of the photon penetration depth and the CM quantum yield (QY) which are both energy/wavelength dependent. The IQE of our PSC device is determined by two factors: (1) the population of generated carriers and (2) carrier losses during transport and extraction. For (1), the CM process may help generate more carriers, thus resulting in an enhanced IQE. For (2), the recombination losses during transport and extraction may lead to the inefficient carrier collection, resulting in a reduced IQE.

For energy $E < 2E_g$ (single exciton regime, Supplementary Fig. 37a), the photon penetration depth into the perovskite absorber is long, thus there is more efficient charge extraction to the electrodes. Hence, single exciton IQEs are nearly 100%. For $2E_g < E < 3E_g$, the photon penetration depth into the perovskite absorber is reduced (Supplementary Fig. 37b). Hence, there is now a competing effect between the effective charge extraction and the CM quantum yield (i.e., the step-like plot in Figure 1d). This results in the IQE increasing to ~110% and does not increase further (and could even decrease due to poor charge extraction) until $> 3E_g$ (Supplementary Fig. 37b). Beyond $3E_g$, there is a dominant increase in CM QY that further increases the IQE (Supplementary Fig. 37c, Figure 1d). Nevertheless, losses from defects at grain boundaries and at interfaces during transport and extraction are still present.

The Auger recombination loss may also contribute to the observed discrepancy between the IQE and the expected values from the TA spectra, leading to the observed variation trend of IQE. As shown in Supplementary Fig. 38, we analysed the TA spectra of the $Cs_{0.05}FA_{0.5}MA_{0.45}Pb_{0.5}Sn_{0.5}I_3$ pumped with different pump energies to investigate the change in the Auger recombination behaviour. The initial intensity ΔA is controlled to be around 3 mOD to make sure the population of carriers pumped under different pump energies is about the same. Besides, when initial ΔA remains constant at 3 mOD, the pump fluence of the high-energy photons is lower (e.g., the fluence for the 606 nm pump is 2.80 μJ cm^{-2}, while the fluence for the 300 nm pump is 1.65 μJ cm^{-2}). The spectra were fitted by bi-exponential decay convolved with a gaussian function, which can be expressed by:

$$y = y_0 + f(x) * h(x) \tag{S8}$$

Where $f(x) = \frac{A_1}{\tau_1} e^{-\frac{x}{\tau_1}} + \frac{A_2}{\tau_2} e^{-\frac{x}{\tau_2}}$, representing the bi-exponential decay process and $h(x)$ is the Gaussian function which indicates the instrument response function (IRF). Here, τ_1 represent the biexciton Auger recombination time and τ_2 represent the exciton recombination time. The fitted values are summarized in Supplementary Table 3. It can be found that when the photon energy is smaller than $2E_g$, their TA kinetics and Auger recombination time τ_1 are similar (Supplementary Fig. 39). When the photon energy exceeds $2E_g$, their Auger recombination becomes faster with the increase of photon energy, even though the pump fluence of the high-energy photon is lower (e.g., the fluence for the 606 nm pump is 2.80 μJ cm^{-2}, while the fluence for the 300 nm pump is 1.65 μJ cm^{-2}), which can be attributed to the presence of CM (Supplementary Fig. 39). This severe Auger recombination hinders the efficient extraction of the generated carriers, resulting in a lower IQE observed in PSC devices. When the photon energy exceeds $2.5E_g$, the Auger recombination

keep becomes faster and the ratio of the Auger recombination becomes dominant. Thus, in the energy range between 2.5E_g and 3E_g, the IQE may even show a decline. When the photon energy exceeds 3E_g, both the Auger recombination time τ_1 and Auger recombination ratio remain almost invariant, while the CM QY continues to increase due to CM. Thus, the IQE obtained from the PSC devices starts to rise again. Meanwhile, as shown in Supplementary Fig. 40, the exciton recombination also becomes faster as the increase of photon energy. This is consistent with our previous discussion about the increased carrier recombination for charges generated by higher energy photons.

Therefore, the observed variation trend in IQE values of the PSCs, as well as their lower-than-ideal IQE values, could be attributed to recombination losses originating from both Auger recombination and carrier recombination in the perovskite films.

12. Ellipsometry measurements and Optical modelling.

The complex index of refraction $N(\lambda) = n(\lambda) + ik(\lambda)$ spectra for perovskite films, ITO and C_{60} were determined using an α-SE^{TM} spectroscopic Ellipsometer (J. A. Woollam Co., Inc.) at various incident angles ranging from 65° to 75° with an increment of 5°. The ellipsometry measuring range is from 381 nm to 893 nm.

Optical modelling was performed using the Solcore python package.[23] Custom materials were defined with n and k parameters obtained from the ellipsometry measurements. The device stack was constructed with the SolarCell class, and the absorption profile of the device stack was calculated with the transfer matrix method (TMM) solver. The absorptance of the perovskite layer

was then obtained by integrating the absorption profile over the stack, i.e., $\int A(\lambda)dz$. The modelled IQE (Supplementary Fig. 41) was then computed by taking the experimental EQE divided by the calculated absorptance.

Supplementary Figures

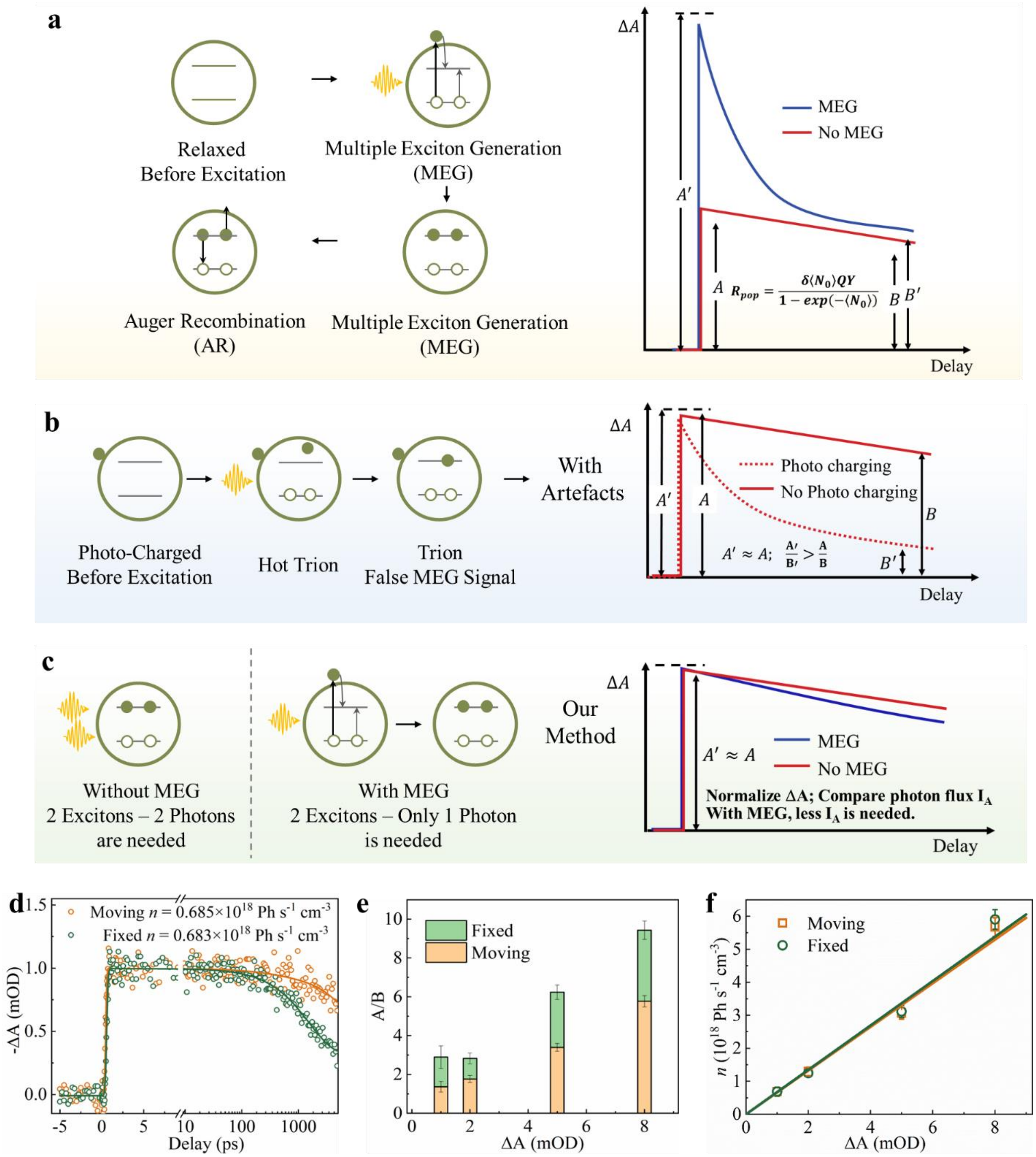

Supplementary Fig. 1| CM/MEG artifacts in perovskite thin films from TA (fixed vs moving sample). (a). Schematic of the CM/MEG process and the traditional method for assessing CM/MEG using the initial amplitude A

and long-lived quasi-equilibrium amplitude B. With CM/MEG (blue line - pumping above CM/MEG threshold) and No CM/MEG (red line – pumping below CM/MEG threshold) **(b).** Schematic of false CM/MEG signals caused by the existence of surface traps. The red lines (solid and dashed) represent excitation below the CM/MEG threshold. **(c).** The straightforward method compares the required absorbed photon flux I_A at the same change in absorbance ΔA_{max} for different excitation energies (Blue line = above CM threshold and Red line = below CM threshold). With CM, less I_A is needed to reach the same ΔA_{max}. **(d).** Photo-bleaching transients of $(FASnI_3)_{0.6}(MAPbI_3)_{0.4}$ sample with strong photo-charging (sample fixed) or suppressed photo-charging (sample moving). **(e).** Exciton population ratio A/B as a function of $|\Delta A_{max}|$ for a fixed or moving sample when pumped at below CM threshold. The error bars represent the uncertainties. **(f).** Required I_A as a function of $|\Delta A_{max}|$. The error bars indicate the measurement error.

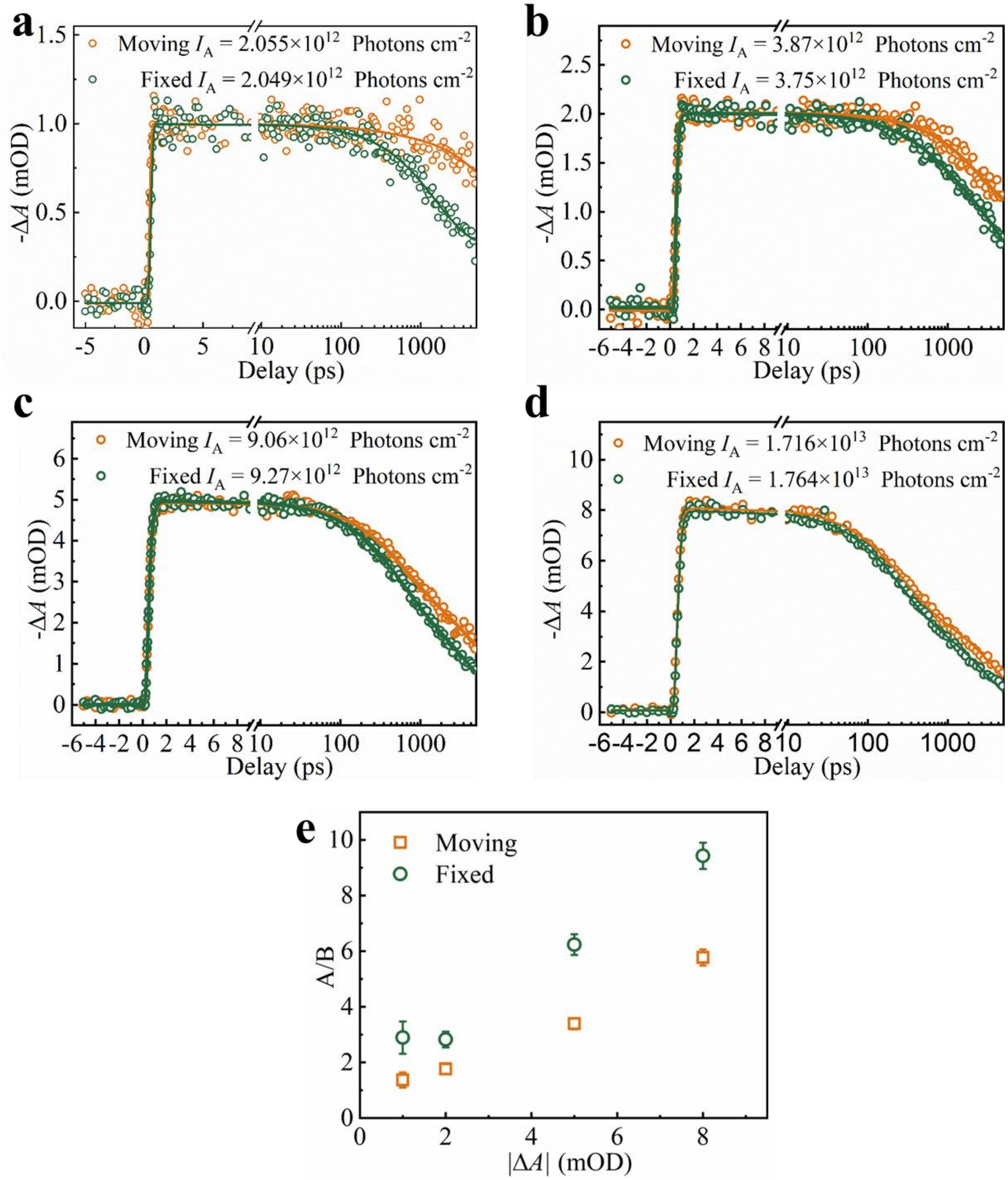

Supplementary Fig. 2| A power-dependent experiment on the variation of A/B with an increase of pump intensity in samples w/wo photo-charging effect. The corresponding Δ*A* are **(a).**1 mOD, (**b**)**.** 2 mOD, **(c).** 5 mOD, and **(d).** 8 mOD, respectively. **(e)**. The summarized A/B as a function of |Δ*A*| for fixed or moving sample when pumped at below CM threshold. The error bars represent the uncertainties.

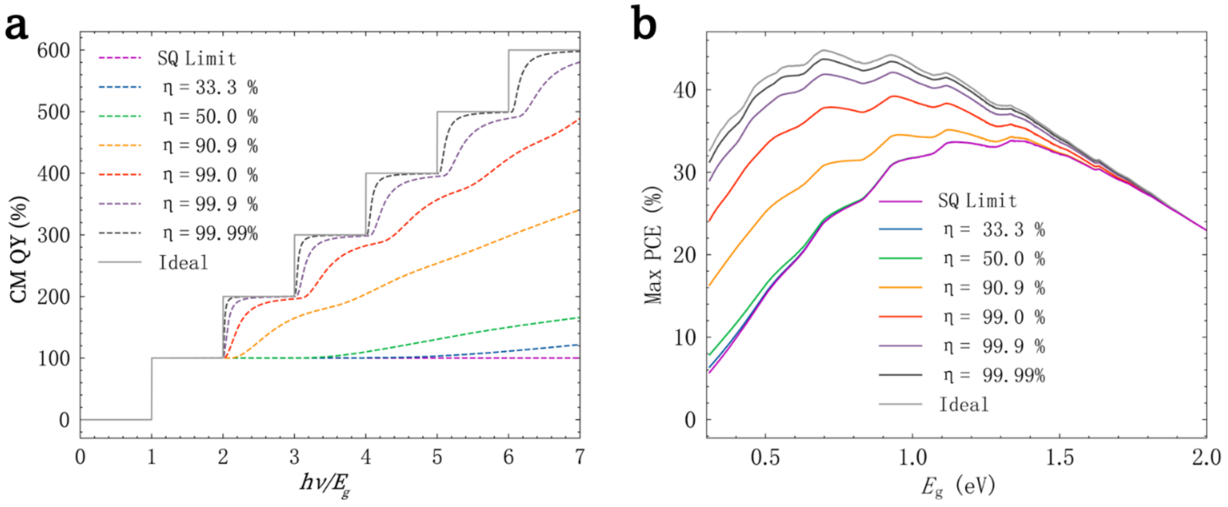

Supplementary Fig. 3| Simulated CM/MEG QY and PCE for CM/MEG solar cells. (a) Theoretical CM QY as a function of $h\nu/E_g$ with different η. (b) The corresponding maximum PCE under one sun (AM 1.5 G).[7]

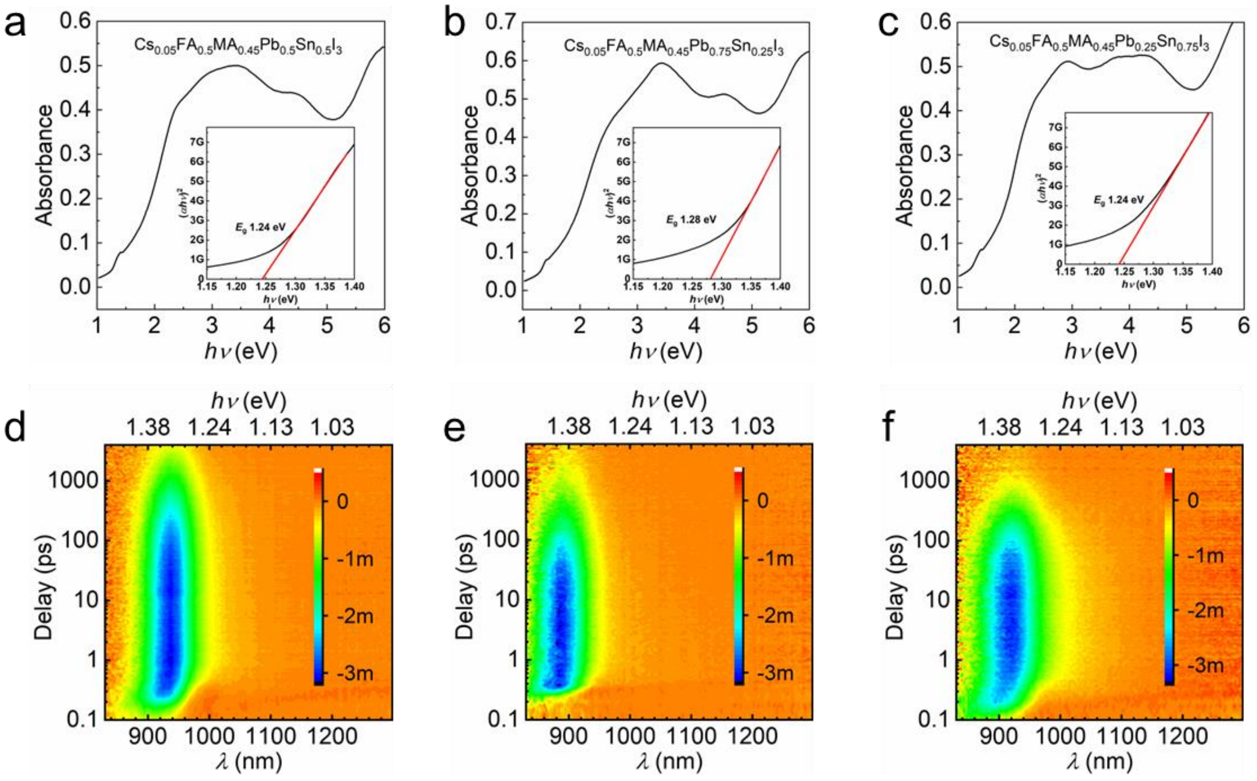

Supplementary Fig. 4| Optical spectroscopy of $Cs_{0.05}FA_{0.5}MA_{0.45}Pb_{0.5}Sn_{0.5}I_3$, $Cs_{0.05}FA_{0.5}MA_{0.45}Pb_{0.75}Sn_{0.25}I_3$ and $Cs_{0.05}FA_{0.5}MA_{0.45}Pb_{0.25}Sn_{0.75}I_3$. (a-c). Steady-state absorption spectra of: (a). $Pb_{0.5}Sn_{0.5}$, (b). $Pb_{0.75}Sn_{0.25}$, (c) $Pb_{0.25}Sn_{0.75}$ samples. Inset is the Tauc plot indicating the bandgap. **(d-f).** TA spectra of: (d). $Pb_{0.5}Sn_{0.5}$, (e). $Pb_{0.75}Sn_{0.25}$, (f) $Pb_{0.25}Sn_{0.75}$ samples.

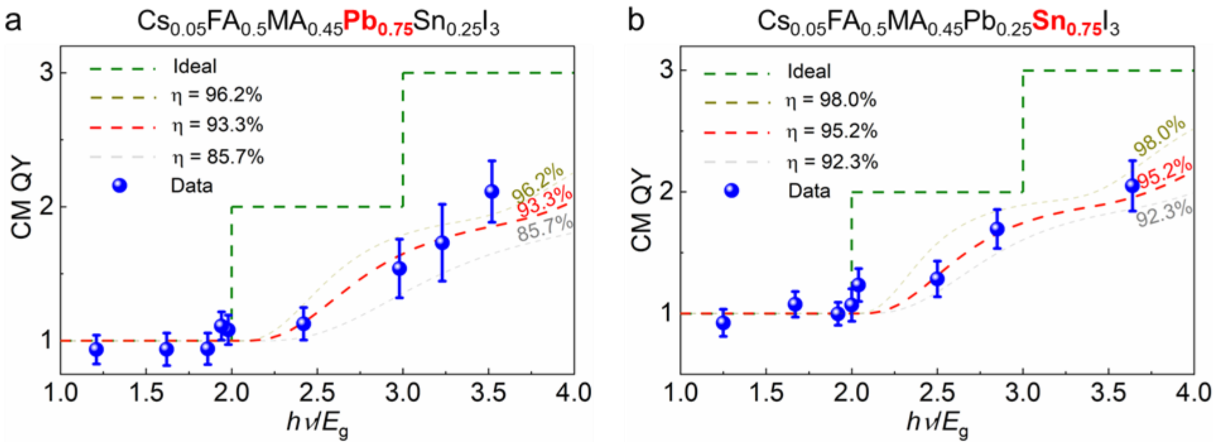

Supplementary Fig. 5| CM QY and CM efficiency of $Cs_{0.05}FA_{0.5}MA_{0.45}Pb_{0.75}Sn_{0.25}I_3$ and $Cs_{0.05}FA_{0.5}MA_{0.45}Pb_{0.25}Sn_{0.75}I_3$. (a). Variation of CM QY as a function of $h\nu/E_g$ in $Cs_{0.05}FA_{0.5}MA_{0.45}Pb_{0.75}Sn_{0.25}I_3$. The CM threshold is around 2.10E_g. **(b).** Variation of CM QY as a function of $h\nu/E_g$ in $Cs_{0.05}FA_{0.5}MA_{0.45}Pb_{0.25}Sn_{0.75}I_3$. The CM threshold is around 2.05E_g. The error bars in (a) and (b) represent the uncertainties determined using error propagation formula.

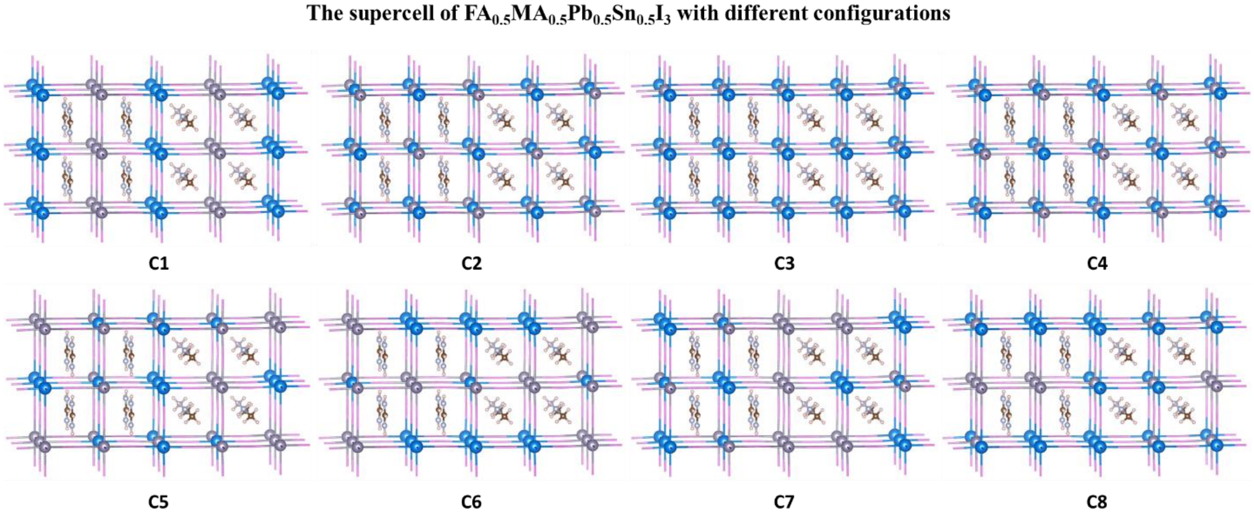

Supplementary Fig. 6| The supercell of $FA_{0.5}MA_{0.5}Pb_{0.5}Sn_{0.5}I_3$ with different configurations. The different configurations of Pb sites substituted by Sn ions, where the substitution in C1-C4 is ordered, while that for C5-C8 is random. Pb: blue atoms; Sn: violet atoms.

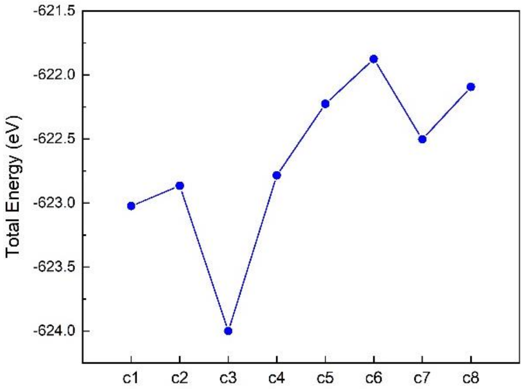

Supplementary Fig. 7| The calculated total energies of $MA_{0.5}FA_{0.5}Pb_{0.5}Sn_{0.5}I_3$ with different configurations.

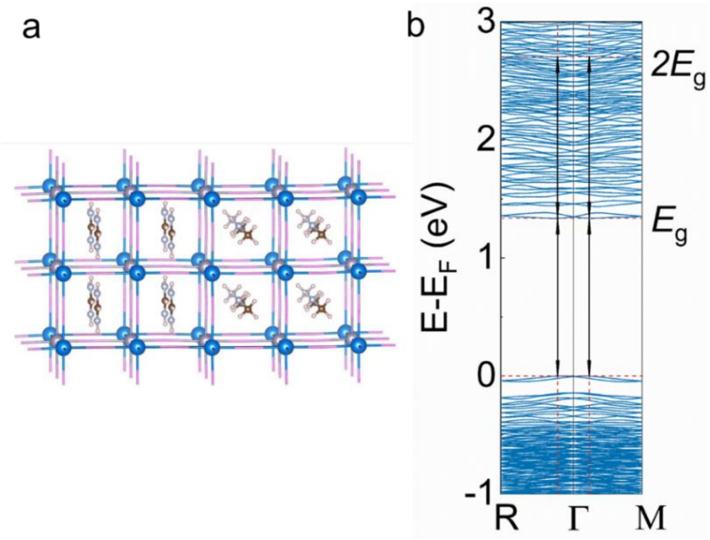

Supplementary Fig. 8| The supercell and the electronic band structure of $FA_{0.5}MA_{0.5}Pb_{0.5}Sn_{0.5}I_3$. (a). The supercell of $FA_{0.5}MA_{0.5}Pb_{0.5}Sn_{0.5}I_3$ with cubic phase. **(b).** Electronic band structure of $FA_{0.5}MA_{0.5}Pb_{0.5}Sn_{0.5}I_3$.

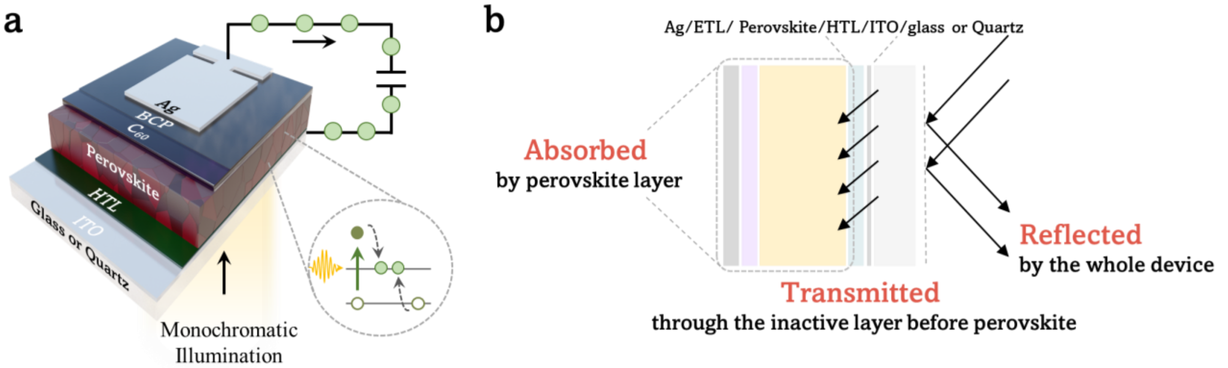

Supplementary Fig. 9| CM study under monochromatic illumination. (a). Schematic of the PSC structure and illustration of the CM effect under monochromatic illumination. **(b).** Schematic illustration of the considerations for

the computation of the IQE of the device. The reflection and transmission of photons through inactive layers were accounted for in order to compute the absorbed fraction of photons by the perovskite layer. R is the reflection of the whole device; T is the transmittance of the ITO_HTL before the perovskite layer. T was measured using glass/quartz as the baseline to minimize influence from the reflection of the glass/quartz substrate to extract the T of the ITO_HTL only. $(1 - R) \times T$ represents the portion absorbed by the perovskite layer. IQE of perovskite absorbing layer is defined as $\text{IQE} = \text{EQE}/[(1 - R) \times T]$.

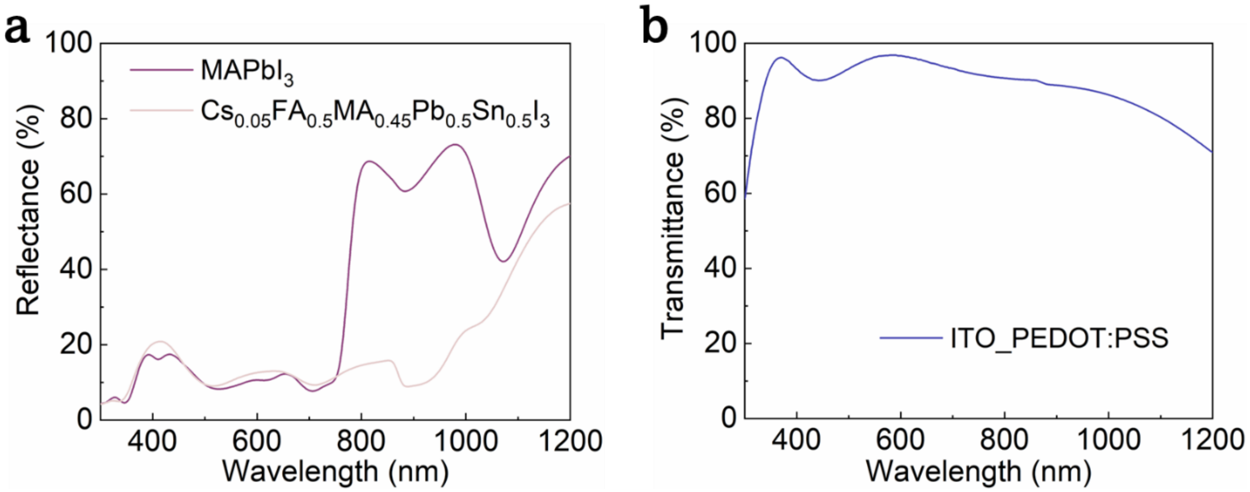

Supplementary Fig. 10| Optical properties of the PEDOT:PSS based PSCs. (a). Reflectance of the Pb based (1.55M 340 nm) and Pb-Sn mixed (1.6M 340 nm) PSCs based on the structure of glass/ITO/PEDOT:PSS/perovskite/C_{60}/BCP/Ag. **(b).** Transmittance of ITO_PEDOT: PSS layer. T is measured using glass as the baseline to minimize influence from reflection of glass substrate in order to extract the T of the ITO_PEDOT:PSS only.

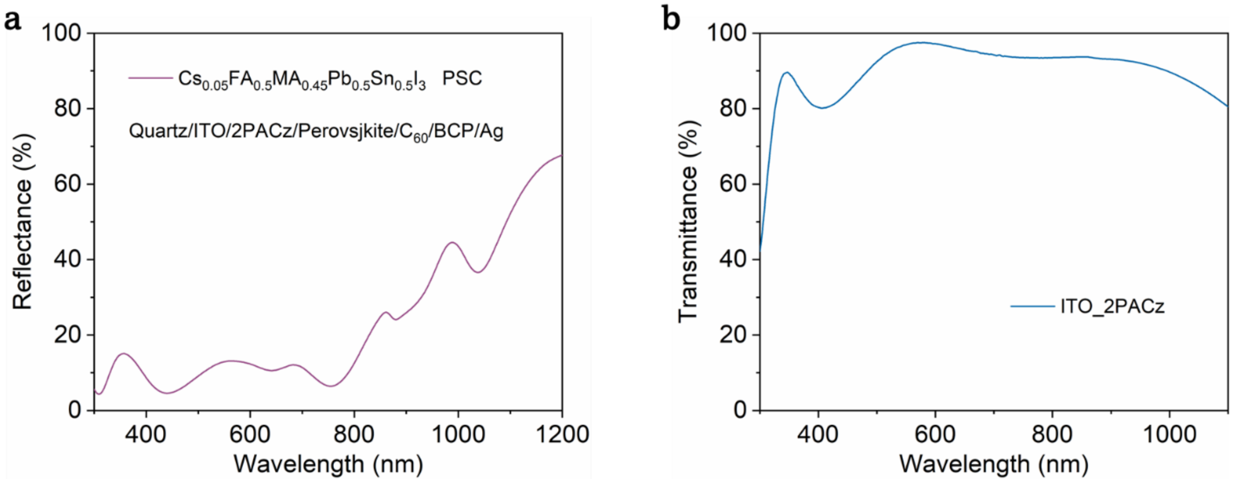

Supplementary Fig. 11| Optical properties of the 2PACz based PSCs. (a). Reflectance of Pb-Sn mixed PSCs based on the structure of Quartz/ITO/2PACz/perovskite/C_{60}/BCP/Ag (1.6M 400 nm). **(b).** Transmittance of ITO_2PACz layer. *T* is measured using quartz as the baseline to minimize influence from reflection of quartz substrate in order to extract the *T* of the ITO_2PACz only.

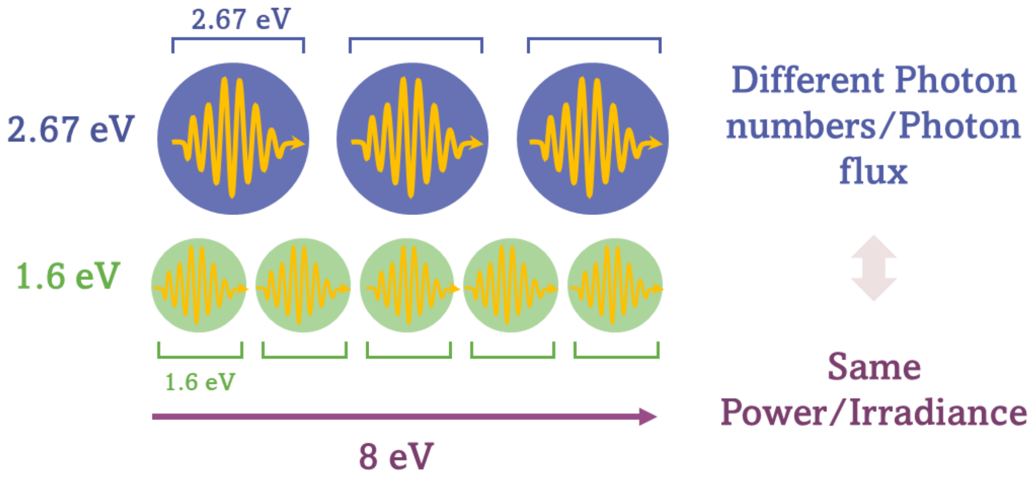

Supplementary Fig. 12| Relationship between photon energies and photon flux. Schematic illustration of an example showing that for the same power/ irradiance, the photon numbers/ photon flux of photons with different energies are different.

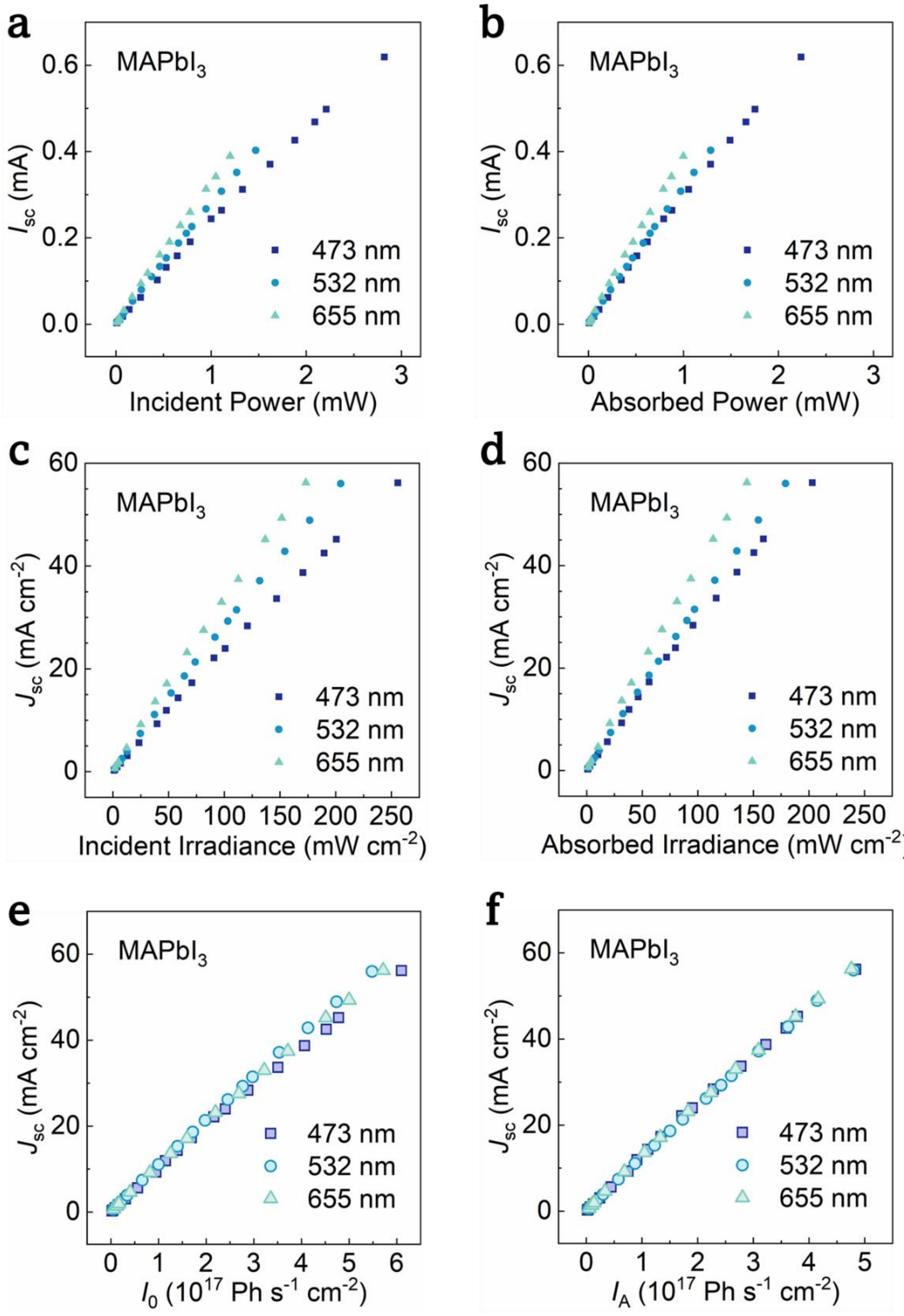

Supplementary Fig. 13| I_{sc} and J_{sc} as a function of power and photon flux in Pb based PSCs. (a-b). I_{sc} as a function of **(a)** incident power, **(b)** absorbed power. **(c-f)**. J_{sc} as a function of **(c)** incident irradiance, **(d)** absorbed irradiance, **(e)** incident photon flux and **(f)** absorbed photon flux in Pb based PSCs.

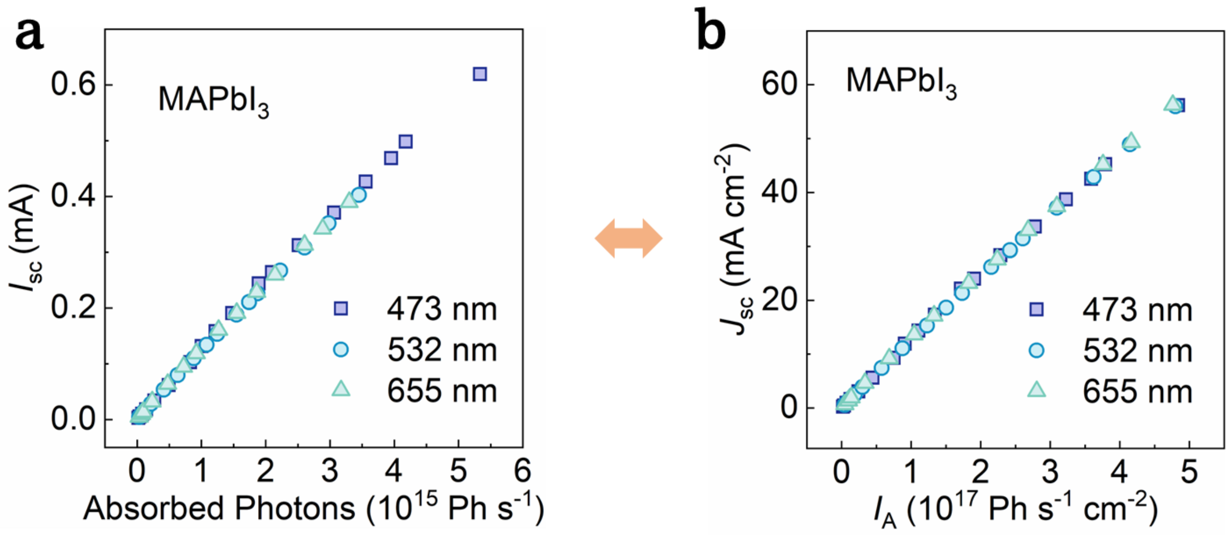

Supplementary Fig. 14| Variation of I_{sc} as a function of absorbed photon and variation of J_{sc} as a function of absorbed photon flux I_A are synonymous. (a). Variation of I_{sc} as a function of absorbed photon. **(b)**. Variation of J_{sc} as a function of I_A.

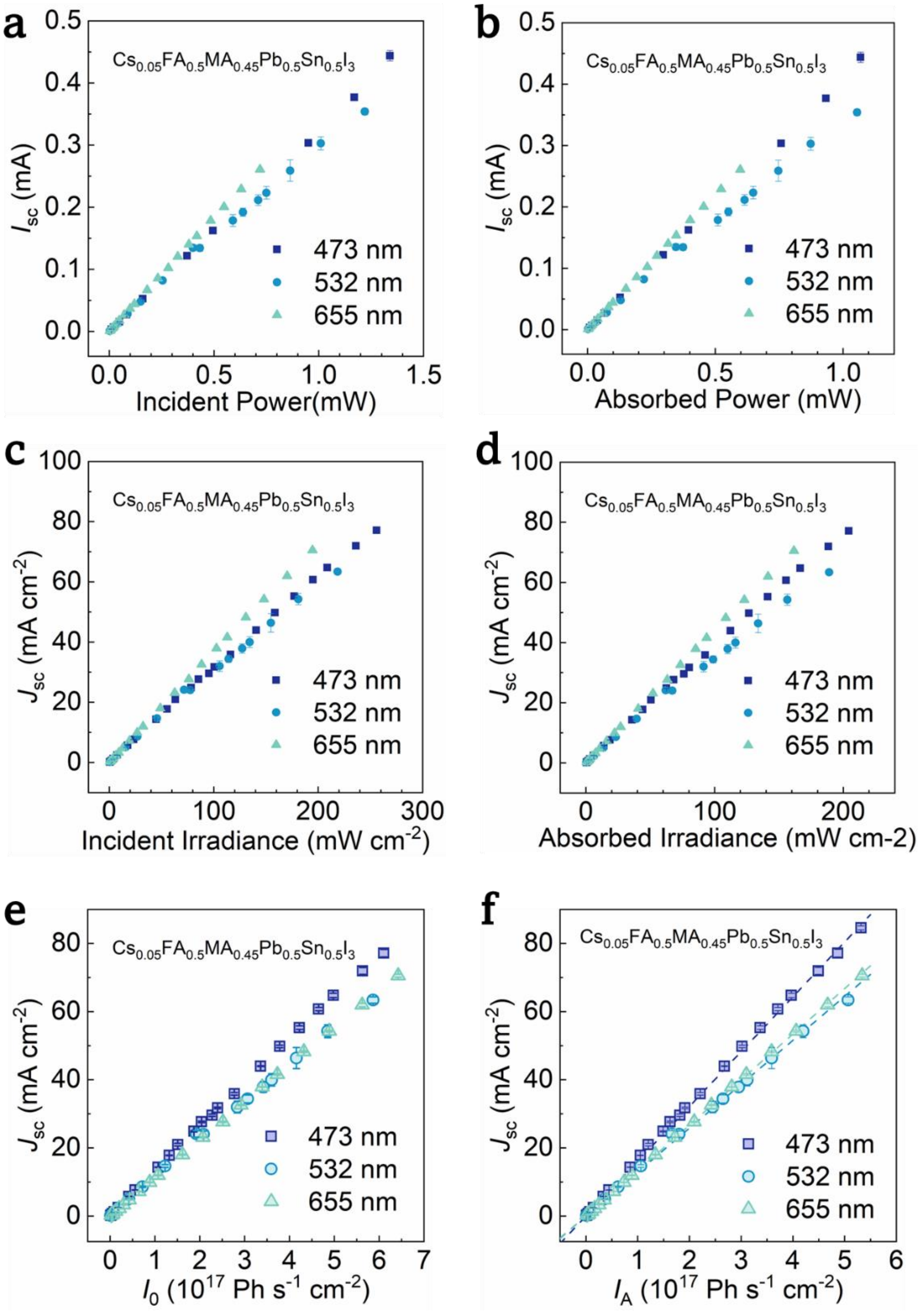

Supplementary Fig. 15| I_{sc} and J_{sc} as a function of power and photon flux in Pb-Sn mixed PSCs based on glass/ITO/PEDOT: PSS structure. (a-b). I_{sc} as a function of **(a)** incident power, **(b)** absorbed power. **(c-f)**. J_{sc} as a function of **(c)** incident irradiance, **(d)** absorbed irradiance, **(e)** incident photon flux and **(f)** absorbed photon flux in Pb-Sn mixed PSCs. The error bars represent the standard deviation.

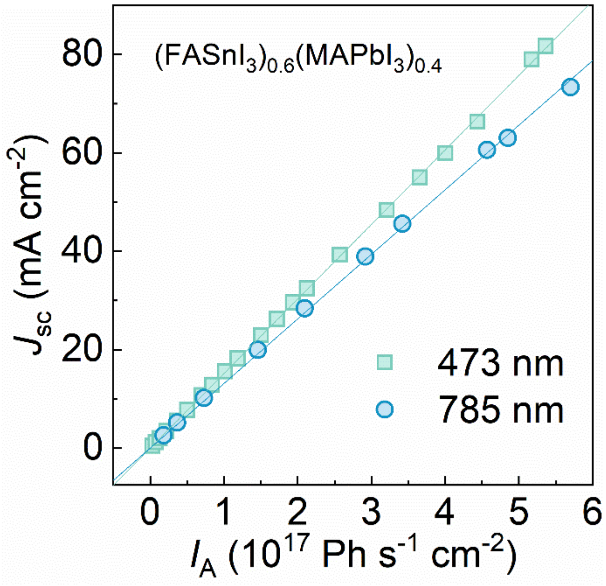

Supplementary Fig. 16| CM measurement using monochromatic illumination in $(FASnI_3)_{0.6}(MAPbI_3)_{0.4}$ PSCs.
J_{sc} under monochromatic illumination in $(FASnI_3)_{0.6}(MAPbI_3)_{0.4}$ PSCs.

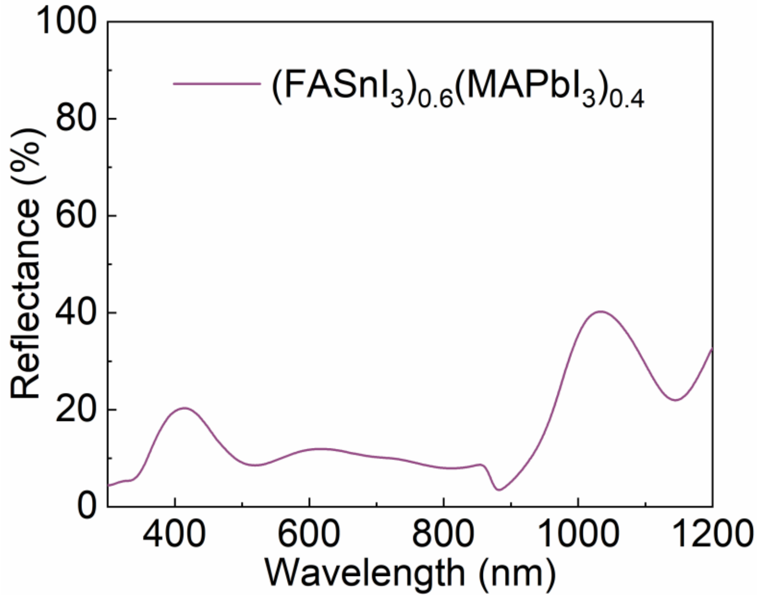

Supplementary Fig. 17| Reflectance of $(FASnI_3)_{0.6}(MAPbI_3)_{0.4}$ PSCs.

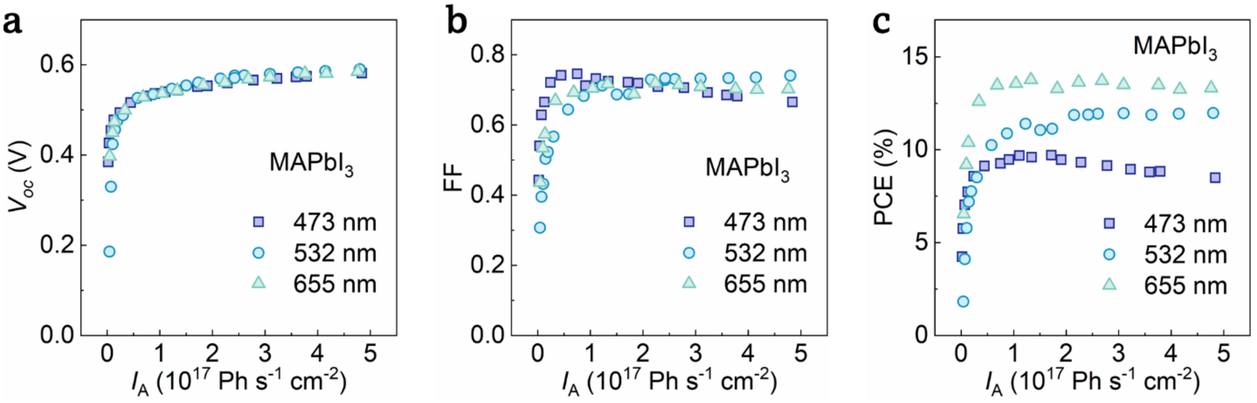

Supplementary Fig. 18| Other performance metrics of glass/ITO/PEDOT: PSS/$MAPbI_3$ Pb-based PSC. (a). V_{oc}, **(b).** FF and **(c).** PCE of $MAPbI_3$ based PSCs under monochromatic illumination.

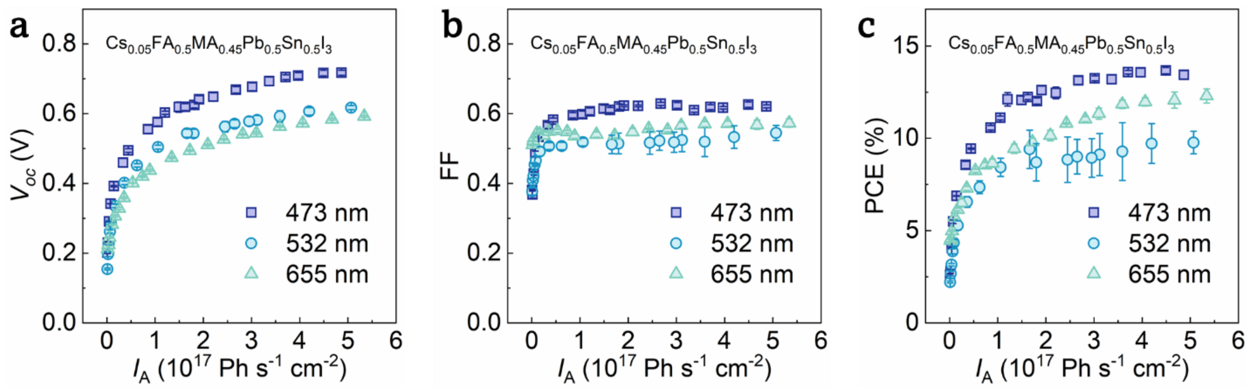

Supplementary Fig. 19| Other performance metrics of glass/ITO/PEDOT: PSS/$Cs_{0.05}FA_{0.5}MA_{0.45}Pb_{0.5}Sn_{0.5}I_3$ mixed Pb-Sn PSC. (a). V_{oc}, **(b).** FF and **(c).** PCE of $Cs_{0.05}FA_{0.5}MA_{0.45}Pb_{0.5}Sn_{0.5}I_3$ mixed PSCs under monochromatic illumination. The error bars represent the standard deviation.

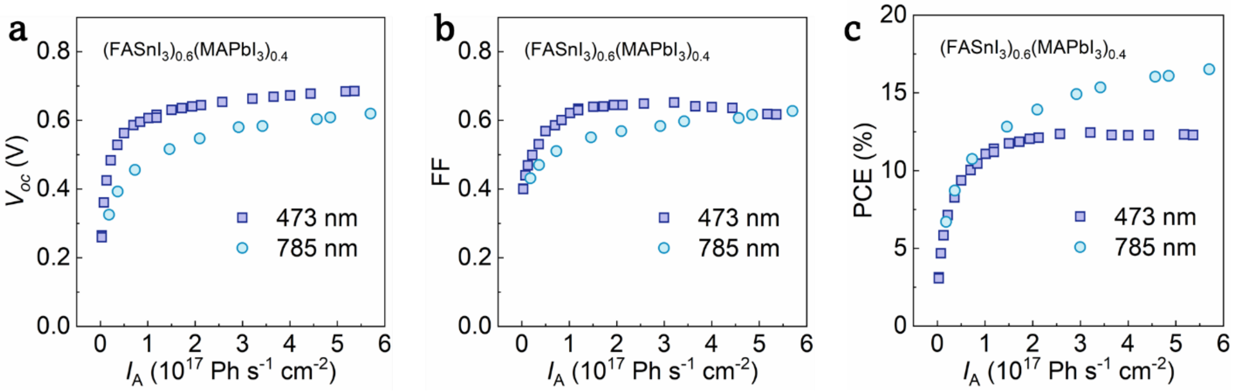

Supplementary Fig. 20| Other performance metrics of glass/ITO/PEDOT: PSS/$(FASnI_3)_{0.6}(MAPbI_3)_{0.4}$ mixed Pb-Sn PSC. (a). V_{oc}, **(b).** FF and **(c).** PCE of $(FASnI_3)_{0.6}(MAPbI_3)_{0.4}$ mixed PSCs under monochromatic illumination. Do note the energy difference between 473 nm photons and 785 nm photons which results in different P_{in} for same photon flux. Although the J_{sc}, V_{oc} and FF of the 473 nm photons are larger than that of the 785 nm photons, the PCE ($=P_{out}/P_{in}$) of the 473 nm photons is still lower. Hence for fair comparison of the PCE obtained under monochromatic illumination with different energies, a normalization is required (see Supplementary Fig. 22i). Refer to Supplementary Note 7-8 for more details.

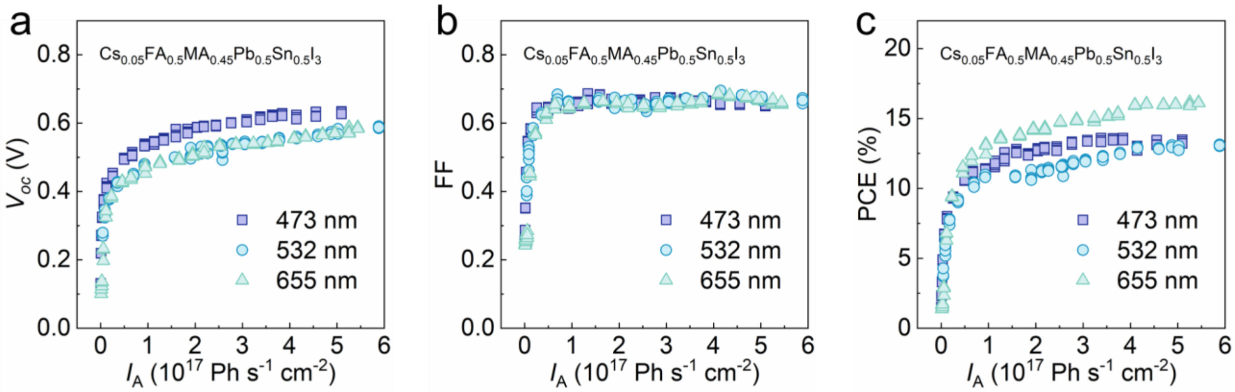

Supplementary Fig. 21| Other performance metrics of Quartz/ITO/2PACz/$Cs_{0.05}FA_{0.5}MA_{0.45}Pb_{0.5}Sn_{0.5}I_3$ mixed Pb-Sn PSC based on quartz substrate and SAM 2PACz. (a). V_{oc}, **(b).** FF and **(c).** PCE of $Cs_{0.05}FA_{0.5}MA_{0.45}Pb_{0.5}Sn_{0.5}I_3$ mixed PSCs under monochromatic illumination.

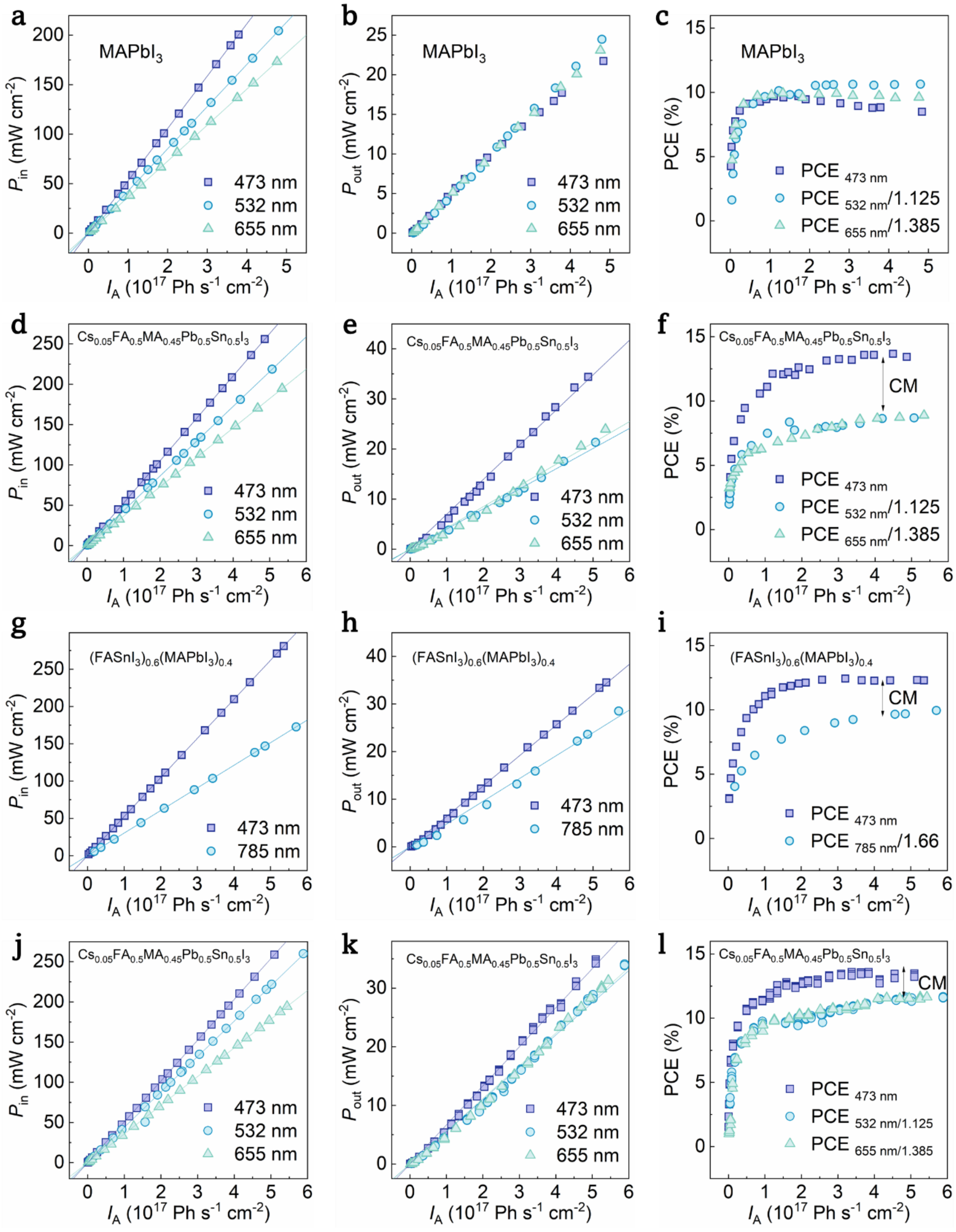

Supplementary Fig. 22| Assessment of CM contributions to PCE under monochromatic illumination. (a). (d). (g). (j) P_{in}, **(b)**, **(e)**, **(h). (k).** P_{out} and **(c). (f). (i). (l).** normalized PCEs of (**a-c**) $MAPbI_3$ PSCs, (**d-f**) glass/ITO/PEDOT: PSS/$Cs_{0.05}FA_{0.5}MA_{0.45}Pb_{0.5}Sn_{0.5}I_3$ PSCs, (**g-i**) $(FASnI_3)_{0.6}(MAPbI_3)_{0.4}$ PSCs and **(j-l).** Quartz/ITO/2PACz/

$Cs_{0.05}FA_{0.5}MA_{0.45}Pb_{0.5}Sn_{0.5}I_3$ PSCs under monochromatic illumination with different energies and intensities. (P_{out} = J_{sc} × V_{oc} × FF, PCE = P_{out}/P_{in}) The arrow in (f), (i) an (l) shows the contribution of CM in Pb-Sn mixed PSCs. Conversely, there is no enhancement for $MAPbI_3$ reference in (c). A discussion on the CM performance difference for various Pb-Sn mixed devices (Supplementary Fig. 22f, i) can be found in Supplementary Note 9 and Supplementary Fig. 23. For Supplementary Fig. 22l, the IQE at 532nm and 655 nm increased from ~80% to ~95% possibly due to further optimization of the PSC structure and processing (e.g., quartz/ITO, 2PACz, vacuum-assisted crystallization etc.), resulting in a smaller PCE increase compared to Supplementary Fig. 22f. Nonetheless, the PCE increase due to CM is consistently present in all the Pb-Sn devices.

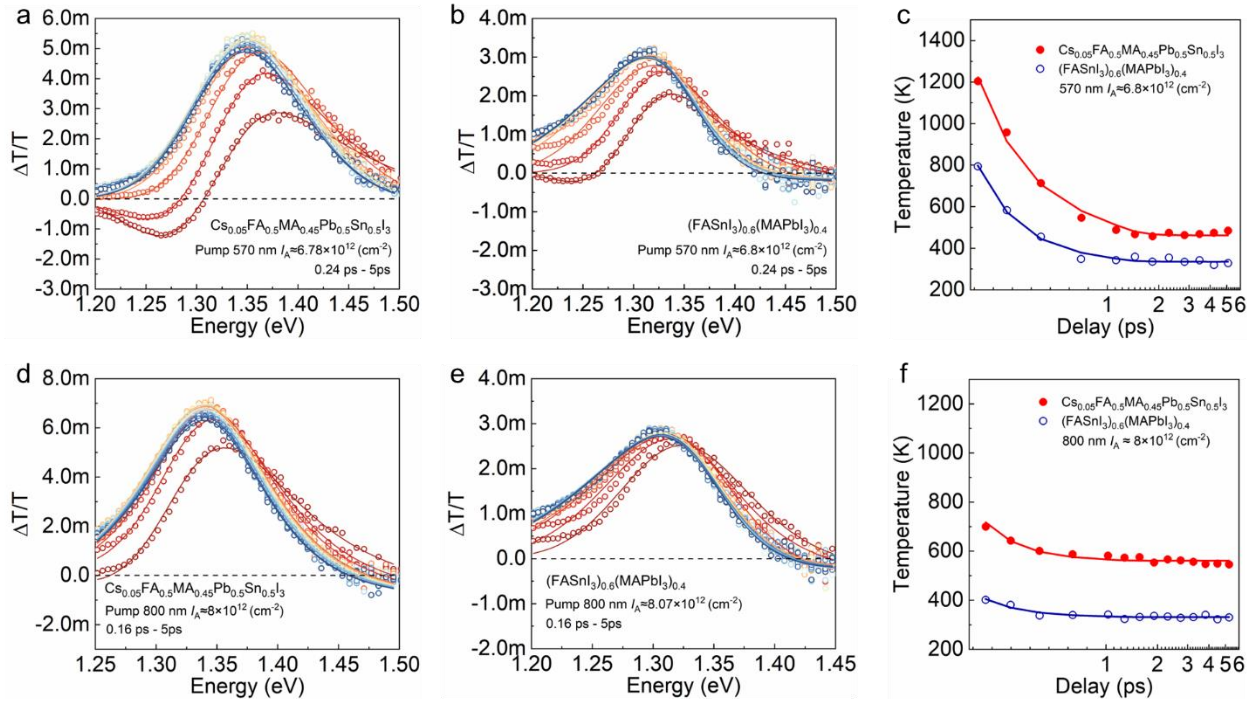

Supplementary Fig. 23| Hot carrier cooling in Pb-Sn mixed perovskite. (a, b). TA spectra of $Cs_{0.05}FA_{0.5}MA_{0.45}Pb_{0.5}Sn_{0.5}I_3$ (a) and $(FASnI_3)_{0.6}(MAPbI_3)_{0.4}$ (b) at different time delays following 2.18 eV pump excitation with absorbed photon flux of ~ 6.8×10^{12} cm^{-2}. **(c).** Calculated cooling temperature under 2.18 eV pump excitation as a function of delay time. **(d, e).** TA spectra of $Cs_{0.05}FA_{0.5}MA_{0.45}Pb_{0.5}Sn_{0.5}I_3$ (d) and $(FASnI_3)_{0.6}(MAPbI_3)_{0.4}$ (e) at different time delays following 1.55 eV pump with absorbed photon flux of ~ 8×10^{12} cm^{-2}. **(f).** Calculated cooling temperature under 1.55 eV pump excitation as a function of delay time.

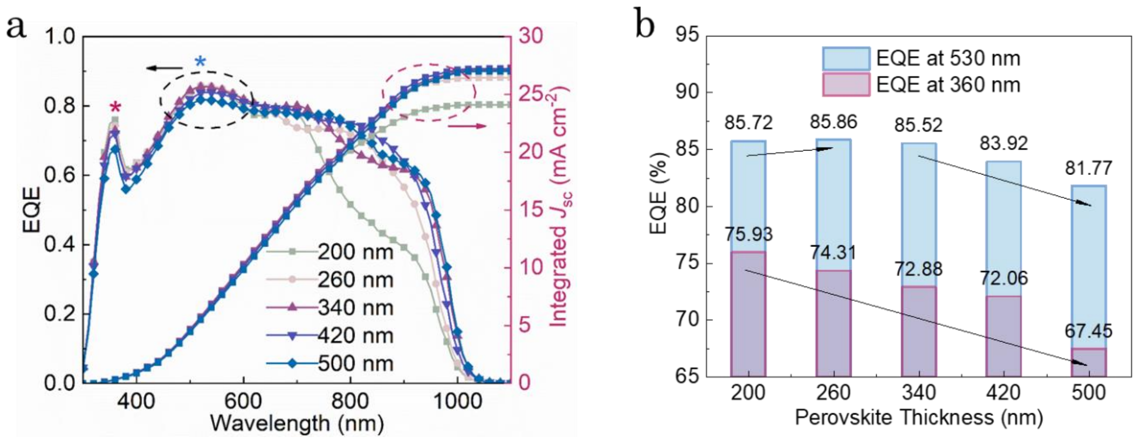

Supplementary Fig. 24| Variation of EQE value with the increase of perovskite thickness. (a). EQE of glass/ITO/PEDOT:PSS/$Cs_{0.05}FA_{0.5}MA_{0.45}Pb_{0.5}Sn_{0.5}I_3$ based PSCs. The perovskite thickness varies from 200 nm to 500 nm. The blue and purple asterisks denote incident photon wavelengths of 530 nm and 360 nm, respectively. **(b).** Variation of EQE excited with high energy photons (360 nm) and relatively low energy photons (530 nm).

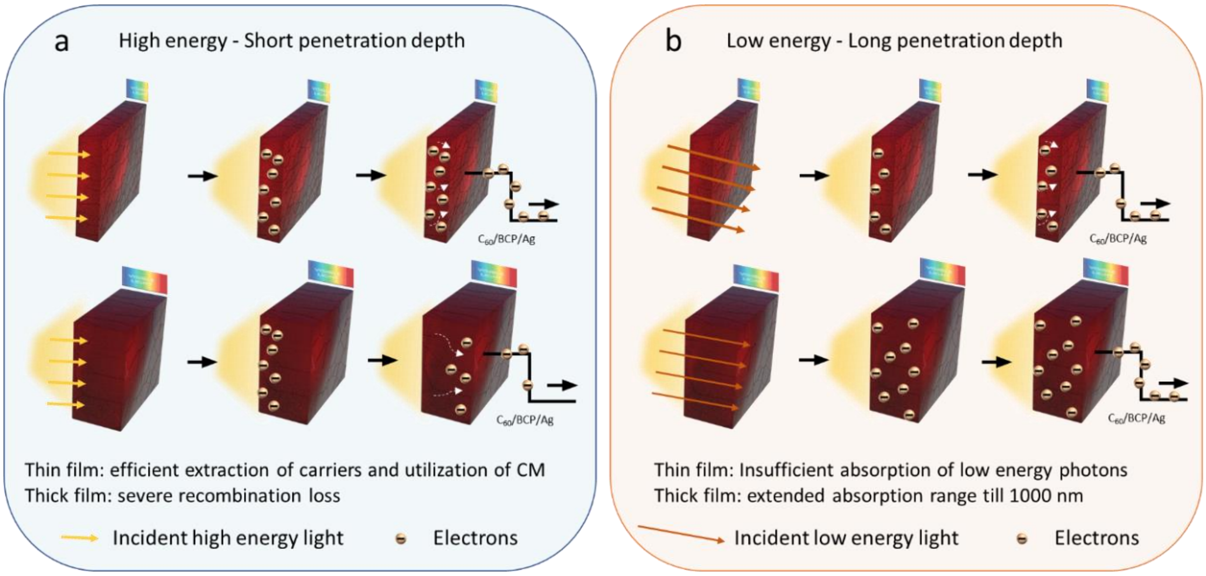

Supplementary Fig. 25| Schematic illumination of carrier extraction in thin and thick perovskite films. (a). For high energy photons with short penetration depth, generated carriers can be transferred and extracted to the external circuit efficiently in thin films but suffer a severe recombination loss during transfer in thick films. Thus, extra carriers generated from CM in high energy region, if any, can be more efficiently extracted from thin perovskite films. **(b).** For low energy photons with long penetration depth, generated carriers can be extracted to the external circuit in both

thin and thick samples. However, the long penetration depth may result in decreased absorption of the low energy photons if the perovskite layer is too thin.

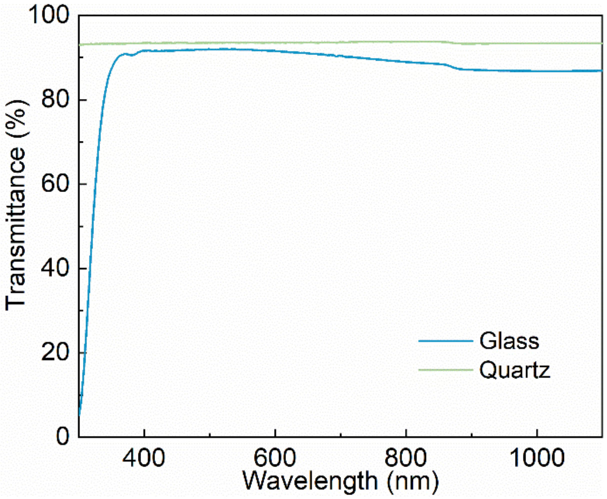

Supplementary Fig. 26| Transmittance of quartz and glass substrates. Air was used as the baseline.

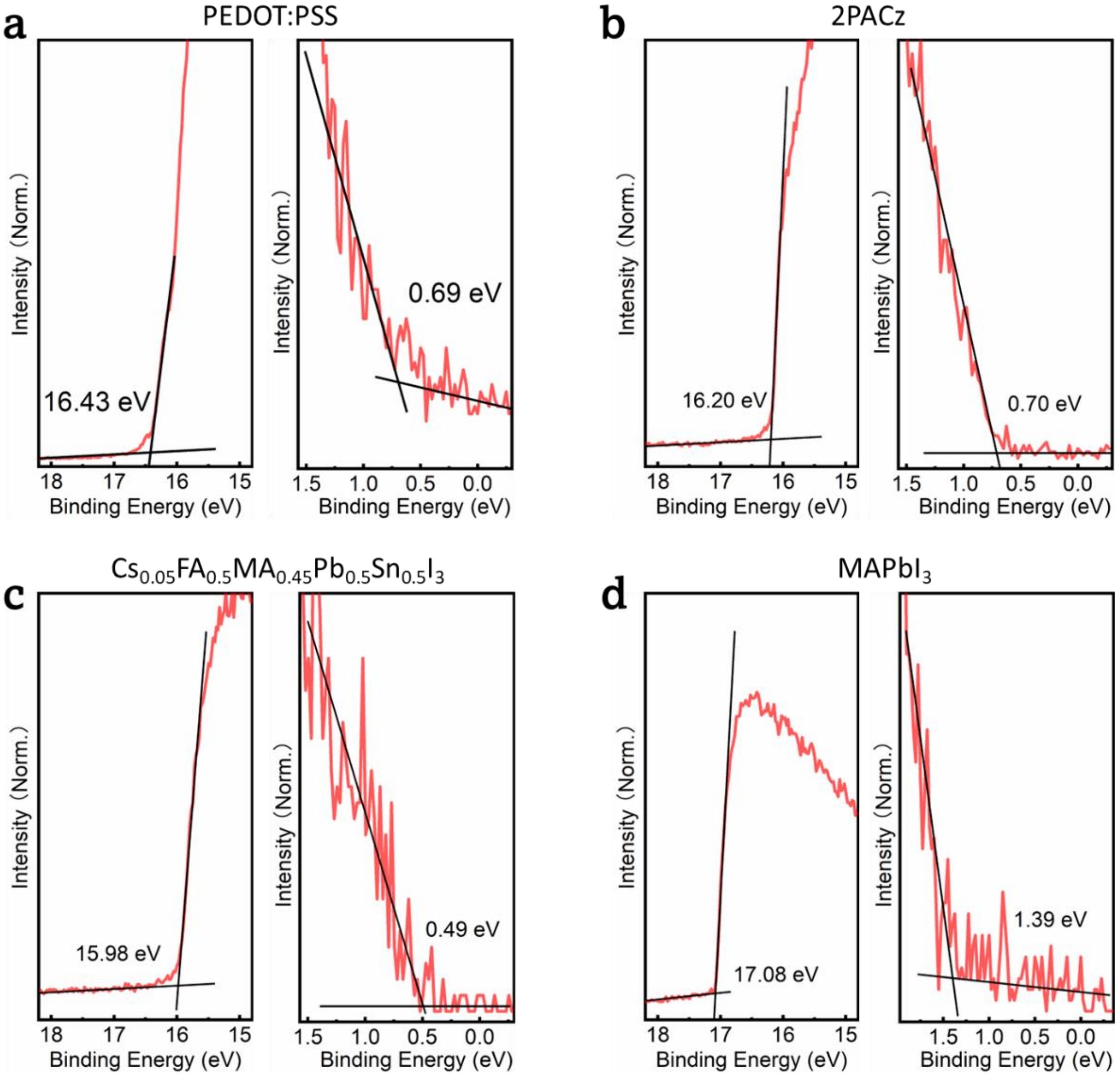

Supplementary Fig. 27| UPS measurements for PEDOT: PSS, 2PACz, $Cs_{0.05}FA_{0.5}MA_{0.45}Pb_{0.5}Sn_{0.5}I_3$ and $MAPbI_3$. Cutoff and onset of **(a).** PEDOT: PSS, (**b**) 2PACz, (**c**) $Cs_{0.05}FA_{0.5}MA_{0.45}Pb_{0.5}Sn_{0.5}I_3$ and **(d).** $MAPbI_3$ perovskite thin films.

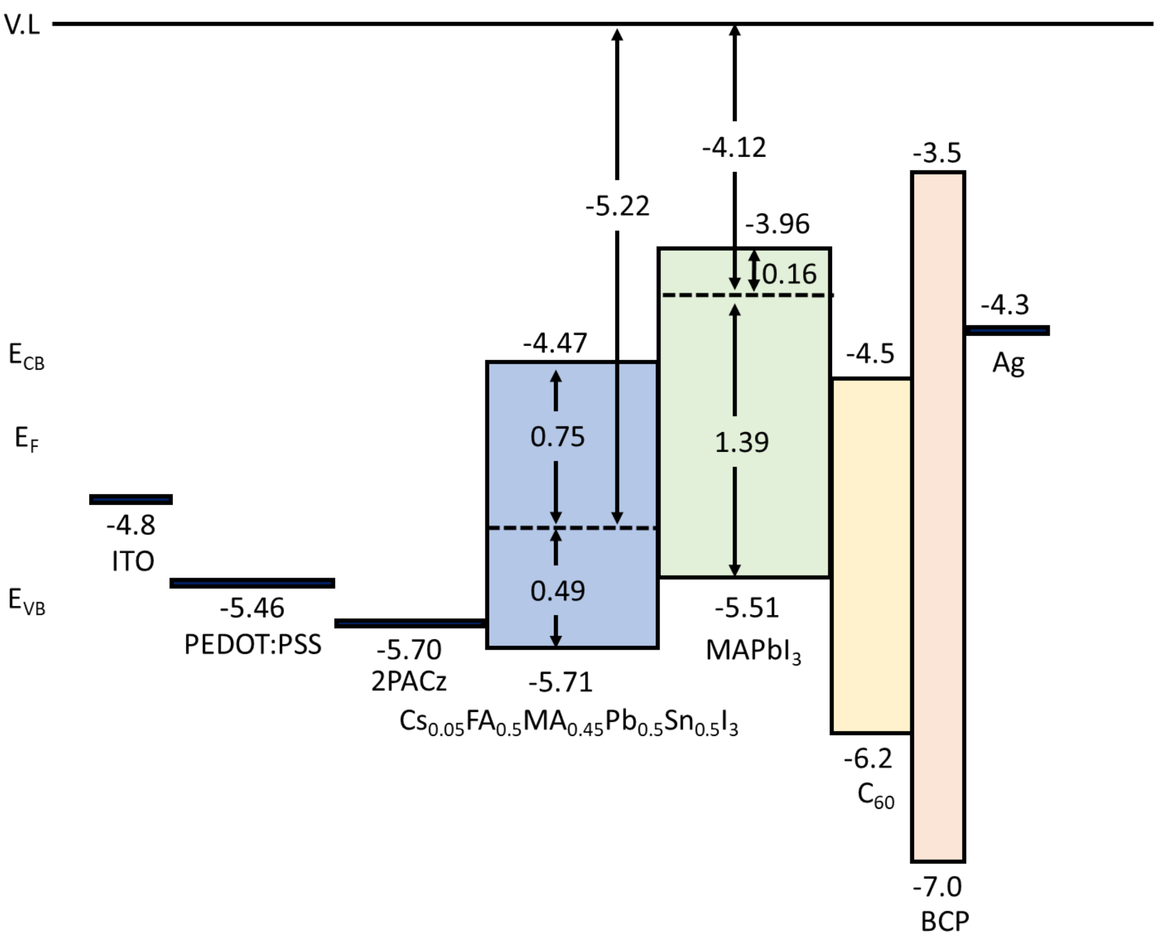

Supplementary Fig. 28| Energy level diagram of the materials in the PSCs.

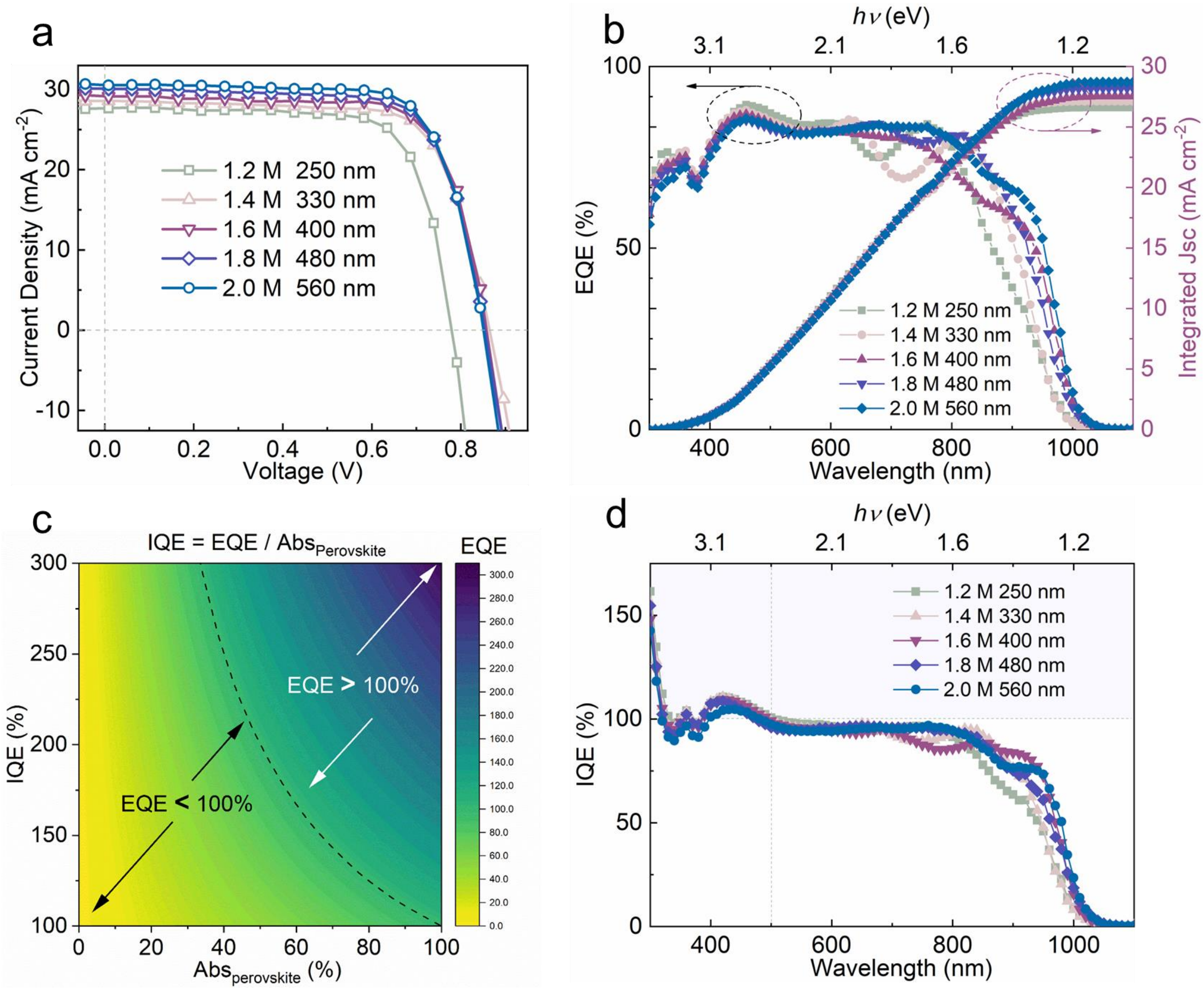

Supplementary Fig. 29| PSC performance under broadband solar illumination. (a). *J*-*V* curves of $Cs_{0.05}FA_{0.5}MA_{0.45}Pb_{0.5}Sn_{0.5}I_3$ PSC devices with different perovskite layer thicknesses measured under one sun. **(b).** The corresponding EQE (left Y-axis) and the integrated J_{sc} (right Y-axis) of PSC devices with different perovskite layer thicknesses. **(c).** Relationship between the EQE, IQE and $Abs_{perovskite}$. The dotted black line is the EQE = 100% boundary. On the left (from yellow to green), the EQE is smaller than 100% although IQE is larger than 100%. The low EQE is due to decreased $Abs_{perovskite}$. On the right (from green to blue), both the EQE and IQE are larger than 100%. **(d).** IQE of the PSCs. The violet shaded region in (d) indicates IQE > 100%.

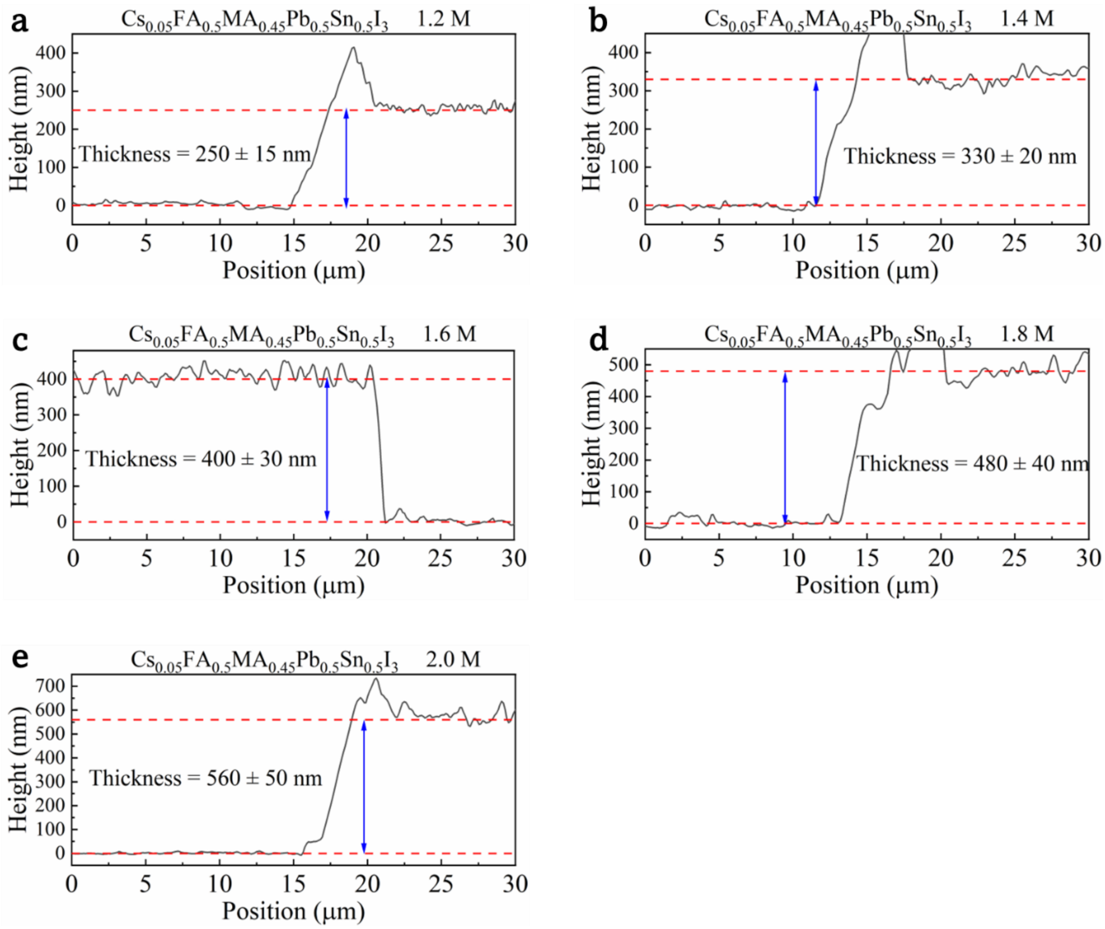

Supplementary Fig. 30| Thickness of the $Cs_{0.05}FA_{0.5}MA_{0.45}Pb_{0.5}Sn_{0.5}I_3$ perovskite layers processed with the vacuum-assisted crystallization method. Precursor concentration: **(a).** 1.2 M, **(b).** 1.4 M, **(c).** 1.6 M, **(d).** 1.8 M, **(e).** 2.0 M.

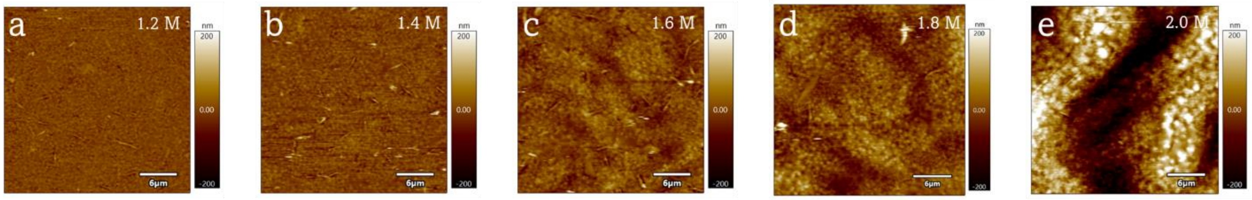

Supplementary Fig. 31| Morphology of the $Cs_{0.05}FA_{0.5}MA_{0.45}Pb_{0.5}Sn_{0.5}I_3$ perovskite layers processed with the vacuum-assisted crystallization method. Precursor concentration: **(a).** 1.2 M, **(b).** 1.4 M, **(c).** 1.6 M, **(d).** 1.8 M, **(e).** 2.0 M.

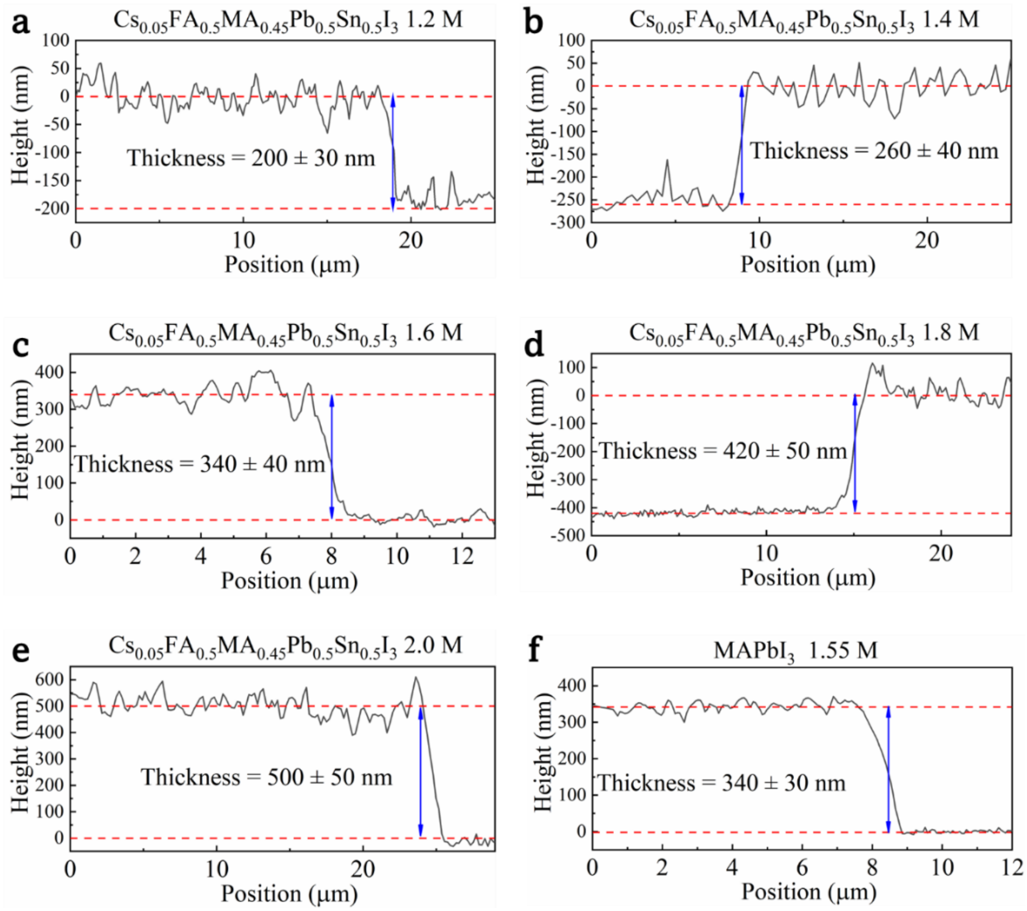

Supplementary Fig. 32| Thickness of the $Cs_{0.05}FA_{0.5}MA_{0.45}Pb_{0.5}Sn_{0.5}I_3$ and $MAPbI_3$ perovskite layers processed without the vacuum-assisted crystallization method. Precursor concentration: **(a).** 1.2 M, **(b).** 1.4 M, **(c).** 1.6 M, **(d).** 1.8 M, **(e).** 2.0 M, **(f).** 1.55M.

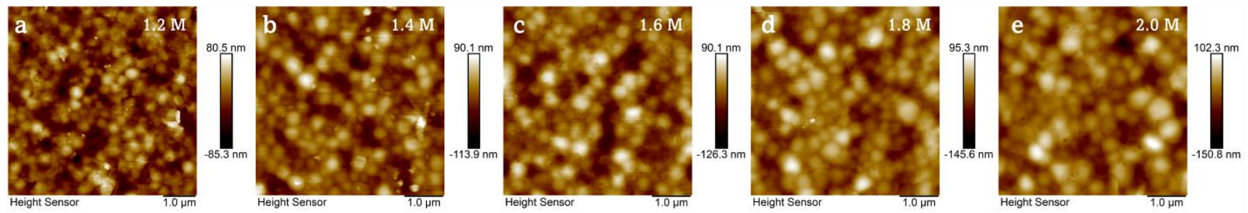

Supplementary Fig. 33| Morphology of the $Cs_{0.05}FA_{0.5}MA_{0.45}Pb_{0.5}Sn_{0.5}I_3$ perovskite layers processed without the vacuum-assisted crystallization method. Precursor concentration: **(a).** 1.2 M, **(b).** 1.4 M, **(c).** 1.6 M, **(d).** 1.8 M, **(e).** 2.0 M.

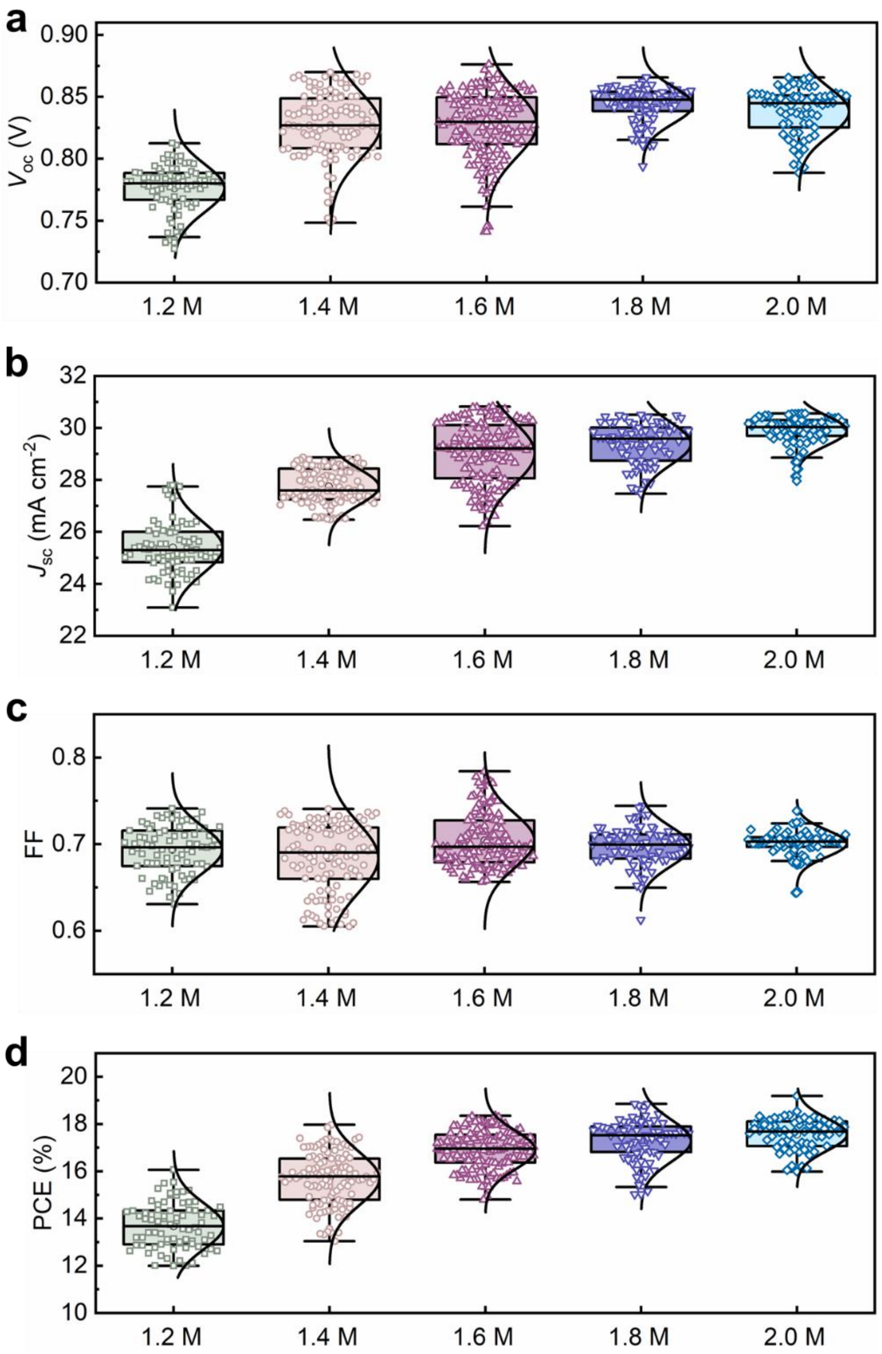

Supplementary Fig. 34| Reproducibility and statistics of $Cs_{0.05}FA_{0.5}MA_{0.45}Pb_{0.5}Sn_{0.5}I_3$ PSCs. (a). V_{oc}. **(b).** J_{sc}. **(c).** FF. **(d).** PCE. For each data set, the box shows the range between the lower quartile to the upper quartile (25%-75%). The top and bottom lines of each data set determine the range within 1.5 times the interquartile range (IQR). The median line represents the middle value (50%).

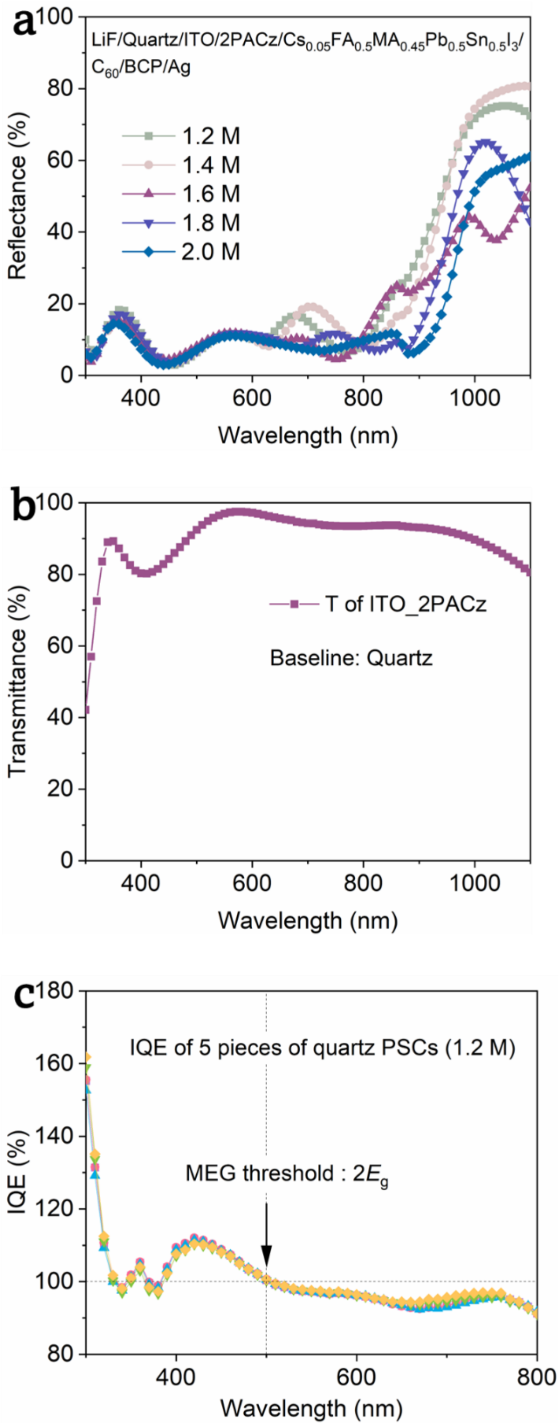

Supplementary Fig. 35| Optical properties of the quartz-based PSCs and the reproducibility of the IQE value. **(a).** Reflectance of PSCs with the structure of LiF/Quartz/ITO/2PACz/Perovskite/C_{60}/BCP/Ag. Perovskite: $Cs_{0.05}FA_{0.5}MA_{0.45}$ $Pb_{0.5}Sn_{0.5}I_3$. 1.2 M-2.0 M. **(b).** Transmittance of ITO_2PACz coated on quartz substrates. **(c).** Reproducibility of IQE for 5 samples of 1.2 M quartz PSCs.

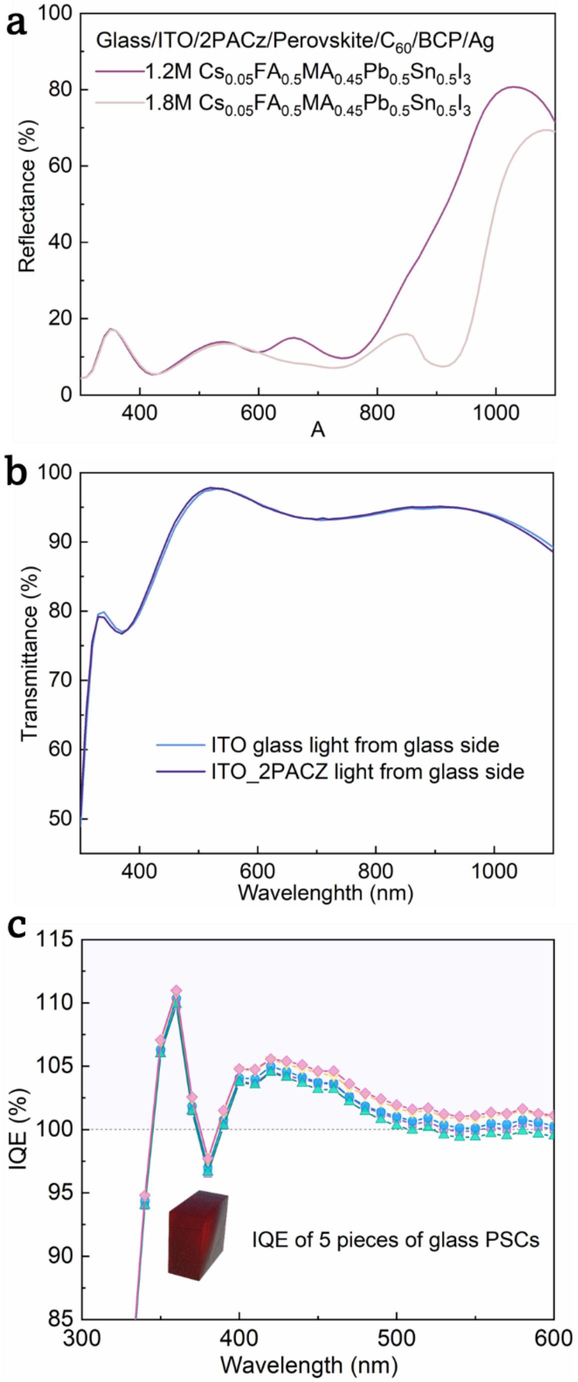

Supplementary Fig. 36| Optical properties of the glass-based PSCs and the reproducibility of the IQE value. **(a).** Reflectance of PSCs with the structure of Glass/ITO/2PACz/Perovskite/C_{60}/BCP/Ag. **(b).** Transmittance of ITO and ITO_2PACz layer coated on glass substrates. **(c).** Reproducibility of IQE for 5 samples of 1.8M $Cs_{0.05}FA_{0.5}MA_{0.45}Pb_{0.5}Sn_{0.5}I_3$ PSCs. The violet shaded region in (c) indicates IQE > 100%.

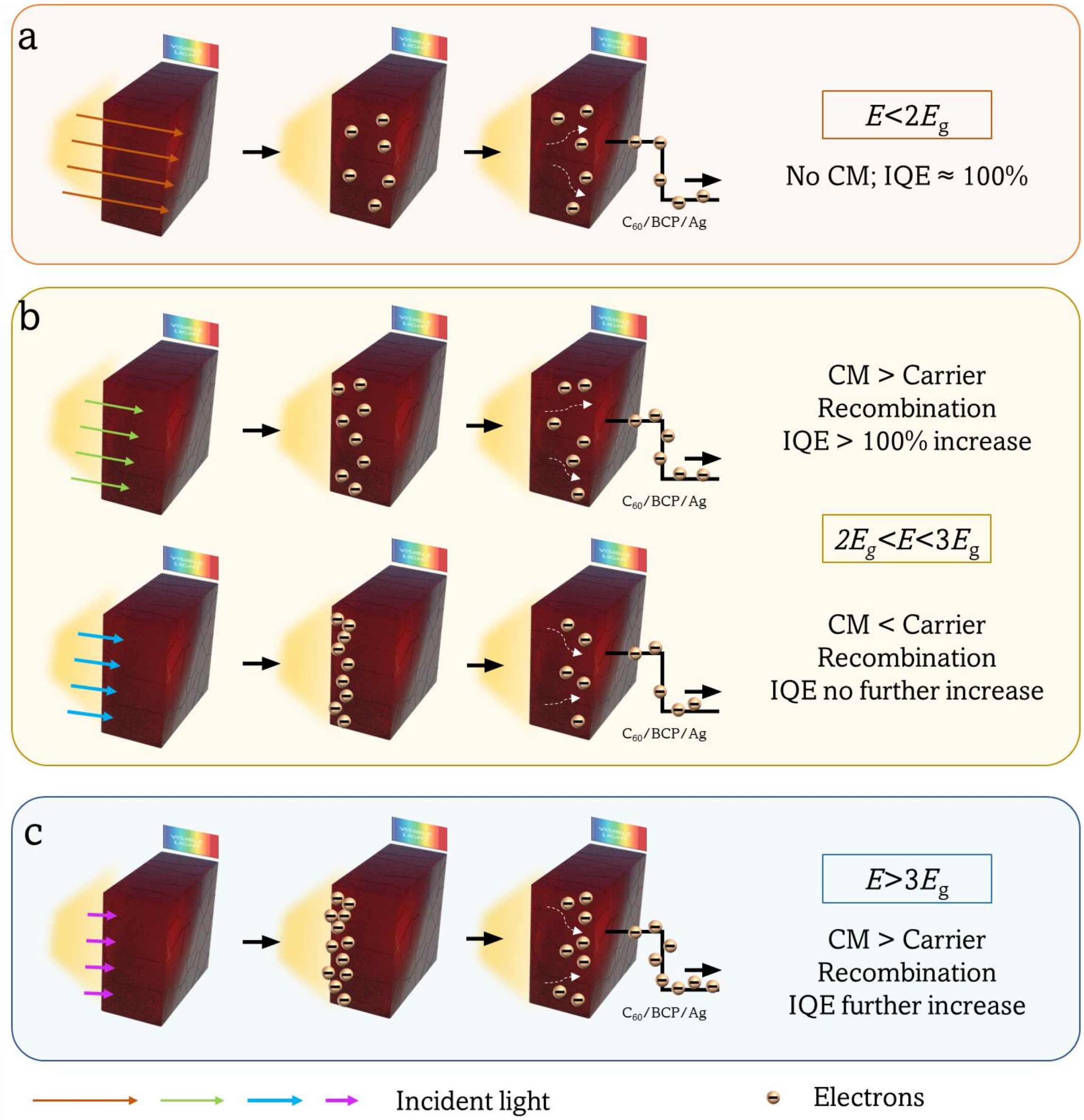

Supplementary Fig. 37| Schematic illustration of carrier extraction and IQE variation with increase of incident photon energy *E*. **(a)** For $E < 2E_g$, there is no CM, IQE approaches100% due to efficient carrier extraction because of the longer penetration depth of lower energy photons. **(b)** For $2E_g < E < 3E_g$, CM competes with carrier recombination due to reduced penetration depth of the high energy photons. If CM dominates carrier recombination, then IQE will exceed 100% and will increase. When CM QY peaks according to the step-like function, the IQE may not increase and could even drop due to carrier recombination losses. **(c)** For $E > 3E_g$, CM QY will increase further and dominate, thus IQE further increases.

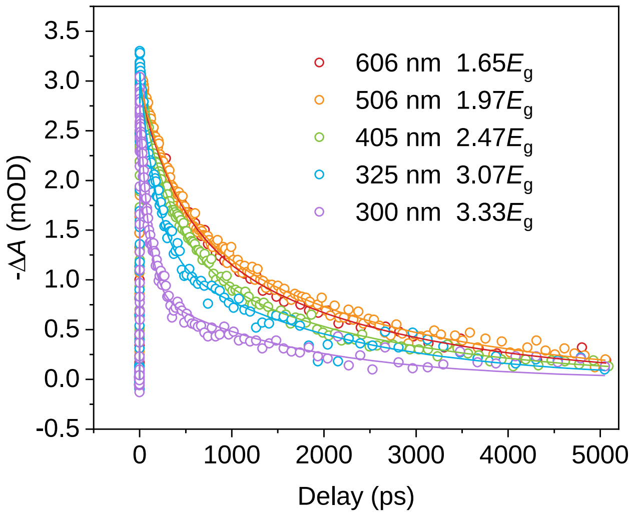

Supplementary Fig. 38| Increased Auger Recombination (AR) in Pb-Sn mixed perovskite with increase of pump energy when the photon energy exceeds 2E_g. When pump photon energy is smaller than 2E_g (i.e., 606 nm and 506 nm), their TA kinetics are similar. When pump photon energy is larger than 2E_g (i.e., 405 nm, 325 nm, and 300 nm), the Auger recombination becomes more severe with the increase of photon energy, even though the pump fluence of the high-energy photon is lower (e.g., the fluence for the 606 nm pump is 2.80 μJ cm^{-2}, while the fluence for the 300 nm pump is 1.65 μJ cm^{-2}).

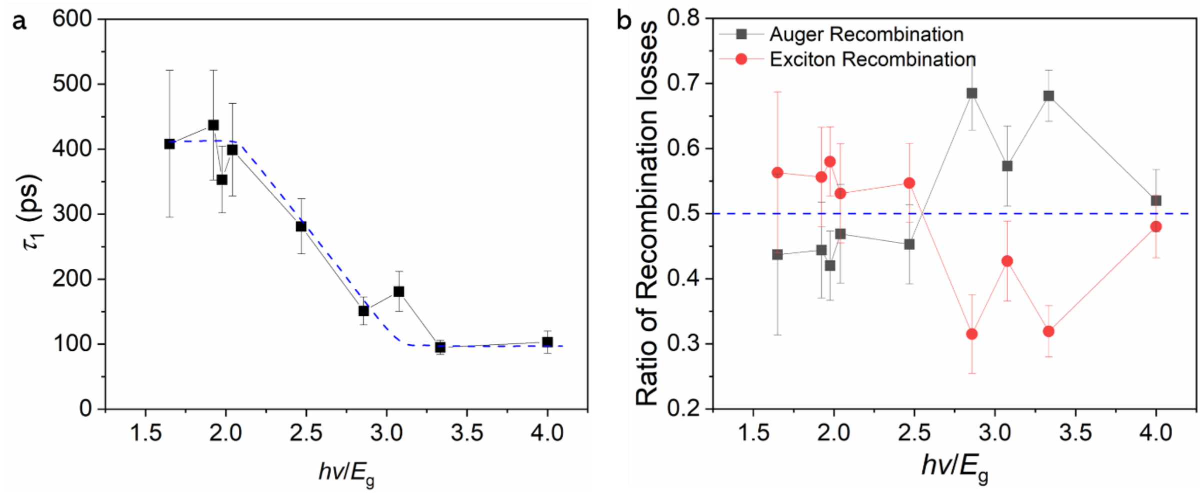

Supplementary Fig. 39| Auger recombination time and Auger recombination ratio as a function of bandgap multiples. (a). Auger recombination time τ_1 as a function of increase of pump energies. (b). Ratio of the Auger recombination and exciton recombination. The error bars represent the uncertainties.

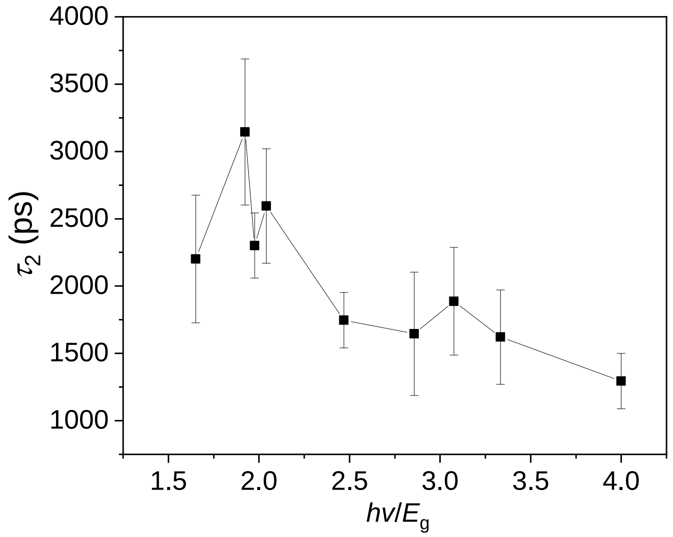

Supplementary Fig. 40| Exciton recombination time τ_2 as a function of increase of pump energies. The error bars represent the uncertainties.

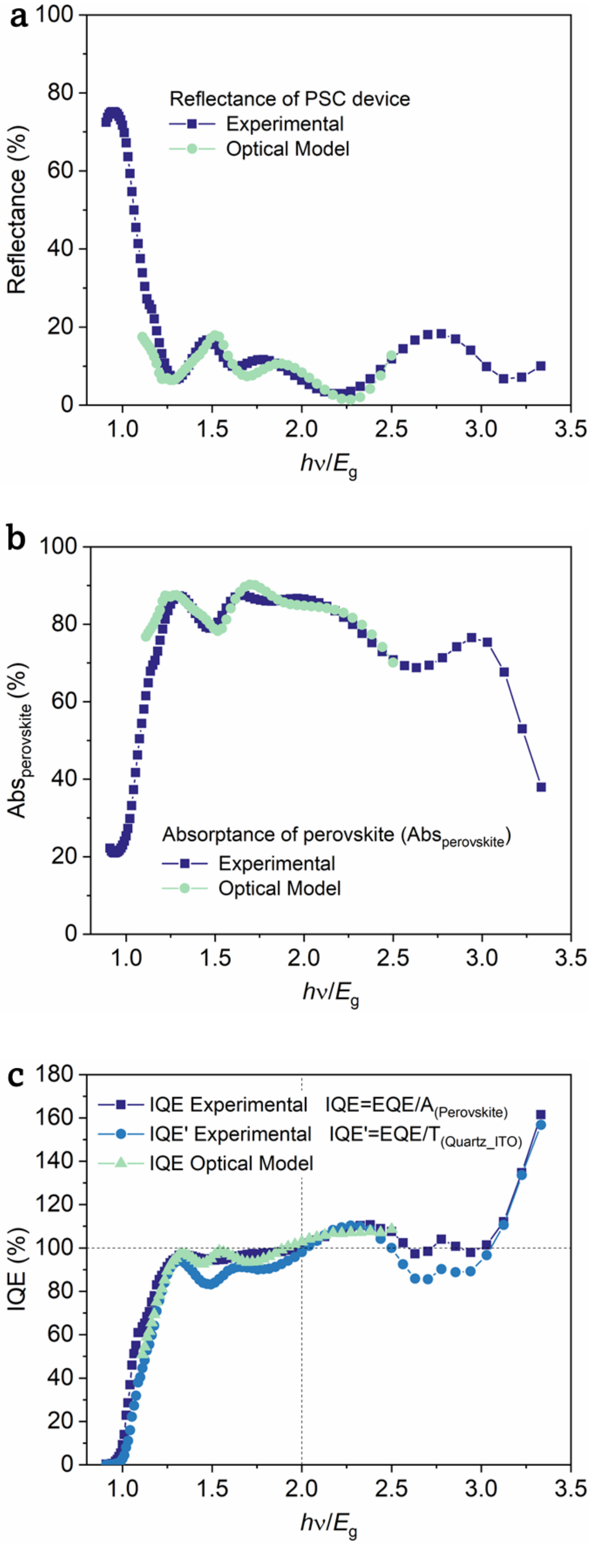

Supplementary Fig. 41| Experimental and simulated *R* of the 1.2 M quartz-ITQ PSC device, Abs$_{perovskite}$ of perovskite layer in a PSC and the corresponding IQE. (a). Experimental and modelled reflectance (*R*) of the PSC

device. **(b).** Experimental and modelled absorptance of the perovskite layer ($Abs_{perovskite}$) in the PSC. **(c).** Experimental and modelled IQE. The purple plot (squares) is the experimental IQE obtained by dividing the EQE with the fraction of light absorbed solely by the perovskite layer in the PSC device. The blue plot (circles) is the experimental IQE obtained by dividing EQE with the initial fraction of light transmitted through the quartz-ITO substrate. The trend of the experimental IQE calculated using both approaches is consistent. The green plot (triangles) is the IQE obtained from optical modelling by the transfer matrix method (TMM) using the *n* and *k* values from ellipsometry measurements. The ellipsometry measurement range is from 381 nm to 893 nm. Hence, the modelled IQE ranges from $1.11E_g$ to $2.5E_g$. The experimental IQE (purple and blue plots) and modelled IQE (green plot) both show an IQE exceeding 100% with a CM threshold of $2E_g$ (at ~ 500nm).

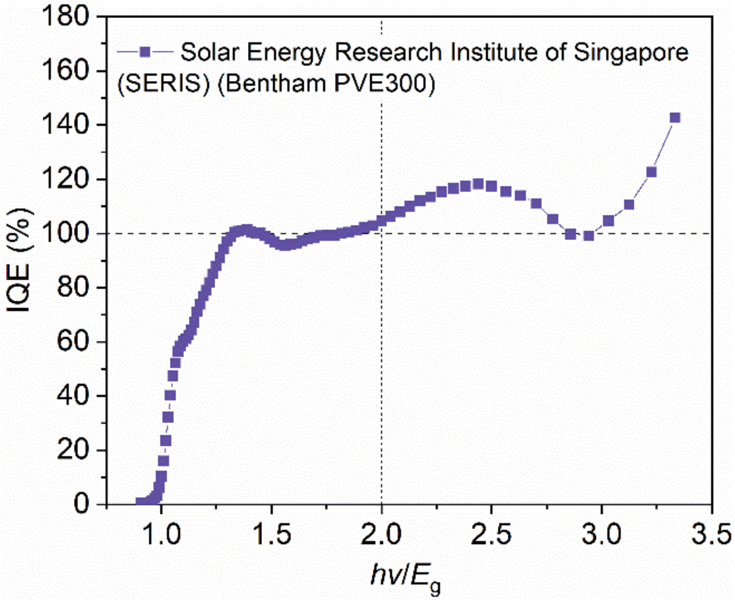

Supplementary Fig. 42| IQE obtained with Bentham PVE300 system in Solar Energy Research Institute of Singapore (SERIS).

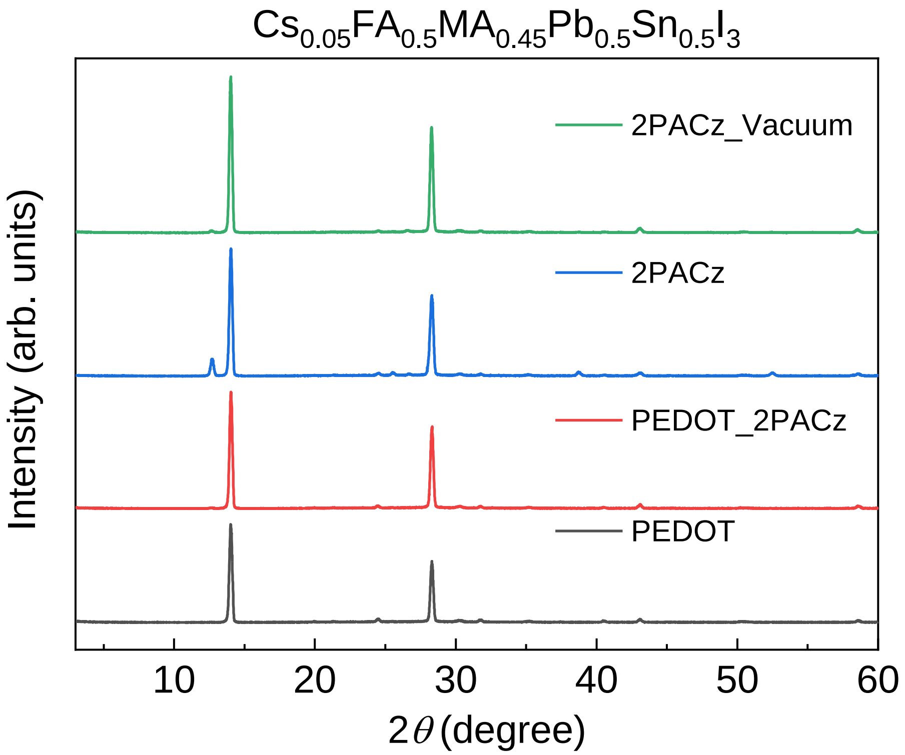

Supplementary Fig. 43| XRD patterns of $Cs_{0.05}FA_{0.5}MA_{0.45}Pb_{0.5}Sn_{0.5}I_3$ on PEDOT: PSS, PEDOT: PSS_2PACz, 2PACz substrates and vacuum pumped $Cs_{0.05}FA_{0.5}MA_{0.45}Pb_{0.5}Sn_{0.5}I_3$ on 2PACz substrate.

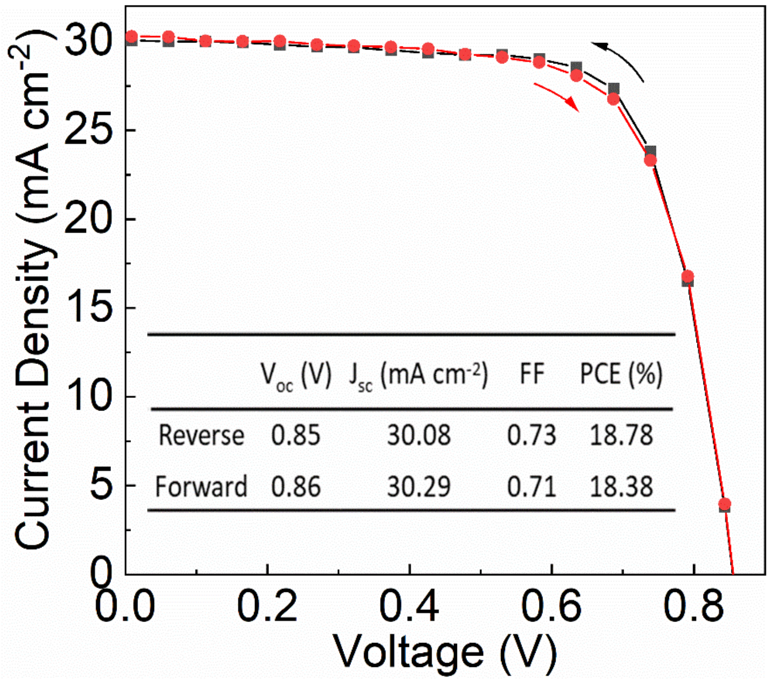

Supplementary Fig. 44| Hysteresis of the $Cs_{0.05}FA_{0.5}MA_{0.45}Pb_{0.5}Sn_{0.5}I_3$ PSC with the structure of LiF/Quartz/ITO/2PACz/Perovskite/C_{60}/BCP/Ag. Black line: reverse scan; Red line: forward scan.

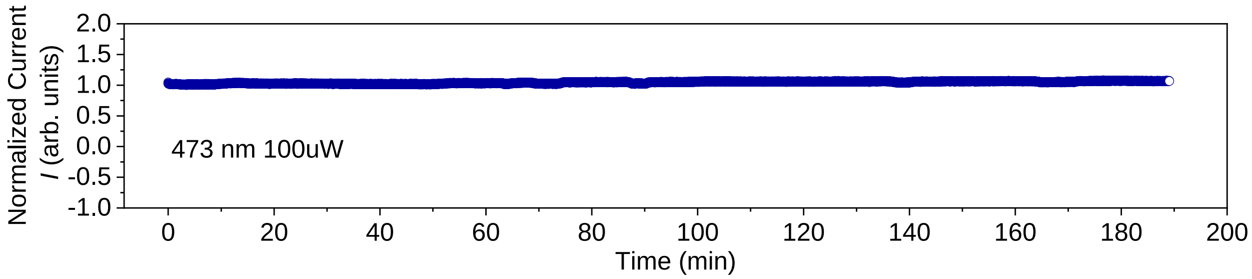

Supplementary Fig. 45| Stability of $Cs_{0.05}FA_{0.5}MA_{0.45}Pb_{0.5}Sn_{0.5}I_3$ PSC with the structure of Quartz/ITO/2PACz/Perovskite/C_{60}/BCP/Ag under 473 nm CW laser illumination.

Supplementary Tables

Supplementary Table 1. The measured J_{sc} from J-V curves and integrated J_{sc} from the EQEs of champion PSC devices with different absorber layer thicknesses.

Concentration	Thickness (nm)	Measured J_{sc} (mA cm^{-2}) (J-V curve)	Integrated J_{sc} (mA cm^{-2}) (EQE)	Relative Discrepancy (%)
1.2 M	250	27.75	26.61	4.1
1.4 M	330	28.57	27.13	5.0
1.6 M	400	29.16	27.54	5.6
1.8 M	480	30.08	28.35	5.7
2.0 M	560	30.55	28.70	6.1

Supplementary Table 2. Statistics of photovoltaic parameters for PSCs based on different perovskite layer thickness.

Concentration	Thickness (nm)	V_{oc} (V)	J_{sc} (mA cm^{-2})	FF (%)	PCE (%)
1.2 M	250	0.78 ± 0.02	25.4 ± 1.0	69.4 ± 2.7	13.7 ± 1.0
1.4 M	330	0.83 ± 0.03	27.7 ± 0.7	68.4 ± 4.0	15.7 ± 1.1
1.6 M	400	0.83 ± 0.03	29.0 ± 1.2	70.4 ± 3.1	16.9 ± 0.8
1.8 M	480	0.84 ± 0.01	29.4 ± 0.8	69.7 ± 2.3	17.3 ± 0.9
2.0 M	560	0.84 ± 0.02	29.9 ± 0.6	70.1± 1.5	17.6 ± 0.7

The data include average values and standard deviations calculated from at least 65 separate devices for each concentration.

Supplementary Table 3. Parameters of the bi-exponential decay.

	A_1 ($\times 10^{-4}$)	τ_1 (ps)	A_2 ($\times 10^{-4}$)	τ_2 (ps)	A_1 ratio (%)	A_2 ratio (%)
606 nm 1.65E_g	13 ± 4	410 ± 110	16 ± 4	2200 ± 500	44 ± 10	56 ± 10
520 nm 1.92E_g	14 ± 2	440 ± 80	17 ± 2	3100 ± 500	44 ± 7	56 ± 7
506 nm 1.98E_g	13 ± 2	350 ± 50	17 ± 2	2300 ± 200	42 ± 5	58 ± 5
490 nm 2.04E_g	14 ± 2	400 ± 70	16 ± 2	2600 ± 400	47 ± 8	53 ± 8
405 nm 2.47E_g	13 ± 2	280 ± 40	16 ± 2	1700 ± 200	45 ± 6	55 ± 6
350 nm 2.86E_g	20 ± 2	150 ± 20	9 ± 2	1600 ± 500	69 ± 6	32 ± 6
325 nm 3.08E_g	18 ± 2	180 ± 30	13 ± 2	1900 ± 400	57 ± 6	43 ± 6
300 nm 3.33E_g	19 ± 1	100 ±10	9 ± 1	1600 ± 400	68 ± 4	32 ± 4
250 nm 4.00E_g	15 ± 1	100 ±20	14 ± 1	1300 ± 200	52 ± 5	48 ± 5

The data were fitted by bi-exponential decay convolved with a gaussian function, as described in Supplementary Note 11.

Supplementary References

1 Schaller, R. D. & Klimov, V. I. High efficiency carrier multiplication in PbSe nanocrystals: implications for solar energy conversion. *Phys Rev Lett* **92**, 186601 (2004).

2 Beard, M. C. Multiple exciton generation in semiconductor quantum dots. *J Phys Chem Lett* **2**, 1282-1288 (2011).

3 Nozik, A. J. Multiple exciton generation in semiconductor quantum dots. *Chemical Physics Letters* **457**, 3-11 (2008).

4 Li, M. et al. Low threshold and efficient multiple exciton generation in halide perovskite nanocrystals. *Nat Commun* **9**, 4197 (2018).

5 Tyagi, P. & Kambhampati, P. False multiple exciton recombination and multiple exciton generation signals in semiconductor quantum dots arise from surface charge trapping. *J Chem Phys* **134**, 094706 (2011).

6 Klimov, V. I. Spectral and dynamical properties of multiexcitons in semiconductor nanocrystals. *Annu Rev Phys Chem* **58**, 635-673 (2007).

7 Beard, M. C. et al. Comparing multiple exciton generation in quantum dots to impact ionization in bulk semiconductors: implications for enhancement of solar energy conversion. *Nano Lett* **10**, 3019-3027 (2010).

8 Ridley, B. K. *Quantum Processes in Semiconductors*. (Oxford University Press, Oxford, 2013).

9 Verma, S. D., Gu, Q., Sadhanala, A., Venugopalan, V. & Rao, A. Slow carrier cooling in hybrid pb–sn halide perovskites. *ACS Energy Letters* **4**, 736-740 (2019).

10 Maiti, S. et al. Emergence of new materials for exploiting highly efficient carrier multiplication in photovoltaics. *Chemical Physics Reviews* **1**, 011302 (2020).

11 Spoor, F. C. M. et al. Asymmetric optical transitions determine the onset of carrier multiplication in lead chalcogenide quantum confined and bulk crystals. *ACS Nano* **12**, 4796-4802 (2018).

12 Blochl, P. E. Projector augmented-wave method. *Physical Review B* **50**, 17953-17979 (1994).

13 Lee, K., Murray, E. D., Kong, L. Z., Lundqvist, B. I. & Langreth, D. C. Higher-accuracy van der Waals density functional. *Physical Review B* **82**, 081101 (2010).

14 Perdew, J. P., Burke, K. & Ernzerhof, M. Generalized gradient approximation made simple. *Physical Review Letters* **77**, 3865-3868 (1996).

15 Kresse, G. & Furthmuller, J. Efficient iterative schemes for ab initio total-energy calculations using a plane-wave basis set. *Physical Review B* **54**, 11169-11186 (1996).

16 Monkhorst, H. J. & Pack, J. D. Special points for brillouin-zone integrations. *Physical Review B* **13**, 5188-5192 (1976).

17 Semonin, O. E. et al. Peak external photocurrent quantum efficiency exceeding 100% via MEG in a quantum dot solar cell. *Science* **334**, 1530-1533 (2011).

18 Burkhard, G. F., Hoke, E. T. & McGehee, M. D. Accounting for interference, scattering, and electrode absorption to make accurate internal quantum efficiency measurements in organic and other thin solar cells. *Advanced Materials* **22**, 3293-3297 (2010).

19 Lim, J. W. M. et al. Hot carriers in halide perovskites: how hot truly? *J Phys Chem Lett* **11**, 2743-2750 (2020).

20 Klimov, V. V., Haring Bolivar, P. & Kurz, H. Hot-phonon effects in femtosecond luminescence spectra of electron-hole plasmas in CdS. *Phys Rev B Condens Matter* **52**, 4728-4731 (1995).

21 Li, M. J. et al. Slow cooling and highly efficient extraction of hot carriers in colloidal perovskite nanocrystals. *Nature Communications* **8**, 14350 (2017).

22 Wang, T. et al. Protecting hot carriers by tuning hybrid perovskite structures with alkali cations. *Science Advances* **6**, eabb1336 (2020).

23 Alonso-Álvarez, D. et al. Solcore: a multi-scale, Python-based library for modelling solar cells and semiconductor materials. *Journal of Computational Electronics* **17**, 1099-1123 (2018).
