# Supplementary material for: Carrier multiplication in perovskite solar cells with internal quantum efficiency exceeding 100%
Source: Nat Commun. 2023 Oct 9;14:6293. doi: 10.1038/s41467-023-41758-w (PMC10562407; doi:10.1038/s41467-023-41758-w)
Supplement: Supplementary file 3 — Reporting Summary [file 41467_2023_41758_MOESM3_ESM.pdf]

nature portfolio

Corresponding author(s): Tze Chien Sum

Solar Cells Reporting Summary

Nature Portfolio wishes to improve the reproducibility of the work that we publish. This form is intended for publication with all accepted papers reporting the characterization of photovoltaic devices and provides structure for consistency and transparency in reporting. Some list items might not apply to an individual manuscript, but all fields must be completed for clarity.

For further information on Nature Research policies, including our data availability policy, see Authors & Referees.

Experimental design

Please check the following details are reported in the manuscript, and provide a brief description or explanation where applicable.

1. Dimensions

Area of the tested solar cells

☒ Yes
☐ No

For J-V curves measured under one sun condition, the area of the tested solar cells is 0.06 cm^2. See details in Experimental Methods.
For J-V curves measured under monochromatic illumination CW laser, the area of the tested solar cells is 0.09 cm^2. The active area of the solar cell device is determined by the beam size of laser beam, which is smaller than the area of the tested solar cell. See details in Experimental Methods.

Explain why this information is not reported/not relevant.

Method used to determine the device area

☒ Yes
☐ No

The active area of solar cell measured under monochromatic illumination is determined by the beam size of the CW laser, because the beam size of the CW laser is smaller than the area of the solar cell device. The beam size is measured with a CMOS camera (Thorlabs, DCC1545M-GL) and the diameter is defined as the 1 e^(-2) of the maximum intensity, with the assumption of Gaussian beam shape. See details in Experimental Methods.

Explain why this information is not reported/not relevant.

2. Current-voltage characterization

Current density-voltage (J-V) plots in both forward and backward direction

☒ Yes
☐ No

See Supplementary Fig. 44.

Voltage scan conditions

☒ Yes
☐ No

The J–V curves were measured from 1.0 V to −0.2 V at a scan rate of ~0.5 V s^(−1). See details in Experimental Methods.

Explain why this information is not reported/not relevant.

Test environment

☒ Yes
☐ No

For J-V curves measured under one sun condition, the measurements were conducted in a N2-filled glovebox (O2 < 10 ppm, H2O < 1.0 ppm) at room temperature. See details in Experimental Methods.
For J-V curves measured under monochromatic CW laser, The measurements were conducted in air at room temperature and the solar cells were encapsulated in the N2-filled glovebox in advance. See details in Experimental Methods.

Explain why this information is not reported/not relevant.

Protocol for preconditioning of the device before its characterization

☐ Yes
☒ No

Provide a description of the protocol.

No preconditioning processes for the solar cells.

Stability of the J-V characteristic

☒ Yes
☐ No

Stability of the PSCs under 473 nm CW laser illumination can be found in Supplementary Fig. 45.

Explain why this information is not reported/not relevant.

3. Hysteresis or any other unusual behaviour

Description of the unusual behaviour observed during the characterization

☒ Yes
☐ No

Negligible hysteresis is observed.

Explain why this information is not reported/not relevant.

Related experimental data	☒ Yes<br>☐ No	See Supplementary Fig. 44.<br>*Explain why this information is not reported/not relevant.*

4. Efficiency

External quantum efficiency (EQE) or incident photons to current efficiency (IPCE)	☒ Yes<br>☐ No	The EQE spectra can be found in Figure 3b, Supplementary Fig. 24, Supplementary Fig. 29b.<br>*Explain why this information is not reported/not relevant.*
A comparison between the integrated response under the standard reference spectrum and the response measure under the simulator	☒ Yes<br>☐ No	The relative discrepancy between the integrated response under the standard reference spectrum and the response measured under the simulator is less than ~6%. See Supplementary Table 1.<br>*Explain why this information is not reported/not relevant.*
For tandem solar cells, the bias illumination and bias voltage used for each subcell	☐ Yes<br>☒ No	*Provide a description of the measurement conditions.*<br>No tandem solar cells involved.

5. Calibration

Light source and reference cell or sensor used for the characterization	☒ Yes<br>☐ No	The used light source is a commercial solar simulator (model: UHE-NSC) from ScienceTech Inc. with certified AAA grade. The used reference cell is a calibrated Oriel PV Reference Cell System (91150V) certified by Newport Co.<br>*Explain why this information is not reported/not relevant.*
Confirmation that the reference cell was calibrated and certified	☒ Yes<br>☐ No	The used reference cell is a calibrated Oriel PV Reference Cell System (91150V) certified by Newport Co.<br>*Explain why this information is not reported/not relevant.*
Calculation of spectral mismatch between the reference cell and the devices under test	☒ Yes<br>☐ No	The spectral mismatch was calibrated according to previous publication (Reference 39).<br>*Explain why this information is not reported/not relevant.*

6. Mask/aperture

Size of the mask/aperture used during testing	☐ Yes<br>☒ No	*Report the size of the mask/aperture.*<br>No Aperture was used during the test. The size of laser beam is smaller than the size of solar cells.
Variation of the measured short-circuit current density with the mask/aperture area	☐ Yes<br>☒ No	*Report the difference in the short-circuit current density values measured with the mask and aperture area.*<br>No Aperture was used during the test. The size of laser beam is smaller than the size of solar cells.

7. Performance certification

Identity of the independent certification laboratory that confirmed the photovoltaic performance	☐ Yes<br>☒ No	*Identify the independent certification laboratory.*<br>A record-breaking high performance of the solar cells is not the focus of this research.
A copy of any certificate(s)	☐ Yes<br>☒ No	*Certificate copies should be provided in the Supplementary information. Please state the supplementary item number.*<br>A record-breaking high performance of the solar cells is not the focus of this research.

8. Statistics

Number of solar cells tested	☒ Yes<br>☐ No	For statistical analysis of device performance, more than 65 devices were tested. See Supplementary Fig. 34 and Supplementary Table 2.<br>*Explain why this information is not reported/not relevant.*
Statistical analysis of the device performance	☒ Yes<br>☐ No	The statistical data were presented in Supplementary Fig. 34 and Supplementary Table 2.<br>*Explain why this information is not reported/not relevant.*

9. Long-term stability analysis

Type of analysis, bias conditions and environmental conditions	☐ Yes<br>☒ No	*Provide a description of the type of analysis, bias conditions and environmental conditions (e.g. illumination type, temperature, atmosphere humidity, encapsulation method, preconditioning temperature, bias) for each long-term stability analysis carried out; see ref. 7 and 8 for details.*<br>Long-term stability is not the focus of this research.
